# The GATA factor ELT-3 specifies endoderm in *Caenorhabditis angaria* in an ancestral gene network

**DOI:** 10.1242/dev.200984

**Published:** 2022-10-24

**Authors:** Gina Broitman-Maduro, Simo Sun, Taisei Kikuchi, Morris F. Maduro

**Affiliations:** ^1^ Department of Molecular, Cell and Systems Biology, University of California, Riverside, CA 92521, USA; ^2^ Department of Infectious Diseases, Faculty of Medicine, University of Miyazaki, 5200 Kihara, Miyazaki 889-1692, Japan; ^3^ Department of Integrated Biosciences, Graduate School of Frontier Sciences, The University of Tokyo, Chiba 277-8562, Japan

**Keywords:** *Caenorhabditis*, GATA factors, Endoderm specification, Gene network evolution, ELT-3, Developmental system drift

## Abstract

Endoderm specification in *Caenorhabditis elegans* occurs through a network in which maternally provided SKN-1/Nrf, with additional input from POP-1/TCF, activates the GATA factor cascade MED-1,2→END-1,3→ELT-2,7. Orthologues of the MED, END and ELT-7 factors are found only among nematodes closely related to *C. elegans*, raising the question of how gut is specified in their absence in more distant species in the genus. We find that the *C. angaria*, *C. portoensis* and *C. monodelphis* orthologues of the GATA factor gene *elt-3* are expressed in the early E lineage, just before their *elt-2* orthologues. In *C. angaria*, *Can-pop-1(RNAi)*, *Can-elt-3(RNAi)* and a *Can-elt-3* null mutation result in a penetrant ‘gutless’ phenotype. *Can-pop-1* is necessary for *Can-elt-3* activation, showing that it acts upstream*.* Forced early E lineage expression of *Can-elt-3* in *C. elegans* can direct the expression of a *Can-elt-2* transgene and rescue an *elt-7 end-1 end-3; elt-2* quadruple mutant strain to viability. Our results demonstrate an ancestral mechanism for gut specification and differentiation in *Caenorhabditis* involving a simpler POP-1→ELT-3→ELT-2 gene network.

## Teaser

**Highlighted Article:** Specification of the gut progenitor E in a distant relative of *Caenorhabditis elegans* uses the GATA factor ELT-3, suggesting that the ancestral network was simpler.

## INTRODUCTION

Gene regulatory networks drive development in metazoan systems and are subject to large- and small-scale changes over evolutionary time (True and Haag, 2001; Davidson and Levine, 2008). Two important mechanisms driving changes in developmental gene networks are changes in *cis-*regulation (rewiring) and gene duplication followed by subfunctionalization. One example of *cis-*regulatory changes occurs in endomesoderm specification in echinoderms through differences in responsiveness to the T-box factor *Tbrain* (Hinman et al., 2007). In another example, the MADF-BESS family is specifically amplified to 16 genes in *Drosophila* where the derived paralogues play overlapping roles in wing hinge development (Shukla et al., 2014). In an example of both duplication and rewiring, the *Drosophila* anterior embryo specification factor *bicoid*, found only in Cyclorrhaphan flies, arose through duplication of an ancient Hox gene followed by changes in expression and loss of the role of the ancestral factor (Stauber et al., 1999, 2002). Such cases in which the outward phenotype is maintained constitute examples of what is known as developmental system drift (True and Haag, 2001).

**Fig. 1.**
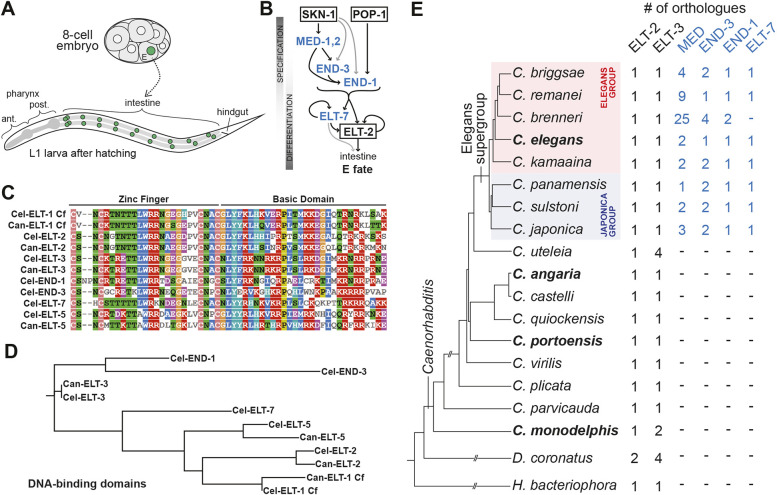
**The E blastomere, the core endoderm gene network, and GATA factor conservation in *Caenorhabditis*.** (A) The E cell, shown at the eight-cell stage, gives rise to 20 descendants that form the juvenile intestine, shown in a larva. The nuclei of E and its descendants are shaded green. The remainder of the digestive tract is also shown. ant., anterior; post., posterior. (B) Diagram of the endoderm specification network from *C. elegans* (Maduro, 2017). The factors that are absent in more distant relatives of *C. elegans* are shaded in blue. Black lines indicate strong regulatory interactions, and gray lines indicate weaker interactions. (C) Alignment of the DBDs (C4 zinc finger and basic domain) of the canonical GATA factors in *C. elegans* and *C. angaria*. The coloured blocks were generated by MView Multiple Sequence Alignment (https://www.ebi.ac.uk/Tools/msa/mview/). (D) RAxML-NG tree of the DBDs shown in C generated using CIPRES Gateway (https://www.phylo.org/), similar to trees made in a prior work (Eurmsirilerd and Maduro, 2020). The *C. elegans* genome contains two additional embryonic GATA factors, *elt-4*, which is a partial duplication of *elt-2* that lacks function, and *elt-6*, a paralogue of *elt-5* (Koh and Rothman, 2001; Fukushige et al., 2003). (E) Phylogeny of *C. elegans* with two outgroup species, *Diploscapter coronatus* and *Heterorhabditis bacteriophora*, based on previously published work and the most recent phylogeny available from The Caenorhabditis Genomes Project (Félix et al., 2014; Slos et al., 2017; Stevens et al., 2019; Stevens et al., 2020). For *C. uteleia*, the *elt-2* orthologue is CUTEL.g25177 and the *elt-3* orthologues are CUTEL.g19098, CUTEL.g19099, CUTEL.g14171 and CUTEL.g17053 (assembly JU2585_v1 from The Caenorhabditis Genomes Project). For *C. portoensis*, the *elt-2* orthologue is CPORT.g4338 and the *elt-3* orthologue is CPORT.g6550 (assembly EG5626_v1 from The Caenorhabditis Genomes Project). All remaining orthologues were identified previously (Eurmsirilerd and Maduro, 2020; Maduro, 2020). Species studied in this work are in bold.

One of the most-studied gene networks in animals is that which specifies the *Caenorhabditis elegans* gut cell progenitor E and promotes intestine differentiation in its descendants (Fig. 1A,B). The zygotic portion of this network consists of a cascade of structurally similar transcription factors that are found only among close relatives of *C. elegans*, suggesting that they may be the result of duplication and subfunctionalization (Eurmsirilerd and Maduro, 2020; Maduro, 2020). At the top of the network, the maternal SKN-1/Nrf factor, acting partially through its zygotic effectors MED-1,2, along with the maternal Wnt/β-catenin asymmetry pathway through its effector POP-1/TCF, activate early E lineage expression of the *end-3* and *end-1* genes (Bowerman et al., 1992; Lin et al., 1995; Rocheleau et al., 1997; Thorpe et al., 1997; Maduro et al., 2001, 2002; Shetty et al., 2005; Bhambhani et al., 2014). Downstream of these transiently expressed factors, *elt-2* and its paralogue *elt-7* drive gut development and differentiation, and their expression is maintained through adulthood (Fukushige et al., 1998; Sommermann et al., 2010; Dineen et al., 2018).

As might be expected for a network with structurally similar genes, the factors between SKN-1 and *elt-2* demonstrate complex patterns of partial or complete redundancy. Some, like *end-1* and *elt-7*, can be individually deleted with no apparent phenotype, whereas other single- and double-mutant combinations result in stochastic expression and many embryos lacking gut (Maduro et al., 2005a; Raj et al., 2010; Sommermann et al., 2010; Ewe et al., 2022). The most-penetrant zygotic defect results from mutants lacking both *end-1* and *end-3* together, which fail to specify gut 100% of the time (Zhu et al., 1997; Maduro et al., 2005a; Owraghi et al., 2010). Generally, any genotype leading to partially compromised specification leads to a loss of robustness of *elt-2* activation and a failure to develop a completely normal intestine in terms of gut differentiation, gut cell number, and metabolic function, showing that the network of factors evolved to make development robust (Maduro et al., 2007, 2015; Raj et al., 2010; Ewe et al., 2022).

The zygotic endoderm genes all encode GATA factors, a family of transcription factors that bind the canonical sequence HGATAR (Lowry and Atchley, 2000; Wiesenfahrt et al., 2015; Du et al., 2016). The MED factors are a divergent subfamily, binding to a related RAGTATAC core sequence (Broitman-Maduro et al., 2005; Lowry et al., 2009). The canonical embryonic *C. elegans* GATA factors, including the endodermal END-1,3 and ELT-2,7 factors, have highly similar DNA-binding domains (DBDs) (Fig. 1C,D) and recognize nearly identical sequences (Weirauch et al., 2014; Wiesenfahrt et al., 2015; Du et al., 2016). In two interesting demonstrations of functional overlap, forced early endoderm expression of ELT-2, using the *end-1* promoter (*end-1p*::ELT-2), can functionally replace the upstream function of *end-3*, *end-1* and *elt-7*; furthermore, the function of all of *end-1,3* and *elt-2,7* can be replaced by a double-transgenic combination of *end-1p*::ELT-7 and *elt-2p*::ELT-7 (Wiesenfahrt et al., 2015; Dineen et al., 2018). The functional overlap does have limits, however, as high copy numbers of these transgenes are required for their function, suggesting there are factor-specific activities that depend on regions upstream of the DBDs (Wiesenfahrt et al., 2015; Dineen et al., 2018).

The recent availability of high-quality genome sequences for dozens of *Caenorhabditis* species has enabled genome-level analysis of evolution of gene families (Félix et al., 2014; Slos et al., 2017; Stevens et al., 2019). In prior work using these sequences, we found no apparent orthologues of the *med*, *end* and *elt-7* genes outside of the Elegans supergroup of species, suggesting these genes evolved over a short time period at its base (Eurmsirilerd and Maduro, 2020; Maduro, 2020) (Fig. 1E). Most nematodes in the broad clade of Rhabditids that includes *Caenorhabditis* have only four ‘core’ embryonic GATA factors that are orthologous to factors found in *C. elegans* (Eurmsirilerd and Maduro, 2020). Aside from ELT-2, there are the ELT-1 and ELT-3 factors, which both function in hypodermal specification, and ELT-5 (EGL-18), which specifies hypodermal cells in the lateral seam (Page et al., 1997; Gilleard and McGhee, 2001; Koh and Rothman, 2001; Koh et al., 2002).

In this work, we examine gut specification outside of the Elegans supergroup using *C. angaria* (Kiontke et al., 2011; Sudhaus et al., 2011). This species has several advantages for study, including its robust growth under laboratory conditions similar to those used for *C. elegans*, and the fact that it has been used in comparative studies by multiple laboratories, with some examples here (Jud et al., 2007; Kuntz et al., 2008; Brauchle et al., 2009; Nuez and Félix, 2012; Barkoulas et al., 2016; Macchietto et al., 2017). RNA interference (RNAi) has shown some success in *C. angaria* (Nuez and Félix, 2012). Embryos of *C. angaria* resemble those of *C. elegans* and undergo a similar development in just over 11 h at 24°C with minor variations in the times at which particular milestones are reached (Macchietto et al., 2017). Using a combination of *C. elegans* transgenics, single-molecule fluorescence *in situ* hybridization (FISH) detection, genetics, and RNAi in *C. angaria*, we present multiple lines of evidence that gut specification in *C. angaria* occurs via Can-POP-1-dependent activation of *Can-elt-3*, and that, in turn, Can-ELT-3 activates *Can-elt-2* to drive gut differentiation. The results suggest an evolutionary origin of the endoderm gene regulatory network in the Elegans supergroup from a simpler GATA factor cascade, representing an example of developmental system drift by both gene duplication and rewiring.

## RESULTS

### An updated high-quality sequence for *C. angaria* PS1010

To facilitate identification of orthologous genes in *C. angaria*, we sequenced and assembled the genome of PS1010 by a combination of Nanopore long reads, Illumina short reads, and Hi-C technology to produce a six-piece chromosome-level assembly (see Materials and Methods). A Hi-C image is shown in Fig. S1. The new sequence represents an improved assembly compared with a previously published draft sequence (Table S1) (Mortazavi et al., 2010).

### Testing requirements for maternal Can-SKN-1 and Can-POP-1

**Fig. 2.**
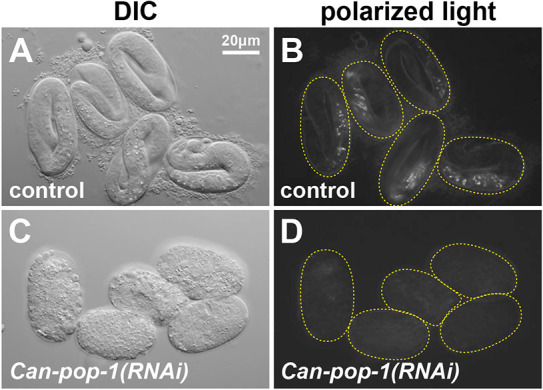
**RNAi of *Can-pop-1*.** (A) RGD1 *C. angaria* control embryos shortly before hatching, visualized by differential interference contrast (DIC). (B) Birefringent gut granules visualized by polarized light; 96% (*n*=123) of control embryos elongated to the threefold stage and 100% contained gut granules. (C) DIC image of onefold arrested embryos of *Can-pop-1(RNAi)*; 226/252 (90%) of progeny embryos lacked differentiated intestine and arrested without significant morphogenesis. (D) Interfered *Can-pop-1(RNAi)* embryos lack gut granules. Dashed lines outline embryos corresponding to those in panels to the left.

To elucidate a pathway for gut specification outside the Elegans supergroup, we began by testing the possibility that the *C. angaria* orthologues of SKN-1 and POP-1 might play a role in gut specification*.* The *Can-skn-1* and *Can-pop-1* orthologues appear to be maternally expressed, as single-embryo RNA-sequencing (RNA-seq) experiments in very early embryos recovered transcripts for these (Macchietto et al., 2017). To deplete function of these genes individually, we used RNAi. Progeny of animals fed control dsRNA displayed normal development and gut granules (Fig. 2A,B; *n*=123). In contrast, we observed a penetrant embryonic lethality with *Can-pop-1(RNAi)*. After >24 h of growth of L4/adult animals on *Can-pop-1* dsRNA-expressing bacteria, 90% of progeny (*n*=252) showed a uniform embryonic arrest at one-fold elongation with several hundred nuclei but no morphogenesis, and an absence of gut granules (Fig. 2C,D). RNAi by injection resulted in the same phenotype, although only 149/234 (64%) of progeny embryos were affected, likely because we injected only a single gonad arm per female to favour survival. The lack of gut in *Can-pop-1(RNAi)* was immediately striking to us, as RNAi of *pop-1* in *C. briggsae* resulted in a similar one-fold gutless phenotype (Lin et al., 2009; Zhao et al., 2010). In those experiments, E adopted the fate of MS, which produces extra pharynx and muscle. We tested for such a transformation by looking for ectopic pharyngeal tissue using single-molecule inexpensive FISH (smiFISH) to detect expression of the *C. angaria* orthologue of the pharyngeal myosin gene *myo-2* (Okkema and Fire, 1994; Tsanov et al., 2016; Parker et al., 2021). However, we did not find evidence of extra *Can-myo-2* expression (0/20 embryos; Fig. S2).

We next attempted RNAi of *Can-skn-1*. Although *Can-pop-1(RNAi)* resulted in a highly penetrant embryonic arrest, *Can-skn-1(RNAi)* resulted in no apparent phenotype (*n*=120 progeny), using both dsRNA injection and RNAi by feeding and with two different targeting sequences. Occasionally, unusual embryos or larvae were observed in less than 5% of progeny that had various morphological or elongation defects; however, these were also observed at a similar frequency following control dsRNA injection, control RNAi by feeding, or no treatment. Because *C. angaria* is a male-female species, these rare embryos likely result from a reduction in developmental robustness due to inbreeding depression (Nuez and Félix, 2012). Regardless, even these rare animals contained differentiated intestine as visualized by gut granules. To control for effectiveness of *Can-skn-1(RNAi)*, we used smiFISH. Expression of the *skn-1* mRNA in *C. elegans* and *C. angaria* was detected throughout four-cell-stage embryos (Fig. S3A,B). We detected *Can-skn-1* mRNA by smiFISH in 97% (*n*=34) of untreated embryos, but in only 6% (*n*=32) of RNAi-treated embryos (Fig. S3C). Hence, *Can-skn-1(RNAi)* treatment was effective at knocking down *Can-skn-1* transcripts. We interpret the lack of *Can-skn-1(RNAi)* phenotype to mean that, unlike in *C. elegans*, the *skn-1* orthologue is dispensable in *C. angaria*.

### Expression of *elt-2* is conserved between *C. elegans* and *C. angaria*

Orthologues of the intermediate endodermal GATA factors from the Elegans supergroup are absent in *C. angaria*. Hence, we next examined *Can-elt-2.* ELT-2 is widely conserved among nematodes (Eurmsirilerd and Maduro, 2020). *Haemonchus contortus*, a parasitic nematode within the Rhabditida order, encodes an apparent *elt-2* orthologue that can promote gut fate when overexpressed in *C. elegans* (Couthier et al., 2004). We therefore predicted that *Can-elt-2* drives intestinal differentiation.

**Fig. 3.**
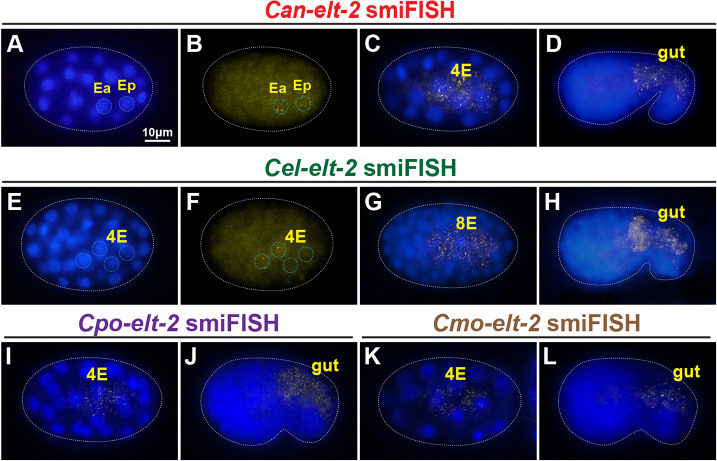
**Expression of *elt-2* orthologues in *C. angaria* and *C. elegans* by smiFISH.** (A,B) Onset of *Can-elt-2* as the 2E cells gastrulate, showing DAPI (A) and smiFISH (B) signals in blue and yellow, respectively. The nuclei of Ea and Ep are outlined. (C,D) Later expression of *Can-elt-2* in the 4E stage and developing gut. (E,F) Onset of *Cel-elt-2* at the 4E stage. The nuclei of the E granddaughters are outlined in blue. (G,H) Later expression of *Cel-elt-2* at 8E stage and the developing gut. (I,J) Expression of *C. portoensis elt-2* at 4E stage and in later gut. (K,L) Expression of *C. monodelphis elt-2* at 4E stage and in later gut. In these and later images, embryos in smiFISH images are outlined by white dotted line, and unless otherwise indicated, anterior is to the left and dorsal is up.

We examined expression of *Can-elt-2* and *Cel-elt-2* using smiFISH (Fig. 3). Consistent with a similar role in intestinal differentiation downstream of specification, we detected *Can-elt-2* transcripts starting at the 2E stage, after the two E daughters had moved into the interior of the embryo, and continuing in the E lineage and intestine at later stages (Fig. 3A-D). This expression is similar to that of *Cel-elt-2* in *C. elegans*, except that *Cel-elt-2* appeared to be activated slightly later, at the 4E stage (Fig. 3E-H). To confirm intestinal *elt-2* expression in other species outside of the Elegans supergroup, we examined *C. portoensis*, a distant relative of *C. angaria*, and *C. monodelphis*, an even more distant species that is considered basal for the genus (Félix et al., 2014; Slos et al., 2017; Stevens et al., 2019). As shown in Fig. 3I-L, the *elt-2* orthologues were expressed in the early E lineage and later gut in both species.

**Fig. 4.**
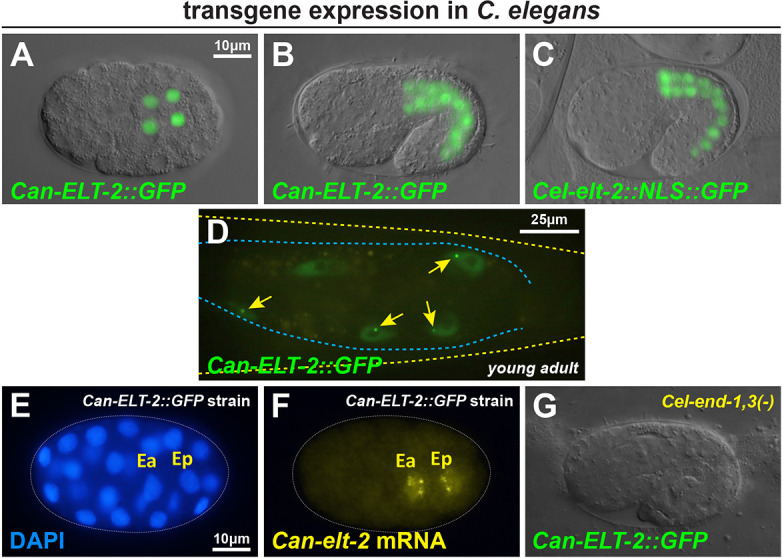
**Expression of *Can-ELT-2::GFP* in *C. elegans* under the control of its own promoter.** (A) *Can-ELT-2::GFP* at the 4E stage. (B) Expression in a 1.5-fold-stage embryo. (C) Expression of a *Cel-elt-2*::NLS::GFP reporter transgene in an embryo slightly younger than that shown in B. The expression was much brighter, so a shorter exposure was used to image the GFP. (D) Subnuclear *Can-ELT-2::GFP* spots (arrows) in intestinal nuclei of young adults. (E,F) smiFISH probes detect nascent *Can*-*ELT-2::GFP* mRNA at the late 2E stage. (G) *Can-ELT-2::GFP* expression fails to occur in an *end-1(ok558) end-3(ok1448)* double-mutant background. In panels A-C and G, a DIC image was overlaid with a fluorescence image.

We next tested whether the entire *Can-elt-2* gene, when introduced into *C. elegans*, is capable of intestinal expression. We expected that activation of *Can-elt-2* in *C. elegans* would occur through endogenous ELT-2 and ELT-7 acting through autoregulatory GATA sites in the *Can-elt-2* promoter (Fukushige et al., 1999; Wiesenfahrt et al., 2015). We amplified the *Can-elt-2* gene with 5.0 kbp of its upstream flanking DNA, the entire coding region including introns, and 231 bp downstream of the stop codon. We inserted the coding region for GFP just before the stop codon. In a wild-type background, the *Can-ELT-2::GFP* transgene was indeed expressed only in intestinal nuclei in *C. elegans*, starting in the early embryo and continuing through adulthood, similar to expression of a *Cel-elt-2* reporter (Fig. 4A-D). We regularly observed a small subnuclear spot of *Can-ELT-2::GFP*, which was particularly prominent in the gut of young adults (Fig. 4D; 36% of 547 gut nuclei examined in 20 worms). These were reminiscent of spots observed from autoregulatory interaction of *C. elegans* ELT-2::GFP protein with the *elt-2* promoter DNA on a multicopy transgene array (Fukushige et al., 1999). The nuclear spots suggest, therefore, that the *C. angaria elt-2* gene is capable of positive autoregulation. A smaller construct with 3.0 kbp of upstream promoter showed identical intestinal expression, and, for the assays described below, we used either transgene interchangeably.

### Expression of Can-ELT-2::GFP in *C. elegans* requires prior gut specification by END-1,3

We used smiFISH to determine more precisely when *Can-ELT-2::GFP* was being activated in *C. elegans*. As shown in Fig. 4E,F, the earliest transcripts of *Can-ELT-2::GFP* were detected in the nuclei of Ea and Ep after the E daughters had ingressed into the embryo, slightly earlier than when *Cel-elt-2* transcripts become detectable, but later than when *Cel-end-3* transcripts first appear (Raj et al., 2010; Nair et al., 2013). This timing suggested that *Can-ELT-2::GFP* was being activated by END-1,3. To test this, we crossed the *Can-ELT-2::GFP* transgene into a double-mutant *end-1(ok558) end-3(ok1448)* strain that is maintained by an *end-3(+)* array marked with *unc-119*::mCherry (Owraghi et al., 2010). We examined embryos in which mCherry was absent, hence are double mutant *end-1 end-3*, but which express *unc-119*::CFP, confirming the presence of the *Can-ELT-2::GFP* array. Of 112 embryos lacking *unc-119::mCherry*, all (100%) lacked *Can-ELT-2::GFP* expression and visible evidence of gut differentiation (Fig. 4G, Table 1). To test whether END-1 by itself was sufficient to activate *Can-ELT-2::GFP*, we introduced the transgene into an *end-3(ok1448)* single mutant. In this background, *Can-ELT-2::GFP* was still expressed in 93% of transgenic animals with gut (*n*=73). We conclude that expression of *Can-ELT-2::GFP* in *C. elegans* requires prior specification of gut by *end-1,3*, an unexpected result because *C. angaria* lacks orthologues of these genes.

**Table 1.**
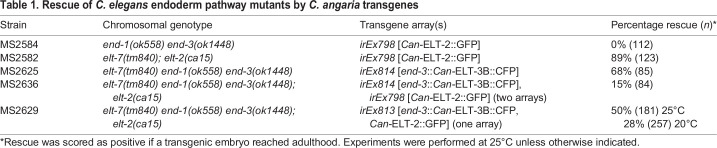
Rescue of *C. elegans* endoderm pathway mutants by *C. angaria* transgenes

### Can-ELT-2::GFP can rescue gut differentiation in *C. elegans*

The early activation of *Can-ELT-2::GFP*, and its possible autoregulation, suggested that *Can-ELT-2::GFP* could substitute for endogenous *Cel-elt-2*. We introduced the *Can-ELT-2::GFP* transgene into a *C. elegans elt-2(ca15); elt-7(tm840)* double-null mutant background, in which animals arrest as first-stage larvae with incompletely developed intestines (Sommermann et al., 2010). As anticipated, *Can-ELT-2::GFP* rescued the larval lethality of the strain to complete viability in 89% (*n*=123) of transgenic animals (Table 1). The ability of *Can-ELT-2::GFP* to rescue a *Cel-elt-2; elt-7* double mutant confirms that *Can-ELT-2::GFP* can drive gut development in *C. elegans* downstream of *Cel-end-1,3*.

### *C. angaria elt-3* is expressed in the early E lineage downstream of Can-POP-1

**Fig. 5.**
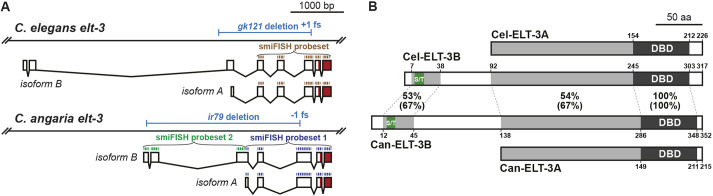
**Gene models and proteins for *C. elegans* and *C. angaria elt-3* orthologues.** (A) Diagrams of the *C. elegans* and *C. angaria elt-3* genes, showing the long and short isoform coding regions. The coding region for the DBD is shaded dark red. Probe sets for smiFISH were designed to be complementary to the regions indicated by the brackets. The *Cel-elt-3(gk121)* and *Can-elt-3(ir79)* deletion alleles are indicated above the genomic DNA. (B) The long and short isoforms of *C. elegans* ELT-3 proteins (Cel-ELT-3B and Cel-ELT-3A, respectively) with the predicted orthologues from *C. angaria*, with results of protein-protein BLAST comparison. Amino acid positions relative to the start of each isoform are indicated. The DBDs are identical, whereas the immediate upstream region common to both short isoforms shows 54% identity (67% similarity). A short 21-23 amino acid region showing 53% identity (67% similarity) is found at the amino end that includes a poly-serine/threonine region (S/T). In *C. elegans*, the poly-S/T region has sequence SSTSSSDS (7/8 amino acids are serine or threonine, 6/8 are serine), and in *C. angaria*, it is SPHSSTDTSS (7/10 amino acids are serine or threonine, 5/10 are serine). Poly-S/T sequences occur in other GATA factors, particularly END-1 and END-3; however, their significance is unknown (Eurmsirilerd and Maduro, 2020; Maduro, 2020).

To explain the activation of *Can-ELT-2::GFP* in *C. elegans* by END-1,3, we speculated that within *C. angaria* endogenous *Can-elt-2* is activated by another GATA factor. We examined *Can-elt-1* and *Can-elt-5* by smiFISH and found that these showed expression similar to their *C. elegans* orthologues with no early E lineage-specific signal (Fig. S4). We next considered *Can-elt-3*, which at first seemed an unlikely candidate*.* From previous work in *C. elegans*, *elt-3* is expressed only in hypodermal cells beginning in mid-embryogenesis (Gilleard et al., 1999). ELT-3 has since been shown to be part of a gene network that drives epidermal specification (Gilleard and McGhee, 2001; Shao et al., 2013). Subsequent studies have found roles for *Cel-elt-3* in oxidative stress responses and regulation of cuticle collagen genes (Budovskaya et al., 2008; Hu et al., 2017; Mesbahi et al., 2020). As shown in Fig. 5, two major isoforms are known for *Cel-elt-3*: a shorter ‘a’ isoform of 226 amino acids (ELT-3A) and a longer ‘b’ isoform of 317 amino acids (ELT-3B) (Li et al., 2020). We predicted a *Can-elt-3* gene model that includes both orthologues, and designed probe sets for smiFISH that would allow detection of both isoforms (probe set 1) or only the longer one (probe set 2).

**Fig. 6.**
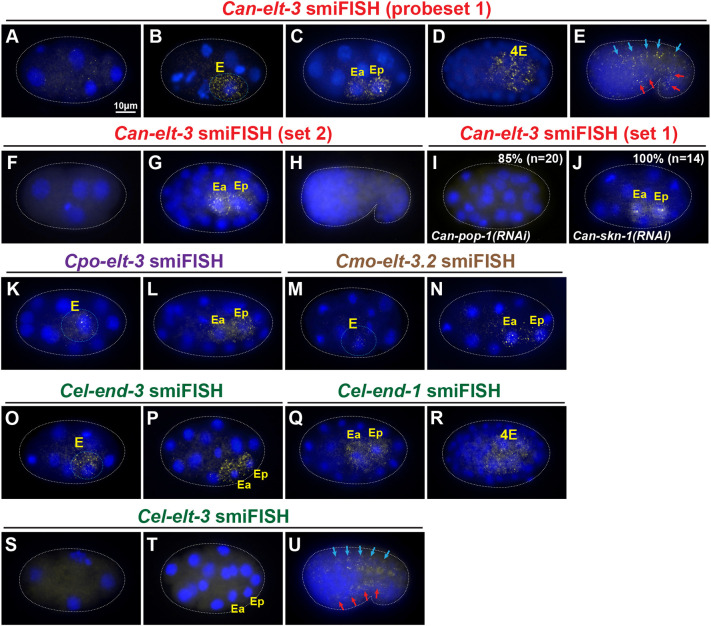
**Expression of *elt-3* orthologues in *C. angaria* and *C. elegans* by smiFISH.** (A-E) *Can-elt-3* transcripts detected by probe set 1, which detects both the long and short isoforms. (A) Low-level maternal transcripts at the four-cell stage. (B-D) Expression in the early E lineage from E through to 4E. (E) Later expression in dorsal (blue arrows) and ventral (red arrows) hypodermal cells. (F-H) *Can-elt-3* transcripts detected by probe set 2, which is specific for the long isoform. (F) Absence of maternal expression. (G) Expression in the early E lineage. (H) Absence of hypodermal expression. (I) Expression of *Can-elt-3* is not detectable in 85% (*n*=20) of *Can-pop-1(RNAi)* embryos. (J) Persistence of *Can-elt-3* expression in *Can-skn-1(RNAi)* embryos (100%, *n*=14). (K,L) Expression of *C. portoensis elt-3* in the early E lineage. (M,N) Expression of *C. monodelphis elt-3.2* in the early E lineage. (O,P) *Cel-end-3* expression from E to 2E. (Q,R) *Cel-end-1* expression from 2E to 4E. (S) Lack of maternal transcripts of *Cel-elt-3* at the four-cell stage. (T) Lack of early E lineage expression of *Cel-elt-3*. (U) Expression of *Cel-elt-3* in dorsal (blue arrows) and ventral (red arrows) hypodermal cells. For clarity, in panels B,K,M,O the E cell has been outlined in a light blue dotted line.

Analysis by smiFISH showed that *Can-elt-3* exhibits both endodermal and hypodermal expression (Fig. 6A-H). Using probe set 1, faint maternal transcripts for *Can-elt-3* were detected in very early embryos (Fig. 6A), appearing much weaker than maternal *Can-skn-1* transcripts (compare with Fig. S3B). Much stronger signal was detectable in the E cell, just after its birth, and the early E descendants up to the 4E stage (Fig. 6B-D), and later in the embryonic hypodermis (Fig. 6E). All of these expression components were previously detected at similar stages by single-embryo RNA-seq (Macchietto et al., 2017). Transcripts were primarily cytoplasmic in most cells; however, we regularly saw one or two bright foci of nuclear staining in E, as well as Ea and Ep, likely representing nascent bursts of transcription of the *Can-elt-3* gene itself (Seydoux and Fire, 1994). *Can-elt-3*, like *Cel-elt-3*, is X-linked; hence, the foci are consistent with nascent transcripts on two X chromosomes in females and one X chromosome in males. When we repeated the staining using probe set 2, we observed only early E lineage expression (Fig. 6F-H), suggesting that the longer *Can-elt-3B* isoform is endoderm specific.

Our earlier observation that *Can-pop-1(RNAi)* results in the loss of gut prompted us to determine whether *Can-pop-1* acts upstream or downstream of *Can-elt-3.* We used smiFISH to detect *Can-elt-3* transcripts in control and *Can-pop-1(RNAi)* embryos. We observed *Can-elt-3* expression in the early E lineage in control embryos from the eight-cell to the ∼50-cell stage (100%, *n*=20), but expression was eliminated in 85% (*n*=20) of similarly staged embryos in *Can-pop-1(RNAi)* (Fig. 6I). The small fraction that did show staining is consistent with our prior measurement of ∼10% of embryos that were unaffected by *Can-pop-1(RNAi).* We also confirmed that knockdown of *Can-skn-1*, which did not exhibit a phenotype, also did not affect *Can-elt-3* expression (14/14 embryos; Fig. 6J). We conclude that *Can-pop-1* is required for *Can-elt-3* expression and therefore acts upstream of gut specification, similar to the *pop-1* orthologues in *C. elegans* and *C. briggsae* (Shetty et al., 2005; Lin et al., 2009; Zhao et al., 2010).

To determine whether early E lineage expression of *elt-3* is likely to be broadly conserved outside the Elegans supergroup, we examined expression in *C. portoensis* and *C. monodelphis.* The former encodes a single *elt-3* orthologue (see Fig. 1E), whereas *C. monodelphis* encodes two (Eurmsirilerd and Maduro, 2020). *Cmo-elt-3.1* showed no embryonic expression (Fig. S5A,B), but we did observe early E lineage expression for *Cpo-elt-3* and for *Cmo-elt-3.2* (Fig. 6K-N). Both also showed later hypodermal expression (Fig. S5C,D). These results are consistent with a widespread role of *elt-3* in gut specification outside of the Elegans supergroup, especially considering the basal placement of *C. monodelphis* in the phylogeny (Slos et al., 2017).

Finally, we examined expression of the *end* genes and the orthologous *elt-3* gene in *C. elegans.* We first examined expression of *Cel-end-3* and *Cel-end-1* to confirm that their overlapping expression patterns (E to 2E, and 2E to 4E, respectively) resemble the expression of *Can-elt-3* in *C. angaria* by smiFISH (E to 4E; Fig. 6O-R). We then examined *Cel-elt-3* to confirm the absence of expression in the early E lineage. We did not detect signal in early embryos (Fig. 6S,T); however, we observed later expression in hypodermal lineages (Fig. 6U), consistent with prior work (Gilleard et al., 1999). Taken together, the data suggest that the endodermal expression of *elt-3* was lost at the base of the Elegans supergroup, but the hypodermal expression has been retained.

### *Can-elt-3* is essential for specification of endoderm

**Fig. 7.**
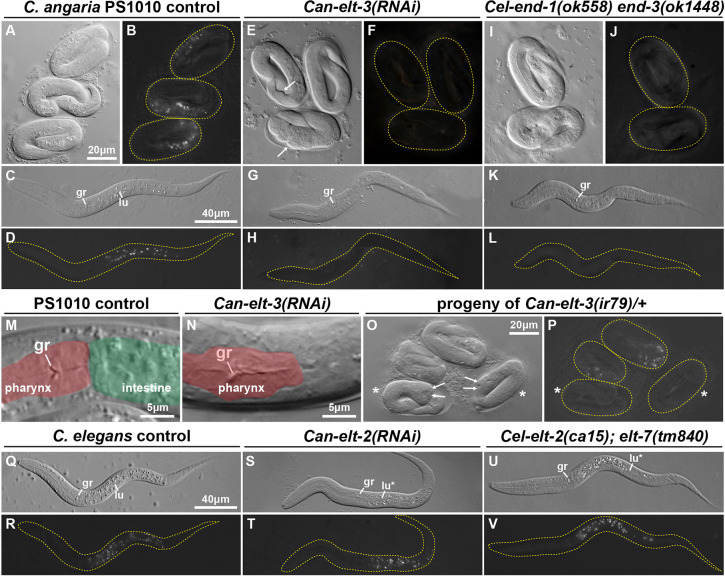
**Gut development phenotypes in *C. angaria* and *C. elegans.*** Most image pairs show DIC (grey images) and polarized light (dark images) to show gut granules. (A,B) Control *C. angaria* PS1010 three-fold embryos. (C,D) Newly hatched *C. angaria* larva. (E,F) Arrested *Can-elt-3(RNAi)* embryos showing absence of gut granules. Arrows indicate hypodermal defects, which were seen in 34% (*n*=29) of gutless embryos. (G,H) Absence of gut in *Can-elt-3(RNAi)* arrested larva. (I,J) Arrested three-fold *Cel-end-1,3(-)* double mutants. (K,L) Absence of gut in arrested *Cel-end-1,3(-)* larva. (M,N) Magnified view of the posterior pharynx region in late-stage embryos. (M) PS1010 embryo showing pharynx (shaded red) joined to intestine (green). (N) Posterior pharynx (red) and absence of intestine in *Can-elt-3(RNAi)* three-fold embryo. (O,P) Twenty-three percent (*n*=140) of progeny from *Can-elt-3(ir79)/+* heterozygous females mated with normal males lacked differentiated gut. Two normal and two apparent *ir79/0* embryos are shown. Gutless embryos are indicated by asterisks and hypodermal defects indicated by arrows. Hypodermal defects were observed in 72% (*n*=25) of gutless animals, but in only 6% (*n*=50) of *ir79/+* or *+/+* controls. (Q,R) Wild-type *C. elegans* newly hatched larva. (S,T) Arrested *Can-elt-2(RNAi)* larva with gut granules and incomplete lumen. (U,V) *C. elegans elt-2(ca15); elt-7(tm840)* arrested larva with abnormal lumen. gr, grinder in pharynx terminal bulb; lu, example of normal gut lumen; lu*, abnormal or patchy gut lumen. In polarized light images, embryos or larvae are outlined with a dashed yellow line.

Because *Can-pop-1(RNAi)* results in a penetrant loss of *Can-elt-3* expression and gut, we hypothesized that *Can-elt-3* specifies gut in *C. angaria.* To test this directly, we performed RNAi by gonadal injection of *Can-elt-3* dsRNA. Whereas control animals always developed intestine (Fig. 7A-D; *n*=102), *Can-elt-3(RNAi)* resulted in arrested embryos and larvae in 76/122 (62%) of progeny in a time window 24-48 h after injection (Fig. 7E-H). We examined these for the presence of birefringent gut granules, ‘fried-egg’ nuclei typical of gut cells, an intestinal lumen, and basement membrane surrounding the intestine. In almost all cases, these features of differentiated gut were completely absent (Fig. 7E-H,M,N). In a small number of embryos, we observed rare gut-like nuclei; however, we could not see a polarized epithelium and gut granule birefringence, and no lumen was visible. Except for these few cases, arrested embryos and larvae were strongly reminiscent of *C. elegans end-1(ok558) end-3(ok1448)* double-null mutants (Fig. 7I-L) (Owraghi et al., 2010). Unlike *Cel-end-1,3(-)* embryos, however, which show variable elongation of two to three times the length, arrested *Can-elt-3(RNAi)* embryos tended to be fully elongated. As well, *Cel-end-1,3(-)* embryos often contain internal hypodermis-lined cavities that result from the transformation of E to a C-like cell when *Cel-end-1* and *Cel-end-3* are absent (Sulston et al., 1983; Zhu et al., 1997; Maduro et al., 2005a). Such cavities were not obvious in *Can-elt-3(RNAi)*, although we did see hypodermal defects, visible as a deformation of part of the cuticle, in 34% (*n*=29) of embryos (Fig. 7E, arrows). These could be the result of loss of *Can-elt-3* in the hypodermis, or from defects in morphogenesis associated with loss of E specification.

We used CRISPR/Cas9 mutagenesis to generate a deletion of *Can-elt-3* in strain PS1010 using a protocol optimized for *C. elegans* (Ghanta and Mello, 2020). We obtained a mutant, *ir79*, that deletes 2916 bp of *Can-elt-3* and is a putative null (shown on the gene model in Fig. 5A). Because *Can-elt-3* is X-linked, the mutant is maintained through heterozygous females; mating with males will produce one out of four hemizygous *ir79* progeny. Of 140 progeny of *Can-elt-3(ir79)/+* females crossed to wild-type males, 32 embryos arrested without gut (23%; *P*=0.6 with expected 25%) and resembled *Can-elt-3(RNAi)* embryos (Fig. 7O,P). These results are consistent with a fully penetrant embryonic lethality of *Can-elt-3(ir79)*, confirming that *Can-elt-3* is zygotically required for gut specification. In addition to the endoderm defect, 72% of gutless animals (*n*=25) showed a hypodermal defect, suggesting that *ir79* mutants have a stronger phenotype than *Can-elt-3(RNAi).* These results also show that *Can-elt-3(RNAi)* phenotypes are not the result of depletion of maternal *Can-elt-3* mRNA.

The essential role of *C. angaria elt-3* contrasts with the absence of developmental phenotype seen in a *C. elegans elt-3* null mutant (Gilleard and McGhee, 2001). To confirm that *Cel-elt-3* plays no minor role in gut specification, we combined the *elt-3(gk121)* null mutant with null mutants in each of *end-1*, *end-3* and *elt-7* to look for possible synergistic effects (Table S2). As expected, we found no evidence of synergy.

### RNAi of *Can-elt-2* results in incomplete gut differentiation

We next confirmed that *Can-elt-2* functions similarly to *Cel-elt-2* by examining *Can-elt-2(RNAi)* using gonadal dsRNA injection. We observed a penetrant larval lethality in 39/89 (44%) of progeny embryos examined 24-72 h after injection. In these arrested larvae, although intestine was present, we observed a variety of differentiation defects, including a partial intestinal lumen and patches of intestine lacking gut granules (Fig. 7S,T). The phenotype was highly reminiscent of the *C. elegans Cel-elt-2(ca15); elt-7(tm840)* double mutant (Fig. 7U,V; compare with Fig. 7Q,R control). We conclude that *Can-elt-2* is required for gut differentiation in *C. angaria*, as expected.

### Overexpression of Can-ELT-3B is sufficient to activate Can-ELT-2::GFP and gut specification in *C. elegans*

**Fig. 8.**
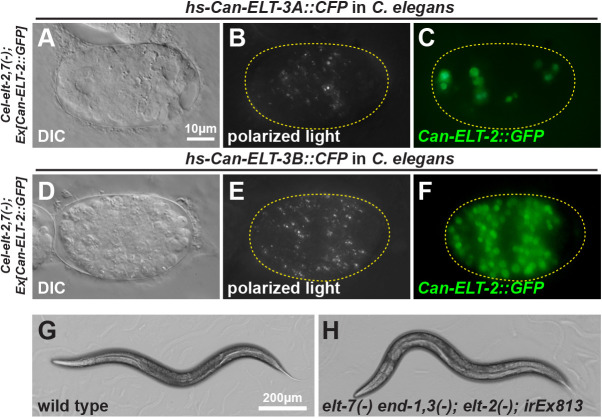
**Gut specification in *C. elegans* by *Can-ELT-3B* and *Can-ELT-2* transgenes.** (A) DIC image of a terminal-stage embryo following *Can-ELT-3A::CFP* heat-shock-induced overexpression. (B) Apparent granule-like material visualized by polarized light. (C) *Can-ELT-2::GFP* expression of the same embryo shown in A,B in <20 nuclei. (D) DIC image of a terminal-stage embryo following *Can-ELT-3B::CFP* overexpression. (E) Widespread distribution of birefringent gut granules. (F) Widespread expression of the *Can-ELT-2::GFP* transgene in >50 nuclei. (G) Phase-contrast image of a control wild-type adult on agar. (H) Adult *elt-7(tm840) end-1(ok558) end-3(ok1448); elt-2(ca15)* quadruple mutant rescued by an extrachromosomal array carrying *Cel-end-3p::Can-ELT-3B::CFP* and *Can-ELT-2::GFP* transgenes.

Prior studies in *C. elegans* showed that endodermal GATA factors are individually able to promote widespread gut specification when overexpressed throughout early embryos (Fukushige et al., 1998; Zhu et al., 1998; Maduro et al., 2001, 2005a; Sommermann et al., 2010). We wished to test whether widespread expression of Can-ELT-3 within *C. elegans* is sufficient to do so. We constructed heat shock (hs) *hs-Can-ELT-3B::CFP* and *hs-Can-ELT-3A::CFP* transgenes to express each isoform conditionally throughout embryos. The transgenes were individually introduced into a *C. elegans elt-2(ca15); elt-7(tm840); Ex[Can-ELT-2::GFP]* strain. We heat-shocked mixed-stage early embryos (<100 cells) for 20 min at 34°C. In both cases, within 75-90 min, widespread nuclear CFP was observed, indicating expression of the transgene [49% (*n*=35) of *hs-Can-ELT-3A::CFP* and 61% (*n*=18) of *hs-Can-ELT-3B::CFP*]. The CFP disappeared by 3 h after heat shock. In the case of *hs-Can-ELT-3A*, most embryos arrested with either no gut or a small patch of gut (*n*=85%, *n*=39), with a small number showing some gut granules and *Can-ELT-2::GFP*-expressing nuclei that were consistent with dispersal of a normal number of gut cells (15%, *n*=39; Fig. 8A-C). In contrast, with *hs-Can-ELT-3B* we observed 37% (*n*=43) of embryos that exhibited one-fold arrest with widespread *Can-ELT-2::GFP* with >50 nuclei (Fig. 8D-F). Parallel treatment of the rescued *elt-2,7* strain carrying *Can-ELT-2::GFP*, without a heat-shock transgene, showed 12% (*n*=50) embryonic arrest but no ectopic gut. These results show that overexpressed Can-ELT-3B, but not Can-ELT-3A, is sufficient to promote gut specification outside of its normal context in *C. elegans*.

We next tested whether expression of Can-ELT-3B in the early E lineage could specify gut in *C. elegans.* We constructed a *Cel-end-3promoter::Can-ELT-3B::CFP* fusion transgene and introduced it, along with an *unc-119::mCherry* marker, into a triple mutant *elt-7(tm840) end-1(ok558) end-3(ok1448)* strain rescued by an *unc-119::YFP-*marked array. We obtained several viable transmitting lines in which the original *unc-119::YFP* array had been replaced by the *end-3::Can-ELT-3B::CFP* array, confirming rescue of specification. The best line rescued 68% (*n*=85) of transgenic animals to complete viability and fertility (Table 1). We next tested whether the combination of *end-3::Can-ELT-3B::CFP* and *Can-ELT-2::GFP* could rescue a strain in which all of *elt-7*, *end-1*, *end-3* and *elt-2* had been mutated. We were able to construct such strains, either using separate arrays containing *end-3::Can-ELT-3B::CFP* and *Can-ELT-2::GFP*, or with a single array containing both transgenes (Fig. 8G,H, Table 1). With two separate arrays, 15% (*n*=84) of double-transgenic embryos were rescued. In the single-array strain, rescue was strongest at 25°C with 50% (*n*=181) of transgenic embryos rescued to full viability, whereas at 20°C rescue dropped to 28% (*n*=257). These striking results demonstrate the ability of the simpler *C. angaria* gut network to replace the core gut specification and differentiation pathway of *C. elegans*.

## DISCUSSION

**Fig. 9.**
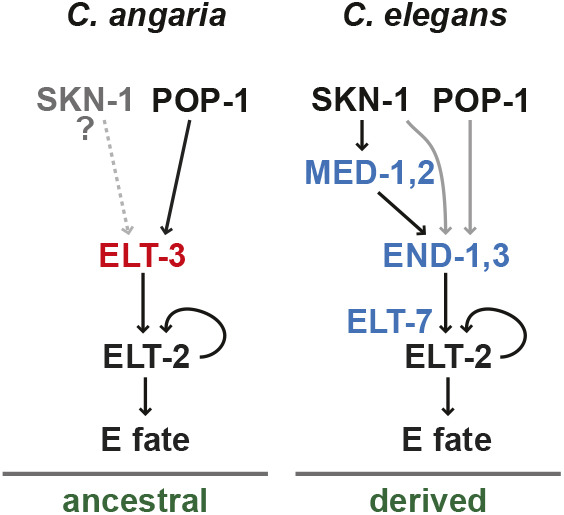
**Simplified core gut network in *C. angaria* compared with *C. elegans***. *In C. angaria*, Can-POP-1, perhaps with another activator, activates zygotic expression of *Can-elt-3*. In turn, Can-ELT-3 activates *Can-elt-2*, which drives gut differentiation and is likely maintained by autoregulation. In the derived *C. elegans* network, endodermal ELT-3 function is absent, and the END-1,3 and ELT-7 factors specify gut downstream of SKN-1/MED-1,2 and POP-1. Black lines indicate strong regulatory interactions, and gray lines indicate weaker ones. The dashed gray line indicates a possible regulatory interaction.

In this work, we have elucidated a core pathway for gut specification and differentiation in a species outside of the Elegans supergroup. This solves a long-standing question about gut specification in *Caenorhabditis*, and adds a new example of a pathway that exhibits developmental system drift (True and Haag, 2001). The simpler pathway consists of a single zygotic specification factor, ELT-3, that serves the function of the three GATA factors END-1, END-3 and ELT-7 that drive endoderm development in *C. elegans* (Fig. 9). Both retain the terminal regulator, ELT-2, which is functionally interchangeable across the evolutionary distance between *C. angaria* and *C. elegans*. Consistent with the essentiality of the network components, loss of *Can-elt-3* by mutation resembles loss of *Cel-end-1,3*, and loss of *Can-elt-2* resembles loss of *Cel-elt-2,7.* When forcibly expressed in *C. elegans*, Can-ELT-3B can activate either endogenous *Cel-elt-2* or transgenic *Can-elt-2* and drive gut development. The simpler network of *C. angaria* is reminiscent of gut development in *Drosophila*, in which two GATA factors act in a similar cascade: *serpent* (*srp*) specifies gut fate upstream of *GATAe*, which executes and maintains this fate (Reuter, 1994; Okumura et al., 2005).

Both pathways in *C. elegans* and *C. angaria* share at least one maternal activator, POP-1/TCF. In *C. elegans*, the positive contribution of POP-1 is secondary to a stronger input by SKN-1, whereas in the close relative *C. briggsae* maternal input from POP-1 and SKN-1 are individually essential (Maduro et al., 2005b; Shetty et al., 2005; Lin et al., 2009; Zhao et al., 2010; Bhambhani et al., 2014). As a result, the phenotype of *pop-1(RNAi)* was similar between *C. angaria* and *C. briggsae*, namely a failure to activate the early E lineage specification factors, *Can-elt-3* in the former, and *Cel-end-1,3* in the latter.

We did not observe a phenotype for *Can-skn-1(RNAi)*, perhaps because it is redundant with another factor, or because Can-SKN-1 does not have a role in endomesoderm specification in *C. angaria* as it does in *C. elegans*. The ancestral role of SKN-1 may not be in embryonic cell specification: SKN-1 is known to be a major effector of postembryonic responses to physiological stress, and, in an unexpected convergence of function, *Cel-elt-3* interacts genetically with *Cel-skn-1* in regulation of genes in the oxidative stress response (Blackwell et al., 2015; Hu et al., 2017). Therefore, it may be that *Can-skn-1* was recruited into endoderm specification at the base of the Elegans supergroup, perhaps concomitantly with the emergence of the MED GATA factors. Further experiments to elucidate contributions of other maternal regulators of cell fate in *C. angaria*, and a more detailed understanding of the fate of the E cell in both *Can-pop-1(RNAi)* and *Can-elt-3(ir79)*, may shed light on how combinatorial mechanisms of cell specification work in *C. angaria* that could explain the lack of a *Can-skn-1(RNAi)* phenotype.

### Differential activity of ELT-3 through long and short isoforms

ELT-3 in *C. elegans* has been associated with hypodermal expression and function, although a null mutation has no developmental phenotype (Gilleard et al., 1999; Gilleard and McGhee, 2001; Shao et al., 2013). In the early E lineage, though not in E itself, a low level of *Cel-elt-3* transcripts has been observed by single-cell transcriptomics (Hashimshony et al., 2012; Tintori et al., 2016). In this study, we failed to observe such expression in intact embryos, and, moreover, found no evidence for even a cryptic role of *Cel-elt-3* in gut specification. Later expression of *Cel-elt-3* in the intestine has been reported, though this has been controversial (Budovskaya et al., 2008; Tonsaker et al., 2012). Potentially, such expression could be conditional, arising in response to oxidative stress as it appears to function in the hypodermis (Budovskaya et al., 2008; Shao et al., 2013; Hu et al., 2017). Our observation of hypodermal expression of *Can-elt-3* in embryos suggests that hypodermal ELT-3 function is conserved in the genus.

Overexpression of ELT-3 throughout *C. elegans* embryos, or in the early E lineage, was previously found to promote widespread hypodermal fates, and not endodermal fates (Fukushige et al., 1998; Gilleard and McGhee, 2001; Wiesenfahrt et al., 2015). This contrasts with our results showing that overexpression of Can-ELT-3 throughout early *C. elegans* embryos, or in the early E lineage, is sufficient to drive gut development. The paradox is resolved by our evidence that a longer isoform, Can-ELT-3B, is endoderm specific, both in its expression in *C. angaria*, and in the ability of this isoform, and not Can-ELT-3A, to activate gut expression when expressed in *C. elegans*. The prior studies in *C. elegans* used only the shorter Cel-ELT-3A isoform (Fukushige et al., 1998; Gilleard and McGhee, 2001). It will be of interest to determine in future studies whether the longer isoform of the *C. elegans* ELT-3 harbours a cryptic ability to activate *Cel-elt-2*, and how the amino-terminal regions found only in the long isoforms, which are conserved between *Can-elt-3* and *Cel-elt-3*, might be important for this activity (Fig. 5B). In human and mouse, protein-protein interactions outside of the DBDs, and combinatorial interactions at promoters, explain differential activities of otherwise similar GATA factors (Romano and Miccio, 2020). Also, a role for vertebrate GATA3 as a pioneer factor was recently described (Tanaka et al., 2020). Hence, it is plausible that the amino-terminal portion of Can-ELT-3B is important for interaction with co-factors, or for a possible role as a pioneer factor in establishing an active transcription state of *Can-elt-2* in the early embryo.

### Expansion of an ancestral network: how and why?

The role of ELT-3 in gut specification is likely to be ancestral. The absence of *med*, *end* and *elt-7* orthologues outside of the Elegans supergroup shows that the *C. elegans* gut network must be derived (Eurmsirilerd and Maduro, 2020; Maduro, 2020). We found that expression of *elt-3* in the early E lineage also occurs in *C. portoensis* and in the basal species *C. monodelphis*, which is further consistent with the ancestral nature of the simpler pathway. The alternative, secondary simplification of the *C. elegans-*type pathway by loss and consolidation of factors, seems far less likely: although some individual genes are dispensable, loss of pairs of regulators in *C. elegans* is inviable or nearly so, and even minor disruption to timely activation of *elt-2* results in abnormal gut development and metabolism (Maduro et al., 2007; Owraghi et al., 2010; Raj et al., 2010; Choi et al., 2017; Ewe et al., 2022). Loss of the upstream MED factors that directly activate *end-1,3* would also result in a failure to specify MS (Maduro et al., 2001). Hence, the *C. elegans-*type expanded network may be evolutionarily fixed.

There are several differences between the *C. angaria-*type network and the derived *C. elegans* one that must have occurred over a very short time span. What originated as a simpler network involving only one upstream GATA factor, ELT-3, must have been rapidly replaced by several regulators, END-1, END-3 and ELT-7. We previously suggested that the *end* and *elt-7* genes might have originated from a duplication of *elt-2*, through a successive cascade of upstream duplication and intercalation into the network through temporal refinement of expression and changes in *cis*-regulation (Maduro, 2020). Our results here suggest that the *end* genes and *elt-7* originated as duplications of *elt-3*. Of the *C. elegans* GATA factors, ELT-3 has features that make it more ‘endodermal’ than hypodermal. For one, it has an intron in the same position as the endodermal GATA factors, between the first two cysteines in the zinc finger (Maduro, 2020). The *elt-1* and *elt-5* genes lack this intron and instead have one farther downstream in the basic domain. Furthermore, the short carboxyl end of ELT-3, which terminates abruptly after the basic domain, is a feature found only among the MED and END factors and ELT-7, as ELT-1, ELT-2 and ELT-5 contain extended regions after the basic domain (Maduro, 2020). As the presumptive paralogs of ELT-3 evolved, the original ELT-3 also had to lose its endoderm specification role, while the upstream MED factors evolved as activators of the ENDs and specifiers of MS fate. The similarity of the MEDs to END-3 suggests that these arose late and were derived from duplication and divergence of an END-3-like factor (Maduro, 2020).

The expansion of an ancestral *elt-3* therefore likely occurred by duplication followed by divergence/specialization. Rapid expansion of gene families is known to have occurred in *C. elegans* (Lipinski et al., 2011; Konrad et al., 2018). The expansion of multiple GLP-1/Notch factor paralogues at the base of the Elegans supergroup is a similar example of expansion of an ancestral factor by gene duplication within the genus (Stevens et al., 2019). In addition, amplification of F-box genes has been observed among four Elegans supergroup species, in which tandem duplication was found to be an important mechanism (Wang et al., 2021). Indeed, among the Elegans supergroup endodermal GATAs, tandem duplication has also been observed for many *med* genes, and there is also the likely ancestral tandem duplication that generated *end-1* and *end-3*, which are found within ∼50 kbp of each other in many species (Maduro, 2020).

Why would a simpler network undergo expansion? Perhaps the expanded network in the Elegans supergroup resulted from evolutionary pressure to accelerate development (Maduro, 2020). Early developmental timing events are slightly accelerated in *C. elegans* relative to *C. angaria*, although later developmental milestones are similarly timed (Macchietto et al., 2017). An increased number of regulators could amplify early specification and assure rapid, robust activation of *elt-2*, permitting development to speed up without sacrificing robustness. The expansion of genes in the Elegans supergroup endoderm network may thus resemble the emergence of *bicoid* in *Drosophila*, in which expansion of a gene network enabled a more rapid embryonic development to occur while maintaining robustness (McGregor, 2005). One intriguing observation from this study may offer an alternative explanation. When we introduced *end-3promoter::Can-ELT-3::CFP* and *Can-ELT-2::GFP* transgenes to rescue gut development in a quadruple *elt-7 end-1 end-3; elt-2* background, rescue was cold sensitive, occurring at lower efficiency at 20°C than at 25°C (Table 1). *C. angaria* is phoretically associated with the weevil *Metamasius hemipterus*, a pest of sugar cane in South Florida and the Caribbean, places with tropical climates (Sudhaus et al., 2011). An intriguing idea is that a more complex network for gut specification enabled *Caenorhabditis* species to maintain robust development as they spread to different locales. With a core gut network now known for the genus, comparative studies within and outside of *Caenorhabditis* can now begin to explore this new system for studying developmental system drift.

## MATERIALS AND METHODS

### Genome sequence of *C. angaria* PS1010

Animals were grown on nematode growth medium (NGM) agar seeded with *Escherichia coli* OP50 for 5 days. Mixed-stage worms were collected from the culture and washed three times with M9 buffer complemented with Anti/Anti (Gibco). The worms were transferred to a worm lysis solution [QIAGEN buffer G2 with 400 µg/ml proteinase K, 50 mM dithiothreitol (QIAGEN) and 0.5 mg/ml RNase A (Invitrogen)] and incubated at 55°C for 4 h. High-molecular-weight genomic DNA was spooled from ethanol precipitation following phenol-chloroform extraction and dissolved in 10 mM Tris (pH 8.0). A Nanopore library was prepared using 1 µg genomic DNA using a ligation sequencing kit (SQK-LSK109, Oxford Nanopore Technologies) according to the manufacturer's protocol. A single 48-h sequencing run was performed with MinION R9.4.1 flow cell to obtain 5.0 Gb of sequence data (444,000 reads; N50, 23 kb). The Nanopore reads were base-called to generate FASTQ files using the Guppy v4.0.15 basecaller (Oxford Nanopore Technologies) with the supplied dna_r9.4.1_450 bps_hac configuration and were quality checked using NanoPlot v1.31.0 (De Coster et al., 2018). An Illumina paired-end sequencing library was prepared from 100 ng of DNA using the Nextera DNA library prep kit according to the manufacturer's instructions. A total of 1.5 Gb of paired-end reads (95 bp×2) were generated by library sequencing on an Illumina MiSeq instrument with the MiSeq reagent kit v3 according to the manufacturer's protocol. The Hi-C library was prepared from ∼2000 fresh worms using an Arima-HiC kit (Arima Genomics) and a Collibri ES DNA library prep kit (Thermo Fisher Scientific) according to the manufacturers' protocols and was sequenced using a MiSeq instrument with the MiSeq reagent kit v3 (101 bp×2), and the 4.9 million short reads were quality checked using the Hi-C quality control pipeline (https://phasegenomics.github.io/2019/09/19/hic-alignment-and-qc.html). The Nanopore long reads were assembled using Nextdenovo v2.4.0 (https://github.com/Nextomics/NextDenovo) with the parameters genome-size=70 M and read_cutoff=5 k. After base correction by three rounds of Pilon v1.23 (Walker et al., 2014) with the Illumina paired-end reads, the assembly was further scaffolded using the 3D-DNA pipeline v180114 (Dudchenko et al., 2017) without a misjoin correction process, and the chromosome-length scaffolds were extracted via manual curation using Juicebox v1.11.08 (Durand et al., 2016). A Hi-C plot is shown in Fig. S1.

### Identification of orthologous genes in *C. angaria*

Orthologous GATA factors and other orthologous genes were identified by BLAST searches as in prior work (Lin et al., 2009; Maduro, 2020). The identity of individual GATA factors was confirmed using defining features, including location of introns in the coding region, signature amino acids within the DBDs, and reciprocal search back to the *C. elegans* genome (Eurmsirilerd and Maduro, 2020). For some genes, we made use of gene predictions from a previously published sequence of *C. angaria* (Mortazavi et al., 2010; Macchietto et al., 2017) and from its close relative *C. castelli* (downloaded from The Caenorhabditis Genomes Project in November, 2020) (Félix et al., 2014). Genome sequences and annotation files for other species were downloaded from The Caenorhabditis Genomes Project and WormBase ParaSite. See Supplementary Materials and Methods for further details.

### *Caenorhabditis* strains and transgenesis

Strains used were: *C. angaria*, PS1010 and RGD1; *C. portoensis*, EG4788; *C. monodelphis*, JU1667. *C. elegans* strains were constructed by standard crosses and microinjections to generate transgene arrays (Brenner, 1974; Mello et al., 1991). Mutations were: *LG III*: *unc-119(ed4)*; *LG IV*: *him-8(e1489)*; *LG V*: *dpy-11(e224)*, *unc-76(e911)*, *elt-7(tm840)*, *end-1(ok558)*, *end-3(ok1448)*; *LG X*: *elt-2(ca15)*, *elt-3(gk121).* Genotypes were confirmed using a combination of progeny testing and PCR with allele-specific primers. Transgene arrays were: *irEx498 [end-3(+) (pMM768), unc-119::mCherry (pMM824)], irEx798 [Can-elt-2_5kbp_promoter::ELT-2genomic::GFP::elt-2_3′UTR (pGB598), unc-119::CFP (pMM809), unc-119(+) (pMM016B)], irEx804 [Cel-end-3promoter::END-3genomic::Can-ELT-3_DNA-binding domain::CFP::Cel-end-3_3′UTR (pGB612), unc-119::YFP (pMM531), unc-119(+) (pMM016B)], irEx808 [hsp16-41::Can-ELT-3(isoform_b)::CFP (pGB619), rol-6D (pRF4)], irEx809 [hsp16-41::Can-ELT-3(isoform_a)::CFP (pGB620), rol-6D (pRF4)], irEx813 [pGB608(Can-elt-2_3.5kbp_promoter::Can-ELT-2::GFP)+pGB618(Cel-end-3::Can-ELT-3B::CFP)+pMM824(unc-119::mCherry)]*, *irEx814 [pGB618(Cel-end-3::Can-ELT-3B::CFP)+pMM824(unc-119::mCherry)]*. Strains used in this work are listed in Table S3.

### Cloning of transgenes

We constructed transgenes using Gibson assembly (Gibson et al., 2009). The coding region for GFP was amplified from pPD95.67 (a gift from Andrew Fire, Stanford University, CA, USA). Plasmids containing coding regions for the fast-folding fluorescent proteins sCFP3A and Venus/YFP (Balleza et al., 2018) were obtained from Addgene (plasmids #103970 and #103986, respectively). Partial or complete coding regions for *Can-elt-3*, *Can-skn-1* and *Can-pop-1* were synthesized by IDT. See Supplementary Materials and Methods for further details.

### Overexpression of Can-ELT-3 by heat shock

We used Gibson assembly to construct intronless heat-shock *Can-ELT-3A::CFP* and heat-shock *Can-ELT-3B::CFP* transgenes using the heat-shock promoter obtained from vector pPD49.83. See Supplementary Materials and Methods for further details. We injected each transgene along with the *rol-6^D^* marker (plasmid pRF4) into the *elt-2(ca15); elt-7(tm840)* genetic background rescued with the *Can-ELT-2::GFP* transgene.

### Rescue of quadruple *elt-7 end-1 end-3; elt-2* mutant

Triple mutant *elt-7(tm840) end-1(ok558) end-3(ok1448)* hermaphrodites rescued by *irEx813* or *irEx814* were crossed to males from a *him-8(e1489); elt-7(tm840) end-1(ok558) end-3(ok1448)* strain rescued by *irEx804*. Progeny males carrying *irEx813* or *irEx814* and lacking *irEx804*, recognized by expression of *unc-119::mCherry* and absence of *unc-119::YFP*, were crossed to *dpy-11(e224) unc-76(e911); elt-2(ca15); irEx798* hermaphrodites. *F*_1_ males carrying *irEx813*, or *irEx814* and *irEx798* (the latter recognizable by *unc-119::CFP*), were backcrossed to *dpy-11 unc-76; elt-2* hermaphrodites. Non-Dpy, non-Unc progeny were allowed to self-fertilize and non-Dpy, non-Unc progeny carrying *irEx813* alone, or *irEx814* together with *irEx798*, were singled to identify animals that never segregated Dpy Unc. These were confirmed by PCR and progeny testing to be quadruple *elt-7(tm840) end-1(ok558) end-3(ok1448); elt-2(ca15)* and rescued by *irEx813* or [*irEx814*+*irEx798*].

### RNAi

For RNAi experiments, genomic DNA fragments or synthesized cDNA sequences were cloned into the feeding-based RNAi vector pPD129.36. For feeding-based RNAi, we used standard protocols (Timmons and Fire, 1998). To synthesize dsRNA for injection, we used primers L4440A (gagcgcagcgagtcagtgagcg) and L4440B (cccagtcacgacgttgtaaaacg) to PCR-amplify a template for synthesis of RNA using the T7 MEGAscript kit (Thermo Fisher Scientific). dsRNA at a concentration of ∼2 μg/μl was injected into one gonad arm per female. To prevent possible cross-interference with mRNA of the other GATA factors, we targeted sequences upstream of the coding regions for the DBDs. See Supplementary Materials and Methods for further details.

### Microscopy and imaging

Images were obtained using either a Canon EOS 77D or Canon EOS RP camera with an LMscope adapter (Micro Tech Labs) on either of two Olympus BX-51 fluorescence microscopes equipped with DIC optics. Images were processed for contrast and colour uniformly across images using Adobe Photoshop.

### CRISPR/Cas9 in *C. angaria*

We used a *C. elegans* protocol (Ghanta and Mello, 2020) with crRNAs to target genomic sequences 5′-gtgcttgaatgcggtgagtttgg-3′ and 5′-gaatttctccaccaactacatgg-3′. All CRISPR reagents were ordered from IDT. We injected 20 females and mated them individually with five males each. One plate had arrested embryos and PCR identified a putative deletion in *Can-elt-3*. We singled 30 mated females and obtained three plates with one out of four dead eggs lacking endoderm. See Supplementary Materials and Methods for further details.

### Detection of RNA *in situ*

We used the smiFISH protocol (Tsanov et al., 2016; Calvo et al., 2021) adapted for use in *Caenorhabditis* by Parker et al. (2021) using our previously described fixation protocol (Broitman-Maduro and Maduro, 2011). Probes consist of complementary FLAP-X sequence (5′-CCTCCTAAGTTTCGAGCTGGACTCAGTG-3′) followed by complementary gene-specific antisense sequence of 16-24 additional bases (Tsanov et al., 2016). These were generated using the Stellaris Probe designer (Biosearch Technologies) and are listed in Supplementary Materials and Methods. Conjugated FLAP-X oligos (CACTGAGTCCAGCTCGAAACTTAGGAGG) that were 5′ and 3′ end-labelled with Quasar 570 or Cal Fluor 610 were synthesized by Biosearch Technologies. FLAP-X oligos 5′ and 3′ end-labelled with Cy5 or Cy3 were synthesized by IDT. To detect fluorescent smiFISH signals, we used filter sets obtained from Chroma: for Quasar 570, we used the Gold FISH 49304 ET set; for Cy5, the Narrow-Excitation Cy5 49009 ET set; for Cal Fluor 610, set 31002 or Red#2 FISH set 49310 ET. We imaged co-stained embryos that had both Quasar 570 and Cy5 probes in order of increasing wavelength, i.e. DAPI→Gold FISH→Cy5, to prevent imaging of photoconverted Cy5 (Cho et al., 2021). In our hands, staining was highly consistent among a set of fixed embryos, such that when signal was detected in embryos of a particular stage, signal was seen in most other embryos of that stage. Rare embryos (<5%) that did not show staining were usually visibly damaged and were more likely to be younger than the four-cell stage. One exception was detection of *Can-elt-2* transcripts in the *Can-ELT-2::GFP* strain, in which ∼60% of embryos showed staining, consistent with the transmission frequency of the extrachromosomal array. We performed smiFISH following RNAi by feeding in *C. angaria* and included controls for permeabilization and staining in each case. For *Can-pop-1(RNAi)*, we simultaneously stained for *Can-elt-3* using Quasar 570, and for *Can-eef1A.1*, the orthologue of *Cel-eef1A.1* (also known as *Cel-eft-3*), using Cy5. From single-embryo RNA-seq data, *Can-eef1A.1* is expressed at all embryonic stages from zygote through hatching (Macchietto et al., 2017). For *Can-skn-1(RNAi)*, we stained for *Can-skn-1* using Quasar 570 and *Can-elt-3* using Cy5. Because *Can-skn-1(RNAi)* did not result in a loss of gut specification, we reasoned that *Can-elt-3* expression would be unaffected and hence this served both as confirmation of this hypothesis as well as a control for staining of *Can-skn-1* transcripts following *Can-skn-1(RNAi)*. For each probe set, we examined 30-100 embryos. See Supplementary Materials and Methods for further details.

## Supplementary Material

10.1242/develop.200984_sup1 Supplementary information

## References

[DEV200984C1] Balleza, E., Kim, J. M. and Cluzel, P. (2018). Systematic characterization of maturation time of fluorescent proteins in living cells. *Nat. Methods* 15, 47-51. 10.1038/nmeth.4509 29320486 PMC5765880

[DEV200984C2] Barkoulas, M., Vargas Velazquez, A. M., Peluffo, A. E. and Félix, M. A. (2016). Evolution of new cis-regulatory motifs required for cell-specific gene expression in caenorhabditis. *PLoS Genet.* 12, e1006278. 10.1371/journal.pgen.1006278 27588814 PMC5010242

[DEV200984C3] Bhambhani, C., Ravindranath, A. J., Mentink, R. A., Chang, M. V., Betist, M. C., Yang, Y. X., Koushika, S. P., Korswagen, H. C. and Cadigan, K. M. (2014). Distinct DNA binding sites contribute to the TCF transcriptional switch in C. elegans and Drosophila. *PLoS Genet.* 10, e1004133. 10.1371/journal.pgen.1004133 24516405 PMC3916239

[DEV200984C4] Blackwell, T. K., Steinbaugh, M. J., Hourihan, J. M., Ewald, C. Y. and Isik, M. (2015). SKN-1/Nrf, stress responses, and aging in Caenorhabditis elegans. *Free Radic. Biol. Med.* 88 (Pt B), 290-301. 10.1016/j.freeradbiomed.2015.06.008 26232625 PMC4809198

[DEV200984C5] Bowerman, B., Eaton, B. A. and Priess, J. R. (1992). skn-1, a maternally expressed gene required to specify the fate of ventral blastomeres in the early C. elegans embryo. *Cell* 68, 1061-1075. 10.1016/0092-8674(92)90078-Q 1547503

[DEV200984C6] Brauchle, M., Kiontke, K., MacMenamin, P., Fitch, D. H. and Piano, F. (2009). Evolution of early embryogenesis in rhabditid nematodes. *Dev. Biol.* 335, 253-262. 10.1016/j.ydbio.2009.07.033 19643102 PMC2763944

[DEV200984C7] Brenner, S. (1974). The genetics of Caenorhabditis elegans. *Genetics* 77, 71-94. 10.1093/genetics/77.1.71 4366476 PMC1213120

[DEV200984C8] Broitman-Maduro, G. and Maduro, M. F. (2011). In situ hybridization of embryos with antisense RNA probes. *Methods Cell Biol.* 106, 253-270. 10.1016/B978-0-12-544172-8.00009-8 22118280

[DEV200984C9] Broitman-Maduro, G., Maduro, M. F. and Rothman, J. H. (2005). The noncanonical binding site of the MED-1 GATA factor defines differentially regulated target genes in the C. elegans mesendoderm. *Dev. Cell* 8, 427-433. 10.1016/j.devcel.2005.01.014 15737937

[DEV200984C10] Budovskaya, Y. V., Wu, K., Southworth, L. K., Jiang, M., Tedesco, P., Johnson, T. E. and Kim, S. K. (2008). An elt-3/elt-5/elt-6 GATA transcription circuit guides aging in C. elegans. *Cell* 134, 291-303. 10.1016/j.cell.2008.05.044 18662544 PMC4719053

[DEV200984C11] Calvo, L., Ronshaugen, M. and Pettini, T. (2021). smiFISH and embryo segmentation for single-cell multi-gene RNA quantification in arthropods. *Commun. Biol.* 4, 352. 10.1038/s42003-021-01803-0 33742105 PMC7979837

[DEV200984C12] Cho, Y., An, H. J., Kim, T., Lee, C. and Lee, N. K. (2021). Mechanism of Cyanine5 to Cyanine3 photoconversion and its application for high-density single-particle tracking in a living cell. *J. Am. Chem. Soc.* 143, 14125-14135. 10.1021/jacs.1c04178 34432445

[DEV200984C13] Choi, H., Broitman-Maduro, G. and Maduro, M. F. (2017). Partially compromised specification causes stochastic effects on gut development in C. elegans. *Dev. Biol.* 427, 49-60. 10.1016/j.ydbio.2017.05.007 28502614

[DEV200984C14] Couthier, A., Smith, J., McGarr, P., Craig, B. and Gilleard, J. S. (2004). Ectopic expression of a Haemonchus contortus GATA transcription factor in Caenorhabditis elegans reveals conserved function in spite of extensive sequence divergence. *Mol. Biochem. Parasitol.* 133, 241-253. 10.1016/j.molbiopara.2003.10.012 14698436

[DEV200984C15] Davidson, E. H. and Levine, M. S. (2008). Properties of developmental gene regulatory networks. *Proc. Natl. Acad. Sci. USA* 105, 20063-20066. 10.1073/pnas.0806007105 19104053 PMC2629280

[DEV200984C16] De Coster, W., D'Hert, S., Schultz, D. T., Cruts, M. and Van Broeckhoven, C. (2018). NanoPack: visualizing and processing long-read sequencing data. *Bioinformatics* 34, 2666-2669. 10.1093/bioinformatics/bty149 29547981 PMC6061794

[DEV200984C17] Dineen, A., Osborne Nishimura, E., Goszczynski, B., Rothman, J. H. and McGhee, J. D. (2018). Quantitating transcription factor redundancy: the relative roles of the ELT-2 and ELT-7 GATA factors in the C. elegans endoderm. *Dev. Biol.* 435, 150-161. 10.1016/j.ydbio.2017.12.023 29360433 PMC6476323

[DEV200984C18] Du, L., Tracy, S. and Rifkin, S. A. (2016). Mutagenesis of GATA motifs controlling the endoderm regulator elt-2 reveals distinct dominant and secondary cis-regulatory elements. *Dev. Biol.* 412, 160-170. 10.1016/j.ydbio.2016.02.013 26896592 PMC4814310

[DEV200984C19] Dudchenko, O., Batra, S. S., Omer, A. D., Nyquist, S. K., Hoeger, M., Durand, N. C., Shamim, M. S., Machol, I., Lander, E. S., Aiden, A. P. et al. (2017). De novo assembly of the Aedes aegypti genome using Hi-C yields chromosome-length scaffolds. *Science* 356, 92-95. 10.1126/science.aal3327 28336562 PMC5635820

[DEV200984C20] Durand, N. C., Robinson, J. T., Shamim, M. S., Machol, I., Mesirov, J. P., Lander, E. S. and Aiden, E. L. (2016). Juicebox provides a visualization system for Hi-C contact maps with unlimited zoom. *Cell Syst.* 3, 99-101. 10.1016/j.cels.2015.07.012 27467250 PMC5596920

[DEV200984C21] Eurmsirilerd, E. and Maduro, M. F. (2020). Evolution of developmental GATA factors in nematodes. *J. Dev. Biol.* 8, 27. 10.3390/jdb8040027 33207804 PMC7712238

[DEV200984C22] Ewe, C. K., Sommermann, E. M., Kenchel, J., Flowers, S. E., Maduro, M. F., Joshi, P. M. and Rothman, J. H. (2022). Feedforward regulatory logic controls the specification-to-differentiation transition and terminal cell fate during Caenorhabditis elegans endoderm development. *Development* 149, 12. 10.1242/dev.200337 PMC10656426 35758255

[DEV200984C23] Félix, M. A., Braendle, C. and Cutter, A. D. (2014). A streamlined system for species diagnosis in Caenorhabditis (Nematoda: Rhabditidae) with name designations for 15 distinct biological species. *PLoS One* 9, e94723. 10.1371/journal.pone.0094723 24727800 PMC3984244

[DEV200984C24] Fukushige, T., Hawkins, M. G. and McGhee, J. D. (1998). The GATA-factor elt-2 is essential for formation of the Caenorhabditis elegans intestine. *Dev. Biol.* 198, 286-302. 9659934

[DEV200984C25] Fukushige, T., Hendzel, M. J., Bazett-Jones, D. P. and McGhee, J. D. (1999). Direct visualization of the elt-2 gut-specific GATA factor binding to a target promoter inside the living Caenorhabditis elegans embryo. *Proc. Natl. Acad. Sci. USA* 96, 11883-11888. 10.1073/pnas.96.21.11883 10518545 PMC18381

[DEV200984C26] Fukushige, T., Goszczynski, B., Tian, H. and McGhee, J. D. (2003). The evolutionary duplication and probable demise of an endodermal GATA factor in Caenorhabditis elegans. *Genetics* 165, 575-588. 10.1093/genetics/165.2.575 14573471 PMC1462794

[DEV200984C27] Ghanta, K. S. and Mello, C. C. (2020). Melting dsDNA donor molecules greatly improves precision genome editing in Caenorhabditis elegans. *Genetics* 216, 643-650. 10.1534/genetics.120.303564 32963112 PMC7648581

[DEV200984C28] Gibson, D. G., Young, L., Chuang, R. Y., Venter, J. C., Hutchison, C. A., 3rd and Smith, H. O. (2009). Enzymatic assembly of DNA molecules up to several hundred kilobases. *Nat. Methods* 6, 343-345. 10.1038/nmeth.1318 19363495

[DEV200984C29] Gilleard, J. S. and McGhee, J. D. (2001). Activation of hypodermal differentiation in the Caenorhabditis elegans embryo by GATA transcription factors ELT-1 and ELT-3. *Mol. Cell. Biol.* 21, 2533-2544. 10.1128/MCB.21.7.2533-2544.2001 11259601 PMC86885

[DEV200984C30] Gilleard, J. S., Shafi, Y., Barry, J. D. and McGhee, J. D. (1999). ELT-3: a Caenorhabditis elegans GATA factor expressed in the embryonic epidermis during morphogenesis. *Dev. Biol.* 208, 265-280. 10.1006/dbio.1999.9202 10191044

[DEV200984C31] Hashimshony, T., Wagner, F., Sher, N. and Yanai, I. (2012). CEL-seq: single-cell RNA-Seq by multiplexed linear amplification. *Cell Rep.* 2, 666-673. 10.1016/j.celrep.2012.08.003 22939981

[DEV200984C32] Hinman, V. F., Nguyen, A. and Davidson, E. H. (2007). Caught in the evolutionary act: precise cis-regulatory basis of difference in the organization of gene networks of sea stars and sea urchins. *Dev. Biol.* 312, 584-595. 10.1016/j.ydbio.2007.09.006 17956756

[DEV200984C33] Hu, Q., D'Amora, D. R., MacNeil, L. T., Walhout, A. J. M. and Kubiseski, T. J. (2017). The oxidative stress response in Caenorhabditis elegans requires the GATA transcription factor ELT-3 and SKN-1/Nrf2. *Genetics* 206, 1909-1922. 10.1534/genetics.116.198788 28600327 PMC5560797

[DEV200984C34] Jud, M., Razelun, J., Bickel, J., Czerwinski, M. and Schisa, J. A. (2007). Conservation of large foci formation in arrested oocytes of Caenorhabditis nematodes. *Dev. Genes Evol.* 217, 221-226. 10.1007/s00427-006-0130-3 17216268

[DEV200984C35] Kiontke, K. C., F, élix, M. A., Ailion, M., Rockman, M. V., Braendle, C., Pénigault, J. B. and Fitch, D. H. (2011). A phylogeny and molecular barcodes for Caenorhabditis, with numerous new species from rotting fruits. *BMC Evol. Biol.* 11, 339. 10.1186/1471-2148-11-339 22103856 PMC3277298

[DEV200984C36] Koh, K. and Rothman, J. H. (2001). ELT-5 and ELT-6 are required continuously to regulate epidermal seam cell differentiation and cell fusion in C. elegans. *Development* 128, 2867-2880. 10.1242/dev.128.15.2867 11532911

[DEV200984C37] Koh, K., Peyrot, S. M., Wood, C. G., Wagmaister, J. A., Maduro, M. F., Eisenmann, D. M. and Rothman, J. H. (2002). Cell fates and fusion in the C. elegans vulval primordium are regulated by the EGL-18 and ELT-6 GATA factors – apparent direct targets of the LIN-39 Hox protein. *Development* 129, 5171-5180. 10.1242/dev.129.22.5171 12399309

[DEV200984C38] Konrad, A., Flibotte, S., Taylor, J., Waterston, R. H., Moerman, D. G., Bergthorsson, U. and Katju, V. (2018). Mutational and transcriptional landscape of spontaneous gene duplications and deletions in Caenorhabditis elegans. *Proc. Natl. Acad. Sci. USA* 115, 7386-7391. 10.1073/pnas.1801930115 29941601 PMC6048555

[DEV200984C39] Kuntz, S. G., Schwarz, E. M., DeModena, J. A., De Buysscher, T., Trout, D., Shizuya, H., Sternberg, P. W. and Wold, B. J. (2008). Multigenome DNA sequence conservation identifies Hox cis-regulatory elements. *Genome Res.* 18, 1955-1968. 10.1101/gr.085472.108 18981268 PMC2593573

[DEV200984C40] Li, R., Ren, X., Ding, Q., Bi, Y., Xie, D. and Zhao, Z. (2020). Direct full-length RNA sequencing reveals unexpected transcriptome complexity during Caenorhabditis elegans development. *Genome Res.* 30, 287-298. 10.1101/gr.251512.119 32024662 PMC7050527

[DEV200984C41] Lin, R., Thompson, S. and Priess, J. R. (1995). pop-1 encodes an HMG box protein required for the specification of a mesoderm precursor in early C. elegans embryos. *Cell* 83, 599-609. 10.1016/0092-8674(95)90100-0 7585963

[DEV200984C42] Lin, K. T., Broitman-Maduro, G., Hung, W. W., Cervantes, S. and Maduro, M. F. (2009). Knockdown of SKN-1 and the Wnt effector TCF/POP-1 reveals differences in endomesoderm specification in C. briggsae as compared with C. elegans. *Dev. Biol.* 325, 296-306. 10.1016/j.ydbio.2008.10.001 18977344 PMC2648516

[DEV200984C43] Lipinski, K. J., Farslow, J. C., Fitzpatrick, K. A., Lynch, M., Katju, V. and Bergthorsson, U. (2011). High spontaneous rate of gene duplication in Caenorhabditis elegans. *Curr. Biol.* 21, 306-310. 10.1016/j.cub.2011.01.026 21295484 PMC3056611

[DEV200984C44] Lowry, J. A. and Atchley, W. R. (2000). Molecular evolution of the GATA family of transcription factors: conservation within the DNA-binding domain. *J. Mol. Evol.* 50, 103-115. 10.1007/s002399910012 10684344

[DEV200984C45] Lowry, J. A., Gamsjaeger, R., Thong, S. Y., Hung, W., Kwan, A. H., Broitman-Maduro, G., Matthews, J. M., Maduro, M. and Mackay, J. P. (2009). Structural analysis of MED-1 reveals unexpected diversity in the mechanism of DNA recognition by GATA-type zinc finger domains. *J. Biol. Chem.* 284, 5827-5835. 10.1074/jbc.M808712200 19095651

[DEV200984C46] Macchietto, M., Angdembey, D., Heidarpour, N., Serra, L., Rodriguez, B., El-Ali, N. and Mortazavi, A. (2017). Comparative transcriptomics of steinernema and Caenorhabditis single embryos reveals orthologous gene expression convergence during late embryogenesis. *Genome Biol. Evol.* 9, 2681-2696. 10.1093/gbe/evx195 29048526 PMC5714130

[DEV200984C47] Maduro, M. F. (2017). Gut development in C. elegans. *Semin. Cell Dev. Biol.* 66, 3-11. 10.1016/j.semcdb.2017.01.001 28065852

[DEV200984C48] Maduro, M. F. (2020). Evolutionary dynamics of the SKN-1→MED→END-1,3 regulatory gene cascade in Caenorhabditis endoderm specification. *G3* 10, 333-356. 10.1534/g3.119.400724 31740453 PMC6945043

[DEV200984C49] Maduro, M. F., Meneghini, M. D., Bowerman, B., Broitman-Maduro, G. and Rothman, J. H. (2001). Restriction of mesendoderm to a single blastomere by the combined action of SKN-1 and a GSK-3beta homolog is mediated by MED-1 and −2 in C. elegans. *Mol. Cell* 7, 475-485. 10.1016/S1097-2765(01)00195-2 11463373

[DEV200984C50] Maduro, M. F., Lin, R. and Rothman, J. H. (2002). Dynamics of a developmental switch: recursive intracellular and intranuclear redistribution of Caenorhabditis elegans POP-1 parallels Wnt-inhibited transcriptional repression. *Dev. Biol.* 248, 128-142. 10.1006/dbio.2002.0721 12142026

[DEV200984C51] Maduro, M., Hill, R. J., Heid, P. J., Newman-Smith, E. D., Zhu, J., Priess, J. and Rothman, J. (2005a). Genetic redundancy in endoderm specification within the genus Caenorhabditis. *Dev. Biol.* 284, 509-522. 10.1016/j.ydbio.2005.05.016 15979606

[DEV200984C52] Maduro, M. F., Kasmir, J. J., Zhu, J. and Rothman, J. H. (2005b). The Wnt effector POP-1 and the PAL-1/Caudal homeoprotein collaborate with SKN-1 to activate C. elegans endoderm development. *Dev. Biol.* 285, 510-523. 10.1016/j.ydbio.2005.06.022 16084508

[DEV200984C53] Maduro, M. F., Broitman-Maduro, G., Mengarelli, I. and Rothman, J. H. (2007). Maternal deployment of the embryonic SKN-1-->MED-1,2 cell specification pathway in C. elegans. *Dev. Biol.* 301, 590-601. 10.1016/j.ydbio.2006.08.029 16979152

[DEV200984C54] Maduro, M. F., Broitman-Maduro, G., Choi, H., Carranza, F., Chia-Yi Wu, A. and Rifkin, S. A. (2015). MED GATA factors promote robust development of the C. elegans endoderm. *Dev. Biol.* 404, 66-79. 10.1016/j.ydbio.2015.04.025 25959238 PMC4469534

[DEV200984C55] McGregor, A. P. (2005). How to get ahead: the origin, evolution and function of bicoid. *BioEssays* 27, 904-913. 10.1002/bies.20285 16108065

[DEV200984C56] Mello, C. C., Kramer, J. M., Stinchcomb, D. and Ambros, V. (1991). Efficient gene transfer in C.elegans: extrachromosomal maintenance and integration of transforming sequences. *EMBO J.* 10, 3959-3970. 10.1002/j.1460-2075.1991.tb04966.x 1935914 PMC453137

[DEV200984C57] Mesbahi, H., Pho, K. B., Tench, A. J., Leon Guerrero, V. L. and MacNeil, L. T. (2020). Cuticle collagen expression is regulated in response to environmental stimuli by the GATA transcription factor ELT-3 in Caenorhabditis elegans. *Genetics* 215, 483-495. 10.1534/genetics.120.303125 32229533 PMC7268988

[DEV200984C58] Mortazavi, A., Schwarz, E. M., Williams, B., Schaeffer, L., Antoshechkin, I., Wold, B. J. and Sternberg, P. W. (2010). Scaffolding a Caenorhabditis nematode genome with RNA-seq. *Genome Res.* 20, 1740-1747. 10.1101/gr.111021.110 20980554 PMC2990000

[DEV200984C59] Nair, G., Walton, T., Murray, J. I. and Raj, A. (2013). Gene transcription is coordinated with, but not dependent on, cell divisions during C. elegans embryonic fate specification. *Development* 140, 3385-3394. 10.1242/dev.098012 23863485 PMC3737719

[DEV200984C60] Nuez, I. and F, élix, M. A. (2012). Evolution of susceptibility to ingested double-stranded RNAs in Caenorhabditis nematodes. *PLoS One* 7, e29811. 10.1371/journal.pone.0029811 22253787 PMC3256175

[DEV200984C61] Okkema, P. G. and Fire, A. (1994). The Caenorhabditis elegans NK-2 class homeoprotein CEH-22 is involved in combinatorial activation of gene expression in pharyngeal muscle. *Development* 120, 2175-2186. 10.1242/dev.120.8.2175 7925019

[DEV200984C62] Okumura, T., Matsumoto, A., Tanimura, T. and Murakami, R. (2005). An endoderm-specific GATA factor gene, dGATAe, is required for the terminal differentiation of the Drosophila endoderm. *Dev. Biol.* 278, 576-586. 10.1016/j.ydbio.2004.11.021 15680371

[DEV200984C63] Owraghi, M., Broitman-Maduro, G., Luu, T., Roberson, H. and Maduro, M. F. (2010). Roles of the Wnt effector POP-1/TCF in the C. elegans endomesoderm specification gene network. *Dev. Biol.* 340, 209-221. 10.1016/j.ydbio.2009.09.042 19818340 PMC2854320

[DEV200984C64] Page, B. D., Zhang, W., Steward, K., Blumenthal, T. and Priess, J. R. (1997). ELT-1, a GATA-like transcription factor, is required for epidermal cell fates in Caenorhabditis elegans embryos. *Genes Dev.* 11, 1651-1661. 10.1101/gad.11.13.1651 9224715

[DEV200984C65] Parker, D. M., Winkenbach, L. P., Parker, A., Boyson, S. and Nishimura, E. O. (2021). Improved methods for single-molecule fluorescence in situ hybridization and immunofluorescence in Caenorhabditis elegans embryos. *Curr. Protoc.* 1, e299. 10.1002/cpz1.299 34826343 PMC9020185

[DEV200984C66] Raj, A., Rifkin, S. A., Andersen, E. and van Oudenaarden, A. (2010). Variability in gene expression underlies incomplete penetrance. *Nature* 463, 913-918. 10.1038/nature08781 20164922 PMC2836165

[DEV200984C67] Reuter, R. (1994). The gene serpent has homeotic properties and specifies endoderm versus ectoderm within the Drosophila gut. *Development* 120, 1123-1135. 10.1242/dev.120.5.1123 7913013

[DEV200984C68] Rocheleau, C. E., Downs, W. D., Lin, R., Wittmann, C., Bei, Y., Cha, Y. H., Ali, M., Priess, J. R. and Mello, C. C. (1997). Wnt signaling and an APC-related gene specify endoderm in early C. elegans embryos. *Cell* 90, 707-716. 10.1016/S0092-8674(00)80531-0 9288750

[DEV200984C69] Romano, O. and Miccio, A. (2020). GATA factor transcriptional activity: insights from genome-wide binding profiles. *IUBMB Life* 72, 10-26. 10.1002/iub.2169 31574210

[DEV200984C70] Seydoux, G. and Fire, A. (1994). Soma-germline asymmetry in the distributions of embryonic RNAs in Caenorhabditis elegans. *Development* 120, 2823-2834. 10.1242/dev.120.10.2823 7607073

[DEV200984C71] Shao, J., He, K., Wang, H., Ho, W. S., Ren, X., An, X., Wong, M. K., Yan, B., Xie, D., Stamatoyannopoulos, J. et al. (2013). Collaborative regulation of development but independent control of metabolism by two epidermis-specific transcription factors in Caenorhabditis elegans. *J. Biol. Chem.* 288, 33411-33426. 10.1074/jbc.M113.487975 24097988 PMC3829187

[DEV200984C72] Shetty, P., Lo, M. C., Robertson, S. M. and Lin, R. (2005). C. elegans TCF protein, POP-1, converts from repressor to activator as a result of Wnt-induced lowering of nuclear levels. *Dev. Biol.* 285, 584-592. 10.1016/j.ydbio.2005.07.008 16112103

[DEV200984C73] Shukla, V., Habib, F., Kulkarni, A. and Ratnaparkhi, G. S. (2014). Gene duplication, lineage-specific expansion, and subfunctionalization in the MADF-BESS family patterns the Drosophila wing hinge. *Genetics* 196, 481-496. 10.1534/genetics.113.160531 24336749 PMC3914621

[DEV200984C74] Slos, D., Sudhaus, W., Stevens, L., Bert, W. and Blaxter, M. (2017). Caenorhabditis monodelphis sp. n.: defining the stem morphology and genomics of the genus Caenorhabditis. *BMC Zool.* 2, 15. 10.1186/s40850-017-0013-2

[DEV200984C75] Sommermann, E. M., Strohmaier, K. R., Maduro, M. F. and Rothman, J. H. (2010). Endoderm development in Caenorhabditis elegans: the synergistic action of ELT-2 and -7 mediates the specification→differentiation transition. *Dev. Biol.* 347, 154-166. 10.1016/j.ydbio.2010.08.020 20807527 PMC3142750

[DEV200984C76] Stauber, M., Jäckle, H. and Schmidt-Ott, U. (1999). The anterior determinant bicoid of Drosophila is a derived Hox class 3 gene. *Proc. Natl. Acad. Sci. USA* 96, 3786-3789. 10.1073/pnas.96.7.3786 10097115 PMC22372

[DEV200984C77] Stauber, M., Prell, A. and Schmidt-Ott, U. (2002). A single Hox3 gene with composite bicoid and zerknullt expression characteristics in non-Cyclorrhaphan flies. *Proc. Natl. Acad. Sci. USA* 99, 274-279. 10.1073/pnas.012292899 11773616 PMC117551

[DEV200984C78] Stevens, L., élix, F., M. A., Beltran, T., Braendle, C., Caurcel, C., Fausett, S., Fitch, D., Frézal, L., Gosse, C. et al. (2019). Comparative genomics of 10 new Caenorhabditis species. *Evol. Lett.* 3, 217-236. 10.1002/evl3.110 31007946 PMC6457397

[DEV200984C79] Stevens, L., Rooke, S., Falzon, L. C., Machuka, E. M., Momanyi, K., Murungi, M. K., Njoroge, S. M., Odinga, C. O., Ogendo, A., Ogola, J. et al. (2020). The genome of Caenorhabditis bovis. *Curr. Biol.* 30, 1023-1031.e4. 10.1016/j.cub.2020.01.074 32109387

[DEV200984C80] Sudhaus, W., Giblin-Davis, R. and Kiontke, K. (2011). Description of Caenorhabditis angaria n. sp. (Nematoda: Rhabditidae), an associate of sugarcane and palm weevils (Coleoptera: Curculionidae). *Nematology* 13, 61-78. 10.1163/138855410X500334

[DEV200984C81] Sulston, J. E., Schierenberg, E., White, J. G. and Thomson, J. N. (1983). The embryonic cell lineage of the nematode Caenorhabditis elegans. *Dev. Biol.* 100, 64-119. 10.1016/0012-1606(83)90201-4 6684600

[DEV200984C82] Tanaka, H., Takizawa, Y., Takaku, M., Kato, D., Kumagawa, Y., Grimm, S. A., Wade, P. A. and Kurumizaka, H. (2020). Interaction of the pioneer transcription factor GATA3 with nucleosomes. *Nat. Commun.* 11, 4136. 10.1038/s41467-020-17959-y 32811816 PMC7434886

[DEV200984C83] Thorpe, C. J., Schlesinger, A., Carter, J. C. and Bowerman, B. (1997). Wnt signaling polarizes an early C. elegans blastomere to distinguish endoderm from mesoderm. *Cell* 90, 695-705. 10.1016/S0092-8674(00)80530-9 9288749

[DEV200984C84] Timmons, L. and Fire, A. (1998). Specific interference by ingested dsRNA. *Nature* 395, 854. 10.1038/27579 9804418

[DEV200984C85] Tintori, S. C., Osborne Nishimura, E., Golden, P., Lieb, J. D. and Goldstein, B. (2016). A transcriptional lineage of the early C. elegans Embryo. *Dev. Cell* 38, 430-444. 10.1016/j.devcel.2016.07.025 27554860 PMC4999266

[DEV200984C86] Tonsaker, T., Pratt, R. M. and McGhee, J. D. (2012). Re-evaluating the role of ELT-3 in a GATA transcription factor circuit proposed to guide aging in C. elegans. *Mech. Ageing Dev.* 133, 50-53. 10.1016/j.mad.2011.09.006 22001047

[DEV200984C87] True, J. R. and Haag, E. S. (2001). Developmental system drift and flexibility in evolutionary trajectories. *Evol. Dev.* 3, 109-119. 10.1046/j.1525-142x.2001.003002109.x 11341673

[DEV200984C88] Tsanov, N., Samacoits, A., Chouaib, R., Traboulsi, A. M., Gostan, T., Weber, C., Zimmer, C., Zibara, K., Walter, T., Peter, M. et al. (2016). smiFISH and FISH-quant - a flexible single RNA detection approach with super-resolution capability. *Nucleic Acids Res.* 44, e165. 10.1093/nar/gkw784 27599845 PMC5159540

[DEV200984C89] Walker, B. J., Abeel, T., Shea, T., Priest, M., Abouelliel, A., Sakthikumar, S., Cuomo, C. A., Zeng, Q., Wortman, J., Young, S. K. et al. (2014). Pilon: an integrated tool for comprehensive microbial variant detection and genome assembly improvement. *PLoS One* 9, e112963. 10.1371/journal.pone.0112963 25409509 PMC4237348

[DEV200984C90] Wang, A., Chen, W. and Tao, S. (2021). Genome-wide characterization, evolution, structure, and expression analysis of the F-box genes in Caenorhabditis. *BMC Genomics* 22, 889. 10.1186/s12864-021-08189-7 34895149 PMC8665587

[DEV200984C91] Weirauch, M. T., Yang, A., Albu, M., Cote, A. G., Montenegro-Montero, A., Drewe, P., Najafabadi, H. S., Lambert, S. A., Mann, I., Cook, K. et al. (2014). Determination and inference of eukaryotic transcription factor sequence specificity. *Cell* 158, 1431-1443. 10.1016/j.cell.2014.08.009 25215497 PMC4163041

[DEV200984C92] Wiesenfahrt, T., Berg, J. Y., Nishimura, E. O., Robinson, A. G., Goszczynski, B., Lieb, J. D. and McGhee, J. D. (2015). The function and regulation of the GATA factor ELT-2 in the C. elegans Endoderm. *Development* 143, 483-491. 10.1242/dev.130914 26700680 PMC4760314

[DEV200984C93] Zhao, Z., Flibotte, S., Murray, J. I., Blick, D., Boyle, T. J., Gupta, B., Moerman, D. G. and Waterston, R. H. (2010). New tools for investigating the comparative biology of Caenorhabditis briggsae and C. elegans. *Genetics* 184, 853-863. 10.1534/genetics.109.110270 20008572 PMC2845351

[DEV200984C94] Zhu, J., Hill, R. J., Heid, P. J., Fukuyama, M., Sugimoto, A., Priess, J. R. and Rothman, J. H. (1997). end-1 encodes an apparent GATA factor that specifies the endoderm precursor in Caenorhabditis elegans embryos. *Genes Dev.* 11, 2883-2896. 10.1101/gad.11.21.2883 9353257 PMC316658

[DEV200984C95] Zhu, J., Fukushige, T., McGhee, J. D. and Rothman, J. H. (1998). Reprogramming of early embryonic blastomeres into endodermal progenitors by a Caenorhabditis elegans GATA factor. *Genes Dev.* 12, 3809-3814. 10.1101/gad.12.24.3809 9869634 PMC317268

