# Supplementary material for: The GATA factor ELT-3 specifies endoderm in Caenorhabditis angaria in an ancestral gene network
Source: Development. 2022 Oct 24;149(21):dev200984. doi: 10.1242/dev.200984 (PMC9720673; doi:10.1242/dev.200984)
Supplement: Supplementary information [file develop-149-200984-s1.pdf]

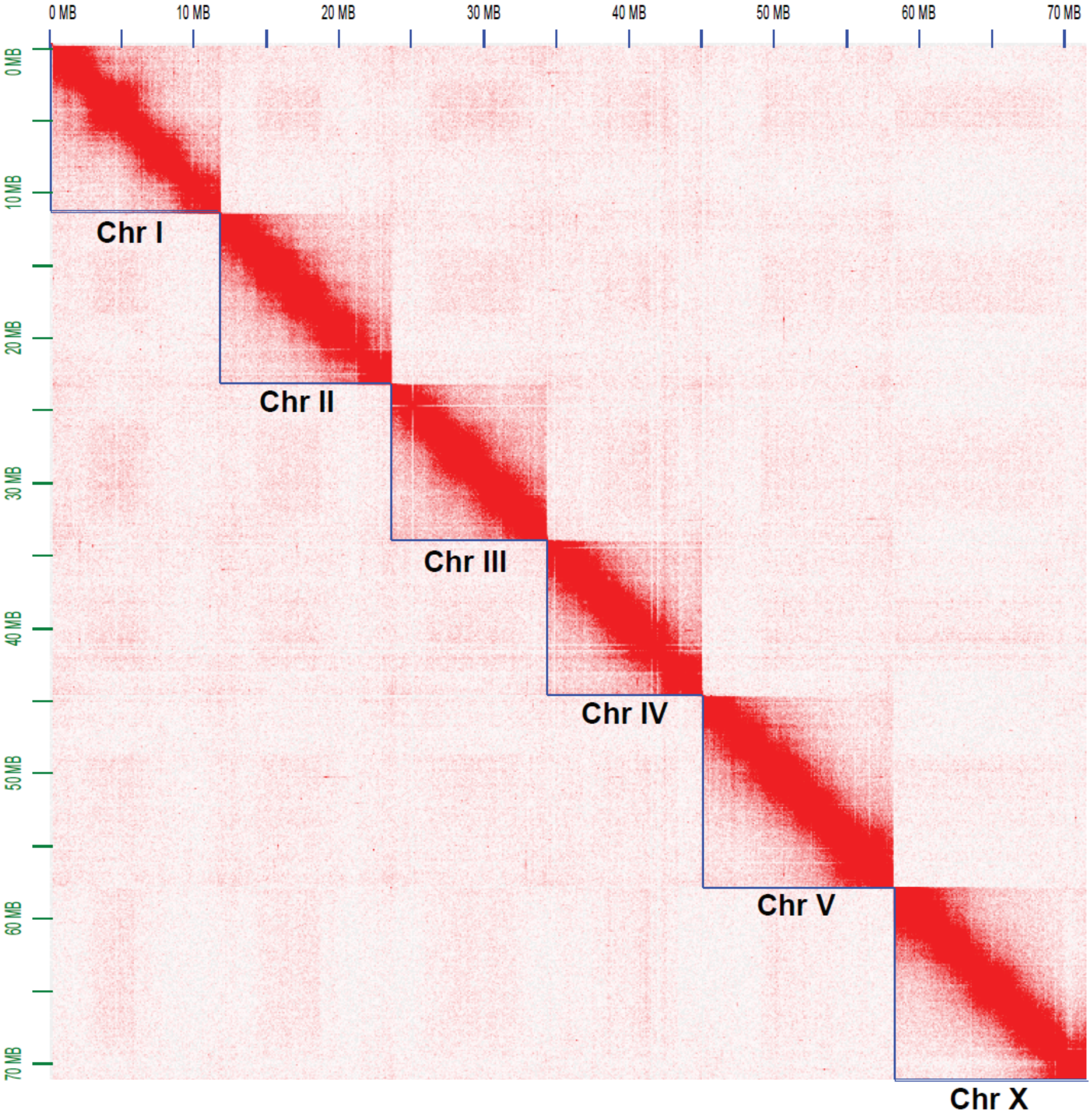

Fig. S1. Hi-C map for *C. angaria* PS1010 sequence indicating linkage groups.

Development • Supplementary information

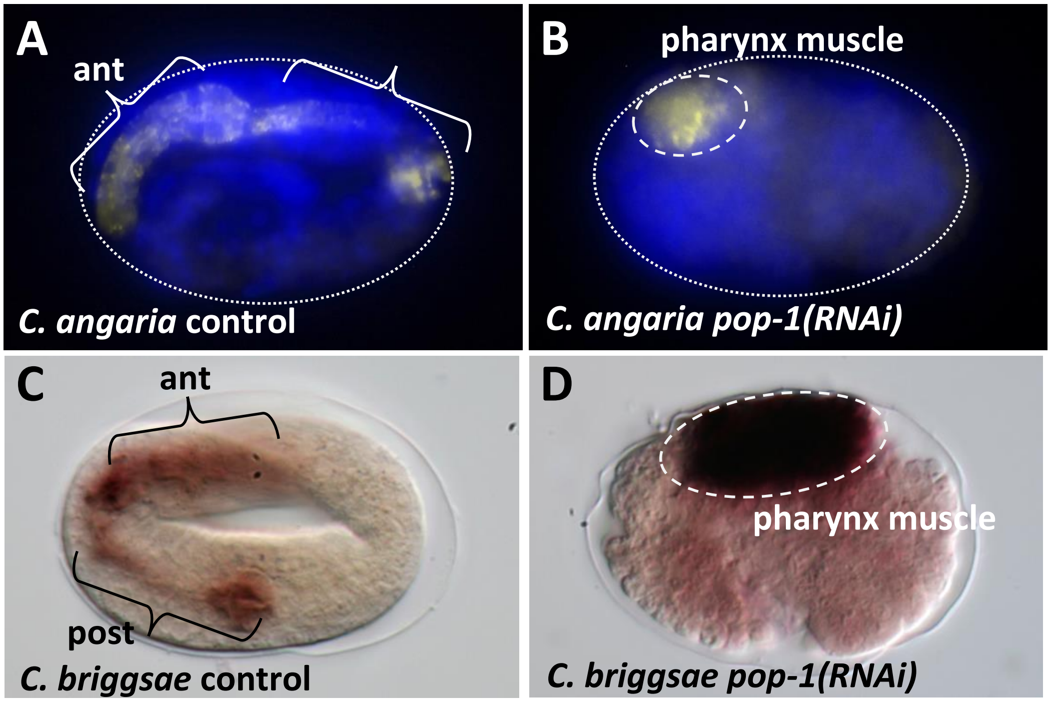

Fig. S2. smiFISH and *in situ* hybridization to detect *myo-2.* (A) Representative PS1010 control 3x stage embryo stained with DAPI (blue) and *Can-myo-2* smiFISH probes (yellow). Staining detects the anterior and posterior pharynx (ant ph and post ph, respectively). 10/10 embryos showed similar staining. (B) Representative arrested *Can-pop-1(RNAi)* embryo stained similarly as in (A). Ectopic *Can-myo-2* expression was not apparent in 20/20 embryos. Because *Can-pop-1(RNAi)* embryos do not elongate, proper morphogenesis of the pharynx does not occur. (C,D) Images from a prior work where ectopic *Cbri-myo-2* tissue was detected in *Cbri-pop-1(RNAi)* using an older *in situ* hybridization protocol (Lin et al., 2009). (D) Extra *Cbri-myo-2* expression is seen because of an E to MS transformation, resulting in extra pharynx muscle. Embryos are approximately 50 μm long.

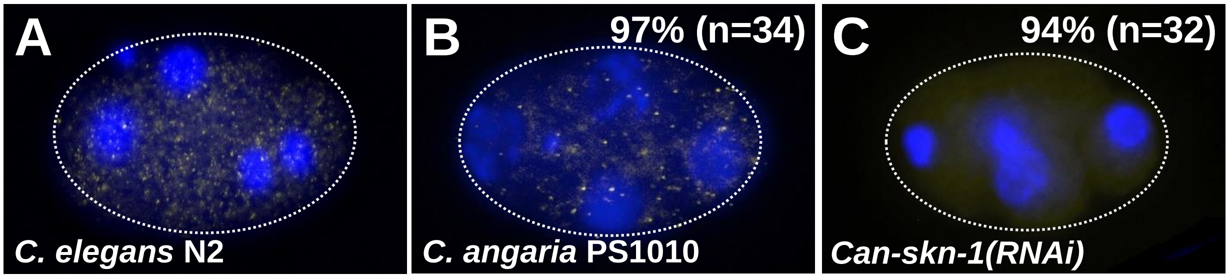

Fig. S3. Expression of *Cel-skn-1* and control for effectiveness of *Can-skn-1(RNAi).* (A) Expression of *C. elegans skn-1* by smiFISH. (B) *Can-skn-1* mRNA was detected in 97% (n=34) of controls. (C) *Can-skn-1* mRNA was absent in 94% (n=32) of *Can-skn-1(RNAi)* embryos.

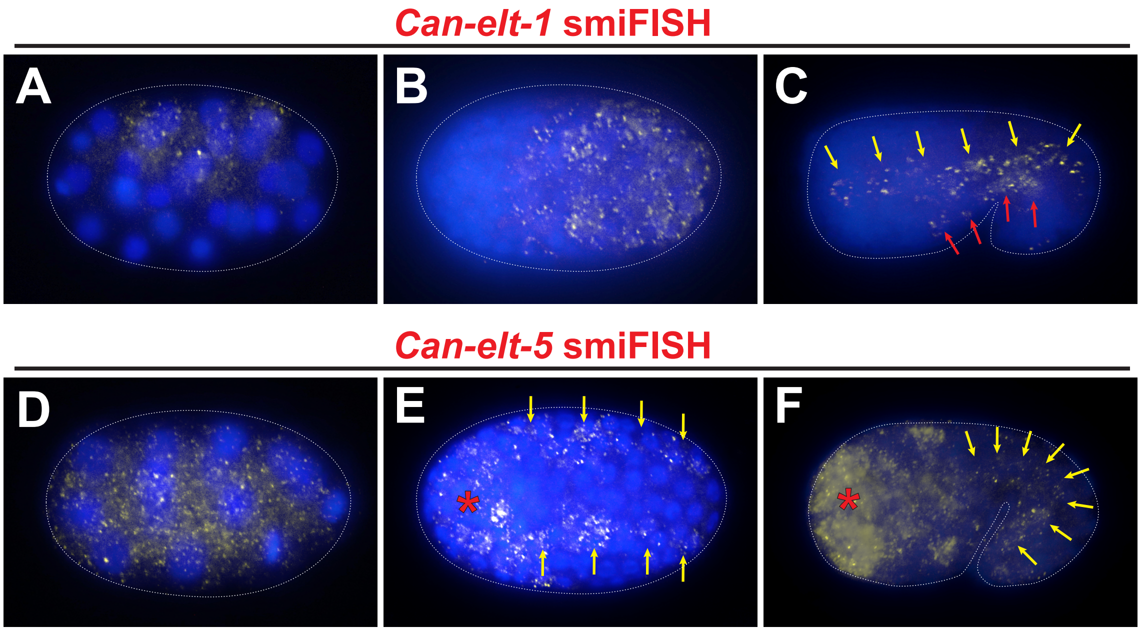

Fig. S4. Expression of *Can-elt-1* (A-C) and *Can-elt-5* (D-F) by smiFISH. (A) Expres-sion of *Can-elt-1* was seen in dorsal hypodermal precursors at gastrulation stage. (B) Dorsal aspect showing widespread expression in hypodermal precursors at late one-fold stage. (C) Expression in lateral seam (yellow arrows) and ventral hypodermis (red arrows) at 1.3-fold stage. (D) Widespread expression of *Can-elt-5* in a 16-cell stage embryo. (E) Dorsal aspect showing expression in anterior cells (*) and lateral hypoder-mal cells (yellow arrows) of late one-fold stage. (F) Anterior expression (*) and lateral hypodermal expression (yellow arrows) in 1.5-fold stage. Expression of *Can-elt-1* and *Can-elt-5* is similar to their orthologous genes in *C. elegans* (Page et al., 1997; Koh and Rothman, 2001). Anterior is to the left and dorsal is up in (A), (C), (D), (F).

Development • Supplementary information

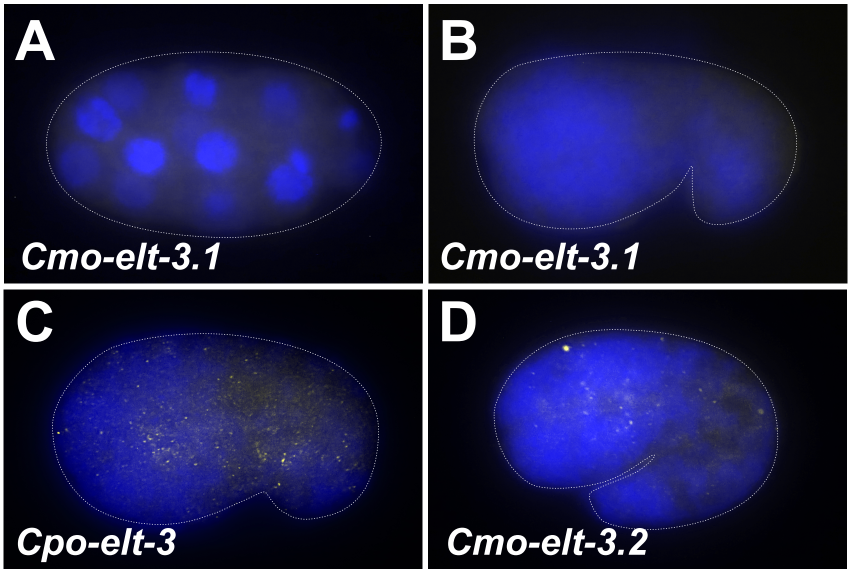

Fig. S5. Absence of embryonic expression of *Cmo-elt-3.1*, and hypodermal expression of *Cpo-elt-3* and *Cmo-elt-3.2*. (A) No expression of *Cmo-elt-3.1* was seen in early embryos (2E stage shown here). (B) No hypodermal expression was seen in later stages (comma stage shown here). (C) Expression of *Cpo-elt-3* in hypodermis of a bean stage embryo. (D) Expression of *Cmo-elt-3.2* in 1.7x stage embryo consistent with hypodermal cells.

Table S1. ***C. angaria*** **genome sequence statistics**

	C. angaria (this study)	***C. angaria*** (Wormbase; PRJNA51225.WS285)
Assembly size (Mb)	71.4	106.0
Number of scaffolds	6	34621
Average (kb)	11,895	3
Largest scaffold (kb)	13,323	868
Gaps (kbp)	6	11,442
GC (%)	33.7	35.7

Table S2. Tests of synergy of elt-3(gk121) with mutants in the C. elegans gut gene network Percentage of embryos containing gut and average number of gut nuclei made. An integrated *elt-2::GFP* reporter transgene, *wIs84*, was used to mark both the presence of gut as well as to count the number of gut nuclei at the end of embryogenesis (Owraghi et al., 2010). In pairwise combinations, none of the comparisons of mutant; *elt-3(+)* with mutant; *elt-3(gk121)* in gut nucleus number is significant (p>0.05 for all, *t*-test). Single *elt-7* null mutants make gut 100% of the time (McGhee et al., 2007). We did not test *elt-7(tm840); wIs84* alone. However, a comparison of *elt-7; elt-3* with *elt-3* alone was not significant (p>0.8).

Strain	Genotype	% making gut (n)	# of gut nuclei ± SD (n)
JR1130	wild type	100% (40)	19.7 ± 0.8 (40)
MS2046	*elt-3(gk121)*	100% (40)	20.0 ± 0.5 (40)
JR2719	*end-1(ok558)*	100% (40)	19.9 ± 0.6 (40)
MS2048	*end-1(ok558); elt-3(gk121)*	100% (130)	19.8 ± 0.6 (30)
MS2041	*end-3(ok1448)*	95.5% (201)	17.8 ± 5.3 (192)
MS2042	*end-3(ok1448); elt-3(gk121)*	95.0% (161)	18.1 ± 5.6 (153)
MS2049	*elt-7(tm840); elt-3(gk121)*	100% (104)	20.0 ± 0.6 (30)

Development • Supplementary information

Table S3. Nematode strains used in this work

C. elegans strains	
Strain Designation	Description
JR1130	*wIs84 [elt-2::NLS::GFP::lacZ, rol-6D] X*
JR2719	*end-1(ok558) V; wIs84 [elt-2::NLS::GFP::lacZ, rol-6D] X*
MS2041	*end-3(ok1448) V; wIs84 [elt-2::NLS::GFP::lacZ, rol-6D] X*
MS2042	*end-3(ok1448) V; elt-3(gk121) wIs84 [elt-2::NLS::GFP::lacZ, rol-6D] X*
MS2046	*elt-3(gk121) wIs84 [elt-2::NLS::GFP::lacZ, rol-6D] X*
MS2048	*end-1(ok558) V; elt-3(gk121) wIs84 [elt-2::NLS::GFP::lacZ, rol-6D] X*
MS2049	*elt-7(tm840) V; elt-3(gk121) wIs84 [elt-2::NLS::GFP::lacZ, rol-6D] X*
MS2582	*elt-7(tm840) V; elt-2(ca15) X; irEx798 [pGB598(Can-ELT-2::GFP), pMM809(unc-119::CFP), pDP#MM016B(unc-119(+))]*
MS2583	*unc-119(ed4); end-3(ok1448) V; irEx798 [pGB598(Can-elt-2_5kbp_promoter::ELT-2::GFP) + pMM809(unc-119::CFP) + pDP#MM016B(unc-119(+))]*
MS2584	*unc-119(ed4) III; end-1(ok558) end-3(ok1448) V; irEx498 [pMM768(end-3(+)), pMM824(unc-119::mCherry)]; irEx798 [pGB598(Can-elt-2_5kbp_promoter::ELT-2::GFP) + pMM809(unc-119::CFP) + pDP#MM016B(unc-119(+))]*
MS2606	*unc-119(ed4) III; him-8(e1489) IV; elt-7(tm840) end-1(ok558) end-3(ok1448) V; irEx804 [pGB612(end-3::END-3upstream::CanELT-3DBD::CFP) + pDP#MM016B(unc-119(+)) + pMM531(unc-119::YFP)]*
MS2612	*elt-2(ca15) X; elt-7(tm840) III; irEx803 [pGB608(Can-elt-2_3kbp_promoter::ELT-2::GFP), pDP#MM016B(unc-119(+))]; irEx808 [pGB619(hsp16-41::CanELT-3B::CFP) + rol-6D]*
MS2617	*elt-2(ca15) X; elt-7(tm840) III; irEx803 [pGB608(Can-elt-2_3kbp_promoter::ELT-2::GFP), pDP#MM016B(unc-119(+))]; irEx809 [pGB620(hsp16-41::CanELT-3A::CFP) + rol-6D]*
MS2625	*elt-7(tm840) end-1(ok558) end-3(ok1448) V; irEx814 [pGB618(Cel-end-3::Can-ELT-3B::CFP) + pMM824(unc-119::mCherry)]*
MS2629	*elt-7(tm840) end-1(ok558) end-3(ok1448) V; elt-2(ca15) X; irEx813 [pGB608(Can-elt-2_3kbp_promoter::Can-ELT-2::GFP) + pGB618(Cel-end-3::Can-ELT-3B::CFP) + pMM824(unc-119::mCherry)]*
MS2636	*elt-7(tm840) end-1(ok558) end-3(ok1448) V; irEx798 [pGB598(Can-elt-2_5kbp_promoter::ELT-2::GFP) + pMM809(unc-119::CFP) + pMM016B]; irEx814 [pGB618(Cel-end-3::Can-ELT-3B::CFP) + pMM824(unc-119::mCherry)]*
N2	*C. elegans* reference strain
Other species	
Strain Designation	Description
EG4788	*C. portoensis* reference strain
JU1667	*C. monodelphis* reference strain
MS2628	*C. angaria elt-3(ir79)/+ X*
PS1010	*C. angaria* reference strain
RGD1	*C. angaria* wild isolate

Development • Supplementary information

References

Koh, K. and Rothman, J. H. (2001). ELT-5 and ELT-6 are required continuously to regulate epidermal seam cell differentiation and cell fusion in C. elegans. *Development* **128**, 2867-2880.

McGhee, J.D., Sleumer, M.C., Bilenky, M., Wong, K., McKay, S.J., Goszczynski, B., Tian, H., Krich, N.D., Khattra, J., Holt, R.A., et al. (2007). The ELT-2 GATA-factor and the global regulation of transcription in the C. elegans intestine. *Dev. Biol.* **302**, 627-645.

Owraghi, M., Broitman-Maduro, G., Luu, T., Roberson, H., Maduro, M.F. (2010). Roles of the Wnt effector POP-1/TCF in the C. elegans endomesoderm specification gene network. *Dev. Biol.* **340**, 209-221.

Page, B. D., Zhang, W., Steward, K., Blumenthal, T. and Priess, J. R. (1997). ELT-1, a GATA-like transcription factor, is required for epidermal cell fates in Caenorhabditis elegans embryos. *Genes Dev.* **11**, 1651-1661.

Development • Supplementary information

Supplementary Materials and Methods:

1) gene models - sequences and coding DNA sequences (CDSs)

2) RNAi clone information

3) plasmid maps and cloning information

4) smiFISH probesets

5) CRISPR/Cas9 PCR primers and sequence of *ir79* allele

1) GENE MODELS:

Can-eft-3 (eef1A.1)

join(2248..2391,2447..>3436,3494..3760)

gatgtttggaatagtgcggaaacggtgatgacgtagagaaaacttgtagtcttacgatagcattacatttttgcggcacaggaattgggc
taatccggaaaaaaattaaatagaatttcaaagatggttctatccagtcacgtaccatcggactatttaggattcatatgaaaaaatgac
ctttctgaatcagcctagatgttcgagttccaatcatgaaatttccagaccttggaaatttatgaggttcagaagttatattctcaatgaaattt
tgcaatcccatttttgtagtggttgttctttgaacttttgttaggaaaaaagtattctccaagtctgcactaaatttcaaaatgcaattttccaga
tggtcagattctacggcgcaatgtttcgtgaaggagatgtgtggatttgtatggaagtaatggacacgtcactcgacaaattctatcgaca
agcatacaaattaggcaaaaagataccagaagcgtttatcagcaaaatgtcgttgtcagtcatcgaaggattgaatttcatgaaagaa
caattgaatttgattcatcgcgatgtgaaaccatcgaatattttattgaataggcatggtcaagtgaaaatttgcgattttggtatctctggac
atttgacaaatagtttagcaaaggtgcgttatacgagagaattttcattaaaaaaaagaaacgatttcagacagttcaagcgggttgtaa
gccttatatgccaccagagcgaattgatggcgagacaaaaactgcatatgatgttcgagctgatgtatggagcttgggaataactatca
ttgaaattgctactggaacacacccctacgcaaaatggaaaacaccatttgatcaattgaaacaagtggttcgagagccccctccaa
aacttccaatgaatgaaggtttcagtgaagaatgccaattatttgttcaaagatggtgggtagtttgttttgctcattaaaattaattgaactg
ggatttcactcaaaatgaaaacgtcactcaaaaaacagaaattaaaaatagaacttacttgcagcctcgaaaaggattataacaatc
gaccaaaatatcccgaacttcttgcgatgaaatttattgaagcggcgagaaatgacaagcaattcagtatgagtcgatttatcaatgag
gtagacaacaagaaaaaaatgaaatgtgaaataatttctcatcataatatttttatagattctcgacatgtaaaatctcatcaatcgaaca
tcagttttccacgtgtattgccaattgaacaaaatacctaatcccagtttttatctagaaattattttgaaattttaatatagtaggcatctttttttt
gactatcattgtgttctattctatttctttttatcttttatgcttcaatttcagttgctccctttttagtcattcctattgattattttgtttgttgtcctagatct
gtgccatcgttttattgaactaaactgaagatacatttcgaatgtgatcagaaaaaaatagatttcctgaattatatatatatatatatattttat
tctttttctcatatatttttatcttgccattgagcaattattcgattttcttttatctctcctttttctctcctttttcgcacacgaaattttaacatttctca
tcgactttgttcaaaatcatatcatttttaatgttttcttcttattcctttcaagttctcatttgaggtagattttccctgtgtaataaatgaataaata
aataatctgtggtttttgttgttgtctacacgctcccttttcttttgattatcgtttttttttgtctttatcaaaaatattgtggttctgcgctcgtaaaca
agcttccaaaaaaaaacgaggcgagaggagagaaaacacagattacgcacacaggggccaaagaaatagtagtgaacaaga
aagcgagaacctggcgagatagtgtgtgttagagataaaaagtatgaaggcgcattgatttttttgcggcgctgtgtctcggtgggcgtc
cctttgctgatgtggacgcgtttcgaatttttcataattcacaattgtggcctaaatttttaggagagctgtcattctttcctattttctttgtcttgttc
acacgattcttcgaattttttcgatttttttcgaaaatcttctcctaaattaatattttacagtgaataacccttcaagtcttcaataatgggaaa
ggaaaaggtccatattaacattgtcgtcatcggacacgtcgactccggaaagtccaccaccactggacacttgatctacaagtgcgg
aggaatcgacaagcgtaccatcgaaaagttcgagaaggaagctcaagaagtgagtttaatttcagctggaaaatatttcgaaattca
atataattttttttagatgggaaaaggttcgttcaagtacgcctgggttcttgacaaactcaaggctgaacgtgaacgtggtatcaccattg
atatcgctctctggaagttcgaaacttccaagtactacatcaccattattgatgctccaggacatcgtgatttcatcaagaacatgatcac
cggaacttcccaagctgattgcgccgtcttggtcgtcgcttgcggtaccggagaattcgaagccggaatctccaagaacggacaaac
ccgtgaacacgctcttctcgcccaaactcttggtgtcaagcaattgatcgttgcctgcaacaagatggactccaccgagccaccattctc

Development • Supplementary information

tgaggcccgtttcaccgaaatcaccaacgaagtctccggattcatcaagaagatcggatacaatccaaaggctgttccattcgttccaa
tctcaggattcaacggagacaacatgctcgaagtttcatccaacatgccatggttcaagggatgggctgttgaacgcaaggaaggaa
atgccaccggaaagacccttctcgaggctttggacgccatcatcccaccacaacgtccaactgaccgaccactccgtcttccactcca
agacgtctacaagatcggaggtatcggaactgtcccagtcggacgtgtcgaaaccggagtcatcaagccaggaatggtcgtcacctt
cgccccacaaaacgtcaccactgaagtcaaatccgtcgaaatgcaccacgaatcgctcccagaagccgttccaggagacaacgtc
ggattcaacgtcaagaacgtttccgtcaaggacatccgtcgtggttcagtctgctctgactcgaaacaagatccagccaaggaggctc
gcaccttccacgctcaagtcattatcatgaaccatccaggacaaatcgccgctggttacactccagttctcgattgccacaccgctcac
atcgcttgcaaattcaacgagctcaaggaaaaggtaattagaaatcagaacaacctcagaatcagagatttaattgttcaatatttata
ggtcgatcgtcgtaccggtaagaaggtcgaagatttcccaaaattcttgaaatctggagatgccggaatcgtcgaactcatcccaacc
aagccactttgcgttgaatcgttcaccgactacgctccactcggacgtttcgctgttcgtgacatgagacaaaccgtcgctgtcggagtta
tcaagtctgtcgagaaatccgatggatcatcaggaaaggtcaccaaggccgcccaaaaggccggagccgctgcaggaaagaag
aagtaataattgtgattcaatgataactgttattgtatttatgattattactcctgaaaatttatctgtttctgttactcccatcccacatttgatttaa
taatttatatctgttttcaatcatttgtgccccctttttttcctttcttcgtttcatccaatccgtttcttttacatactttaaccccccatcaattgctggt
ttcgaacatatttttatatcatacgatcgaacttggttgtttcttatccttgttaacaaaaattaacaaacaccaagtttcattctacttgtatcct
gtcttcccttgatcatcaccgtcccccccaaaaaaaaaatttcttatgttagaaaatgtgcaacctatgcatatttttgtcaaaaatgaaaata
ataaagtgttggtaaaaaaaaaattaatcgttgtttttgatcatcaacttggaatttttttgttgtaaaacagcatggtctcattttactcgataaga
taagaaagaaagaaggctctgacctcgaatggtttttgcttttttttgtttcgaaaagagcgacaatgttgagagtaaacaaaaatggtatt
gaaggtcagattgtttgaacagtgggacgatggattgggatacttttaactctctgttgattttgttcacgatttgcactgtttttgtgacaattg
gaaagaggggtttgcggtcaaaccatcaatgagtgttttgttttgcttcattttacagacaaatatag

Can-elt-1

join(5001..5301,5418..>5798,6104..>6264,7235..>7360,7574..>7729,7840..7974)

attccttgtgagtaagccagaaattattttgctttatcagtttttggaacttttattcattctttccgagggttactgtaagctttgagttgtaacaat
tgatagttctgactacagaatcttacgattctataagccgagaagcctaaagtcaggtttatctgtcaaaaaattctgagattatcgatttttc
aaaatgttatttttaaatttcatggttaaaagtttgtctcaactctgaaatttcagaaattgaaagcagttttcataatacaatcttcgaactaa
caagatattttaataaaaatcacatctaaagtcaagactagcatcttaatcttttctccggaagcctaatcctaatacctaatcctaatacct
tagcctaaactaaaaaaaaaagttacttccagctgtctttaatttggtgcgaatcttgataacctcacatcttgatttcccaccaaaatctaga
ttcgattgatttgaatcagatcacaagttagttcaagttccgggttttcctaatttttctccattacttgaaatttccagagctcctattttgattgat
gtcggcgaaaattgcaccaaaaaagataattggtgtgaacatttatttttgatcttcaaaaaaatggacagtgataccccccaaatataa
ttgggtgtaaacatacacacgagagagatttttcgatttagattaggacctacaccgtattcggtttttgagaaagaaaatccacgtccct
cgcggacttgacttcgaaaaacgagtcttgtcctcatttctttttctttttcttaatgttatttggtgcgaccaggcggtgtcctcttttttgccttgc
gcctcgtcttgaatacggtattcttgttgttgacggtcatggttatcggctcaaaaaatctacattttgcctcgtttttcgttgtcgccgatgaaa
aagaatatgttgagaaggaacacacaaaaactttttttatttcccgcaacattttcattgacccagattttgctggattttggggaggaggt
gggatactttttggaatcggaacccttgaagttgataaaatctaaattgacttgagttttttttgaagtctaaagctgagatattccaaaatt
cataggtttaccaaagatctcagaagggcctaatctaatttttttcaatttttaaacctctcactcttcccacctctttcccctaaaaaaccaa
aaaccaataaatcatcccaatctcataataatattacataaaagattaatgttgattacataagactactgtaattttctatgtctcctcatttt
tttcgccacgcatgcataatgtgttatacaataatatatcatcatgaaacttctgaaggtctcgatttcccttctttttttctcttatagtttccaaa
actaatcccatcgttcccaatttcctttttttccctcttttcctttccccaaattttcttggcaaccaaacaaaaagccgcgcgcgtatttaatga
attatatgatgattatacataaatcagagggggagagcccaagtgaagaaacagccgggtgggtgtattttggtggtgcagattttatgc
aaatatcaattggtgtgaggattggtgtgatggatggattgagaatgcttgaatgatttatactactatattatatataagaggatgtgtgga
aaacagagaaaacaacaacttaatccctattttggatgaaacattgaaataaatttacatgatgtgtagattgatttcaaagagaaagtt
gcgcggcgaaaatttggtgtgaaaccacgtggcgccgcatatccacacctgccacattggtgtgaagaatgctggaattgtttttgatg
atttgccgaaaaaaacttcgtctacagtagcatacagtatttgaaacaaattaggaatattgataggtttttggagggttactgtagatggt
gataacttgaaaagtgttttgatctaccttgaaaatgatcttctttggaaaatgtttcaagagattactgtacctgagaattacgctaactaa
gacacagtaacttctaaacgagactcgaaaagtgaaagtttcaaacagtactgtatctcaactatgaatgaaaatttctgagctacagt
agtccaacacattttccaattaatctctcccaaatccctaaattctcctacagtaatccaaaaattcgcaacatccaatttgtctcattcctcc

Development • Supplementary information

ttttttttctttccattccatgaaaaaacatcccaaatcaagtgttttattgaaataaatgtgcattgtgtacactttttctctcaatagagctgag
atccttctgaaaatcccccagagactattcaaatcggtcttctaatcgagattcatcgttgtttcgcctttttctttccgactgccttttgataggt
gacagatgaagaaggcgaaaaaaatagaagaaatgggtgaaaacaagcaggaaaagtcaattatatatgagataaaaatgaa
gttgatgtgaggttagcccattggggaatattattctcaacttatttttggtttttgtttgggcctacggtagtttttcagataccgacttccgatta
gcggattagttggattaccagcaactttttctggggcacgcacgcacgccgacttttgggaaaacagtggcccgtggtttttctcttgtcat
caacaccaaatttatgattttatctatttgcctaatttacaactcatcaataacaataataacatattttactgttcaaaaaaaatcaagaaa
aatgatttattgattggtatttttggcaagtattgaaaagtttcctgaaggcgacaagttgaagaaaaactgacgagcggaatgtaaaca
acaacaaaacggtaaacaaagacaccgtgtttttgtttggcgtcggaaacactatggtgtgacggcatgcttatcacaccaaaggccg
attccgacatggtgttaggtgtctttgatatgacggaactttggatttttttttttgagaaagattaggtataatcacagacttaataaataata
ataataataataatcacagacttaataaagtaaaactgagagttagtctaaatttcttacaagggaaagtaagttacttacacttcgctct
aattgaggatttgttagataaatattgaaaattgttaaaatgttttggaattaaactagagataaactacggttctcgaggtttgctgtaaaat
tttgtattgctcatgtcaatctctaaaaatgtctttacataaatacgaaactattcaaaaacggtcaaattctcaatggagcgtatttgcaaat
atttaactttagaagtcaatatctaggttgagacttttcggtgccaccataaacgtagactcaggtttacaattgagttagaggtatagtaa
gctatcttaaaattatttcgatattcaagttttcggcaacacggaaaacaaacacacacacacacacaaaagatccccgactctttgaa
ttaatcggtgtcactttggtgtgacggcatgcttatcacaccaaaaagacacgctcactaaatagtgtatcaaaataaattaggaagaa
ctctggcgccaacaattggcgcaacaacaactgttccacacacttttctttcatgttaatcgcaaaccgccattacaccacgatttattttttt
cttcaaattgagcaataattttaattagtaatttgaaagtaattagcatatttctcgcttgaaaactgactacagtatcccattctcgattcgatt
tcattttcgaattaggcttgtttaatggggtgttcgatgaagcgaaaaataaatgcgtggctcttttacgaaaaacatacggtagctttttctt
gaaatccgatgggaattatttgcgaaccacaacaaaaacaaaaataaaaataggaaaagattcagatttagaaaaaattaactcgt
gaccctctattttttttctgaaattaaaagaaattttggcggcatttcaagaaatttgaatttttaaaatgatgatggattattttgatgcgaact
ctttggaagagttggacaatctcgatttttcaatgtgggttttgaaagattttctgacataagggatttttactcaagctctatgtatacattaa
agttctgaaaaaatttttctgaattttgtttttttttctgaattttgttttagtttctgaagaacatctagactttagattctgaagtttctattttccca
atccgttttcatttttcctcaaacttcaaatcagacttgctgatcttgtttttatgactttttctcaaaaaaaatcccgacaaaatatctgaaccg
tattttttatttaccaaaacatttttcggaatttttcctccgctccgaaccggtcaaaaacaaccgtccaacgaaatttttgtcttgtgtccag
atcttatcctcaatttttctcttcttcattttcactcatcacaacaaataaaaatacgagaaaaatcagcagctcataaatccttctttctcttttc
tctctcccatttcccccaaaatttcctattttccacccttttcgctccgcccacaaatctttgccgattcgaagaccccgcccctctttagaat
caaggggcccccaactattttccaacactgcaaatatactgggtcgtgttatctgacattttgatttttgcattcattatatctcgaacagata
atctatctatatttttcaaagtttatgatatttttcgggggggaaatttcgaaatgtgaaaaaagaaaagggaaaaagttaagccctcacatt
gatttttacattattttttttgtccgcaaacgtggccacgtaactttttgtttggattttcattttcaacttcttttattttgtttaattttccaaaaacaaa
aaaaaattgtttgcaggcaatgagtgaatataaacccgcggatttttcactcgtcacagccgagcacgcgttatccgcccaacctgga
atgtatcaaacagcctgttcaattatggattgtgacactcaaacaatggccgatgttttatcggaaaataatcagatatctgcagttgatga
tgtttttcgccaatcaaatcgcttggtgttggtgtaggagttggtgtgactaataattcggtgaataatagttttgttcaatcaactggttataat
ccaggcaatgtttattataaagattatgcaaccacaccaactagtaagttttttttcttttttgattttttcttggccctttttcaagttaattgaaa
acgtaaactcactcactcactctaaaacaggaaattgaaaacgagtatgtttttgcaggctataacatgtttctcaattatccatcttattctc
tacccacctccacaacttctgatgctacttttggagcatctacagctggtatggttgacacttcagtcttcggtgcatcggcggcacaaaa
cacatcgtatttctacccgggatatggaggttatgatcctttaaatggaagtgcaacaacaacagcatcacttattccaagatcgactgc
aacaaataataatggattgacaccgactgcagtgattgctggatgttcgagttctgggtcgagttcggcgtcgagctcatctgccaattc
aacatctactccgagaaattctggatcaaatggaatgggtggcgggaaaatgcgatcgattagcaataatagttgtgagttttgaaaaa
tagttgaatctatttggggctactttgggaatgttagataaggcttacgagtttttttttagtatagacagttttgaaccaaaaaaagttattctac
aaaagttatttctcaaattttcaactgttttgagctaaactggaacctaagattttcaaatctgcatgttttttctgaaaatcagagcctatgaa
aattttgtgaagcttccgatttttttaaattaatttagagttttagaaagttttttttttggaaaatcgaaaccatttcccaataattccatattttcagtt
cctctgaacaccgaaggccgagagtgtgtaaattgcggagttcaaaacacgccactttggaggcgagatggcggcggacattatcttt
gcaatgcctgtggattatatcacaaaatgaacggacaaaatcggccgttggtgaagccgaaaaagcgacaggttagttttcaaaaaa
tcgctcttcctttttaaagggatgtgacctttctgataaaatcaaaagttagtgaagggaactgggaagaggagtgaaatgagggagaa
gattgatcaagttgtcggcggtggctcttgcgcaatgcatacacatgacattggccattgacaaattgcattaaatatgggaggggtact
gtaggtagaaatggtccactgtttattgattagcaaaaaagtgactttattcttggagtaaaactcttgatttattttttatttccaatttttaacaa
ataatatttgttcaaaaaaccatacactacagtaacctttagggaacccataaattttcaaaaatcattcccatatttattatcctcaccaag

cagtcaatatatttacacattccatttcaatatacagtattcctattcctttcatgttttttcagtttctttcgtctccctcaaaaatagattgttatct
cattttcgctttgttttcttcttcttctttacatttcacaaatttctttcctcacctaacacaaatactgtagagacacgacaaagtggaacaaatt
agcaaaaatcgatgaaaaattaggtcgctggaaaatgtgctctgatgagaaattgggtactgtatgagggaaaagtgtgtgttgatac
ggtgacgcaaaaatgcatccaagttgtttttaattttggagctgaaatttcagattttgagcatttctttggggaattgaagattttagtggatt
tttagcgaagttttcaaccttgaaatctatcttttaccagaaaatttgaaacggatcgatttttggtgacaattagttttacacctcaaaaactt
gaaatatcaattttaaaaaatctcatcctagtcttcgaaaaatttcgaacattccctaaacctagattctgcctcaaaaatcaatcaataaa
ttcggtttttctagagcactcaaaaacgaaccggcgtatcatgtgtaaattgcaaaacgaacacaacaacactttggcgacgaaatg
gttccggagagccagtttgtaatgcatgtggattatattataagctgcatcaggtaaatcatttaaccttcaatatttatgttttgttatttttcca
atcctttcgctaccccgataaatatcgcaacacaccaattaattgcgcaacagtttttcgcctcttttagcgcaccttaaatcttttccgactct
ttaattgcaaggcgggcgggaagaggaatcataacaaaccgagattttatctaatccgtttttcggttacaggttgaacgaccattgac
gatgaaaaaagagggaattcagacgagaaatcgaaaattgacaacaaaaggaggaaaacgagaaggatcgccgatgggaaa
aatggatttggatacatctgtaaatgcagctgctgcggccactgtttggggaatgaaggtaaatttagaaggggcttttggggattcaga
aattcataaataaaatcagaagtttcgaaaaatttaaattctaaatcagtttcaaaaattcatcaattttttttccagacttcaacaacacaa
ccaatgttaatgacatcatcaggaggatatccattcgccacaaataatttctatttcaccccacaaatcgatgattcatcaattccaatcat
ggatttctcatcaaatcttcagaaatagtagcaattattgcacgacctgcctgccctgtaaaaccaaagatcctgtgatctaaatatttaga
gagaatccttatttacatataaattatattaattcccaatctcctctgtctgactgactgtgattttattttcaaaacgcctttcttcccgaacccc
cgtcaaagatcttttcttctattgttatgttttttctcttgccaccttttttcacttcgcctgaatatgtgtacttatttttgattttcgaatttcaaaact
aataataatttctgtcttgatatatacattccattccaacccgttcatgatttgcataaagttttttttggaaccaaacagaggaacgaaaaa
caaacagctgctcagttaattatataatttatcgaaagttgtgaaagtcagttgtttttggttaagtttgaagagtgaaaaaattatttatttaa
cttcgaatattatttcgaaacgtgtcaatatgataaattgtctgcgtttttttagaaatacagtaatccgccatttttcgaaatttttttcagaaa
accgaatttttagtatctccaaatttctaggacagttacgatcatttgaattacattattccaaatttttgcatcaaatttgaaatgatttatggca
attatctacagctgtatacagtaatcttcatactgtaacatgtattttccccattattttgatctacaaaatattatgctacagtaaccagattttt
aaaatttaacaaatttaggagtatagtataccgtgttcatccgaagtttttattcacagtaaccgaaatttcatgaatatgtggaatctacaa
gattctcgaactttatgatgtctgaatcttgaaattgaaaaaaaaaactttcggaacaaagtccaacgaaaacacctgcctttccatcta
aatcattatattttttaaagtttattctcgaataaaaatgcaaaatcccaatgaaataacaatatca

Can-elt-2

join(5005..5143,5192..>5248,5300..>5408,5501..>5890,5947..>6030,6078..>6265,6322..6443)

ttatttgttaaatgcgcaagttttattcattcatttattatatgtcattacggaaaaccgaaaatcggtcgataaagaagaaagaaacagca
tatatgaataaagaaagaaaacgacgcgacgtgaagcattatttgttttctgtctgcctgtctttttcgaaaggcaaaatatcaaaatctcg
tcatatcctgcctaacttgacagactgtgcgaaaaaatttgtgtgcgagaaagaaacaaagcaacgacaatttagcggttggcaataca
tacagatcgtgtgccaaaacgctttttccggtttttgctttttgtgttttggcacaaatcatttttatcacaaatcaaacaatctagatcacctc
gtttttcttcaaaaaacttcaaaatatcatttctcctactcttttataaaaaaatctcatattcgaaaacaggaagggttgacaataaatgtga
gatttttcgtctcttcggttctttggaaattgaaattttttgaacagacgaggtttgaatgaaaaaaaatgaaaaaaagaaaaaaaaactcaa
attatatggaatgagggctgaaattattttgtgctctttttccgtttaatgaaaaaatggagcaaattgaaactgaagaattgcaaattggg
caaatcatctgaaaaaatgtgggaaacaattaatttgtctagtttttctaacactcaattgaacgtttttttgtgttttgatgttgtgacattgaaa
atggaaggtttttcccaatttttcattggaaatgaatgacttctaattaaaaagagaaacaaaattggggttttttcggaattcgttattttga
gggaatacgtttgtgtgtgaaattcattgaaggaaaatcagtttttgaaatccctggaacgaaacattgaaaaaaaatgacgaacgtc
ctgaagatttcaaattttacaaaaaaataagaaatcgtcctggtaagttaaaaatttttcctaagattattttgttgaaatttgaatcggaccc
ttgaaatcaggcaaggcccaacttcaaaatataaactcttaaaaacgcggttggtttttcggcatttttctatctcttgccgactcttcgaga
tcaatttaataactctcaaatagttttattttctgtatcgatttttccaggttttccgacttctcaaaagtttgccagaacattgtcttattcagacta
ttcaaaagttcacaaaaacaccgcgtgatattttctcaaattccaatctgtttttatattcttcacattatacgataccgtatttaatccaatatt
cccaaaaaaaattgtatttttgtactaataaaactacatttcccaactttattattcgttattcattcctaaaaataataatactaatattcataaa
caaatatccacatgttcgagggatcgacatatattactaatccaaaaatcgtcttaacatcgacgtcgtaaggatatgtaatctacctaa
aatcgtgtcgacgtaaataagaagatttgtttggtagtccaagcaagccacacctcatcctgactatttattccatctcatctcatctcatttc
attttattttggtttgatttgtttgtcaaacgaaaaatggttgagctcaagtctctcactgctccaaaagttgcgattccaattgttggactcatt

gtcttgctcgttgtttcaacattggttgctattttctatataaggtatgaacaataggaaaggaaattgtattaatctgaaattttcagaaaccc
gggtgtcactgactcaatctttggaacttccaccgaagaaccgactactcctgcgtcattttcgatgcaatccgctgatggcgaaatgga
acatctccaggtaataaattatatttcaaaatgtcatctgattttttcttctcacaatcatttcagaactcatctccacttggtcaatccgctcat
caaacaccaacaacaactgcacatgatcgttagtttgaatttgcgagaacagaaaaaaacatataaaaagcatgttgtgtttttgatca
aagtgaatgatgataaatctttcaaagtttcagatgttttatttttccagtagctacaaccacttcttcctctccaattagtacaacaacttcttct
ccagttactcaaccttctcaacaactttccacacctttacatccaattgaaccaacacaacatttaaaataattatttatttcaataaagttat
ttttctgactcattcaaaaaaaatgcaaaaaacttccctccaaaccccttttcgttctttttttattttccaaacaaatataaacgatcgaatat
aatttgctaataaatgtagggcaggcttggcgtcatgatgttactgataaggctacagtacttctatattgctggcttgtcaaatatgtattat
acgtgaataaacagaataaattcattttcagactagaaaacaggaaaaatagaatttttgttgaaaataagataaatttttgaaacattta
tcaaaaagttggttatgattatgttatcgtcagaataatgtgtataaatatatttatcattgtcagagagcccgtggttctgataccaatttttta
cagataaagttgttcccacacgtttttgagttttttttcgaaaaatattgattgacttacttactttgaactcaatttcaaactatctttcgaatattc
caccttatttgtgagatgtctcttatttccctggaaaaatttgagtagtctaggaaaacatctaaaattcagatttagaaattttctaaaagctt
tcaatttttcatttctattagattttttggggacagttagtttagtttttcttatgaaaaacatcagaattttcagtttcctcattatccttctctctggc
cctgtaattctcggcattatacaaaactgtgtatttctggaacaagagaaaaacaattttgaattgtgtcccaaagttcaatcaataaaatg
tatcgataagaagaggaaagaaaagtataagcggattacagctaaaactataagcctacataataattcagaggtaggaaaaaac
ggaatttttagaatgagaaggtttttagctgactcgagacaatcttattttaaaaaattcttcaaaacatttcactacaataaacagtgaaat
ctggctttggtttaaacttcgaattattataggtttttttatgaatacagctcaattcgataagaaatctgtgatttttgatgtttcaggtgtaggttg
ccaaataatgaataaggtaccagatttgaaacaatgatttaccaattaaaaaccaagaaaagttaaagcttttagggtcaggagaac
agtccaaaataattgttttctaacaaactaatcttctcaggccaccgttaagttttgagcctaaaagatttttttttcgactttgttaaatttaccc
gacgaatatataaaaaaaatattcattatattcagactcactaatttcatcagagttttccaaaattctgaaatctggatcagtttttagtgcct
aaccaaaaaaattggatattcatcaaatttcaccagtttcactaaatcgatcttcgtgaacaacgtccgaactgtcagattgggataatc
ataaagctaaaaggaaccaaattaaaaactgattaagaaattcatagaagataatgctttgaaatggaaagaaacaaccaaaaaa
ggttttagaattctaacagaagaaataaaaatatcagaccttcaactcagtatttatattgaaaagataatgttatctttaattgatatattcta
ttcatctgaaccatatcaagacaatcaattgtgtttttattgataaaaatattgctttttttaaatttgaaaatgtttcgaatgtcccctataatttca
gcattccccaattttgcttcccccacattgtagtataaagtcgatgaagaaaaaagttgaccggcatcatataggcaactgataagaat
accgtatactttcatgcacatcaactttttgtgtccacgacaatgcacattggacctctgaatcatacaaaaaatgagaaataatttcgag
aacaggatttcgaaaattagagaaaaatagaatgaaattatgatgtgaaataatgttgcgaaaaaaactgattaaaaatatattgatta
cgttatcgaatacttggagtgatttatcaacttgtagagtccgcggtcctgatataaaaaggtgtcagttcaacatatcgcctatcactgga
acatataaactctaaaacttgataataagtattttcgtagttcattttcatgtcataagaaattgaatcaagttttccagtgctatgcatgaatc
gttaccagaatgaagaattttggttgaaaattttagtggagaatcataggttaggaaacatttgttcaacggtaactctttgttggcatacttt
gttgaaatctgaaaatttatgactggaaaactgaaggctaagctccaaaaaacaatcttcattgataattttcatgtatttttcccaaaata
acttttttttcagaaaattcataggagttgagagaatcagtttcaatcatatcacaacttccataatatcgaaaatcaacttcgaattaaaatt
attttcctcaaagctctgggaaaatttcccaaaccctttttccagagccaatcaacaacgtttgccagctacagtaccaactcgcgacta
gaaaaggccatataaggagagggaaagagcactattattcccttttctcatttcttcattttccaatgtgtgcggggctcataatgactgat
aagataacgacagaatatttggcgttgggaagacagttgatggcttgagcatattctttctttttttgtcggtatctctaacgaatatcttgaatt
tctctattttttttcataatctatttcgcaagttcttttttttttcagaaaatggatccaagatattcgatcgagagtccctggatcgatagtatgcgt
ccaccaacattgcaactcccaatccaacaaactcaaacacatactcttatgcctgatgatgggcagagttcgatgagtgaactaacta
gaggtactagttttctgatgtttcatttttacaacattaatttattttcagttcgaactgatcctttgatggataggcaaagtgttatcggctcattt
gatttacaatgtaagttttcgactaatagaaaaataatgatactgatcttcatttttccagcaactttcgcaactggaacaaatcaagattatt
tgtataacacttcttcaatttttaacacatattcaactcaaccgcctccatcacaatatcatactttatcatcatcgtaagttggccgggaaat
tttgaattcttgaatgtttctgatttcacaataagaaaatattttcaagacatcaaactccaatttttttaacagattttacgataattcggcattta
taccacaacaacctttcgcgccagcaaatccagaatgtgtcaaatgcgcaaatccagtcatttccggaagacaagttgatggtggata
tatgtgtgatacttgtagtaattctaatgtatatgatttgtcacgaattcaaacaaactattcgatgccactttctcttgttcaaccaattgaacc
tgctccaattgaacaaattccacctccagcaccagcaccatcaaaaccaccaaaatcatcaagtaacaaaaaagctggatcagctg
gaagcacaaatcgccgacaaggattggtttgttcaaattgtaatggtacaaatactacactttggagaagaaatgctgaaggtgaacc
agtttgcaagtgagttattcaaattttaaattggaaatatcagaaatgtgaaagcttattttcagcgcatgtggtctgtatttcaaattgcatag
tataaatcggccagtgtcaatgaaaaaagagggacaacttcaaacaagaaaaaggtatttatttttcaaatgaaaacattgaacaact

Development • Supplementary information

tgaaaaattatagaaaaatgaagaatagtgaatcatcaatgactccgacacgaccaaaagagcgtaaatatgaaaaacgaacaa
aaacagaaacaactgaatatcaagcaaacgcatttgctttgaatgctcatcaacaaagccaaaatccttatagtaattcacatttcgcat
atccagtgaacccattgcattatacaggtgagatcatttcgcaagttttatttcaaaatttttaaaatatatacgtttccagaaccatcgttag
cttcattttcatatccacaatattcatcagaaatgaaaccaactattcaaatgatcaatcaagatgaagaagttaaagcagctgcgaga
gatctggaagaggaacaataattcaactatttttatcaataatttgctctgatttacggcgtatttttacctttttcatccatctcaaataggata
tgtaaagattttgatacatatttattttttttgtcatatatactcaataaatttatatttcatattgttagtttaatgctttcagaaaaaaacacatcatt
cttgtgaaatataaattacagtagcatttccgaagtttgcgaagatgagcattcttgggtaattgtgaaaacacaacaaaaagaaataat
cggaatcaagaatgaatggaatcagaatctcggcattccgaaaccgatgggtttttcggaatgagactaacgctggaacacattccg
aaacttttcgaaatatctgaatgttttcatcgaccttttaaggttttaatttaaaaattagtgaaattttcaaataattatgaaaaagtgatgaat
tattacccaaaaaaatcatcctataattttcaaataaaaaaaatgtttcc

Can-elt-3

'a' isoform: join(9306..9424,10061..>10331,10439..>10533,10581..10713)

'b' isoform:
join(7159..7247,7294..>7442,8921..>9136,9319..>9424,10061..>10331,10439..>10533,10581..
10713)

tctacagtttctagaagagttcgttgaagagtatagatttcttcaaaatttccaaccaataggtcatagtcgttcgcatttactctgaaatttag
gtaatgttggatgggtgagatgaaaataatttttcttggaaatgtttttccctatttcaaaataaccgttttgacctcatccaatgcaagattca
aatcgatttgtattttacaccaggtcatttttttgagcgaaaaattgaaatttgtttatcaaattcattattattttgtaattaacatagtttgagtata
attatcacaaaaaactagaaattctatatattatttcgaaaaattgggtgatgacctcatccagtgcaagatacaaatcgatttgtattttac
accgggtcattctttgagcaaaatttgaatttatttatcaaattcattattatctcctaattaacatagtttgagtataattatcacaaaaaacta
gaaattctgtaaattatttcgaaaaattgggtgatgacctcatcgagtgcaagattcaaatcgatttgtattttacaccgggtcatttttttgag
caagaaattaaaatttgtttatcaaattcattatatatcttgtaattaacaaagtttgagtataattatcacaaaaaactcacaatccagatttt
ccaatcggtaaaagaatactactaatcgtcctagtcatattatcaatatattccttctcatgctttgtgtaatcaataagaacatcagcacga
taatcaggctgattcaaagcaccaacaagttctgaaaaatagttctttatctctaaacaaaacaatttcaaaactacctaccttttgcagtg
gtatttgggacagatctcgagcgcatgagtttatctggaaaattttttttaaattagaaaaatgaattttagtaataaagaaaaagacatac
ctttttcagtgatcagattcacataggcacatggaaaccatccagtcaaacctccatgtgttccttcccaccatccaccatcttgttgttgag
tgattgtgataatatcatttttatcaaaactcaactgaaaatttcgaacaaaattacataataaactcaaaaaatacccacctcgtcattatt
cgttccctcaaatttgaatttggcacgcgcagtcaatggagatccagaatctgaagtttccattttgcaaaaatggaactttttcaaagtat
acaattgttgctagagagacagagacaaacaagaaaattggggcagagacgcagcgcgtccctgtcatattttatgattggggcgtc
atttctcctgtcccttttttattcattgtttgcactctcctatttttccattatatttctcatagtttatttgaaataagtttattcctttcgttttgttatttttcctc
caatccaattatttctcgttatatttttctgataacaaaaattatatctttgaaaaaacgataaatttcaggtaaatggaatgagatcattagc
gaatggaaagtttggcgcatttatcattagatcagttgaagaaggtggaagaacgtgtgcgaatggtcttccgttagttccacccaacgt
gcggatgcttggaaggtgagtatttgtatatttgagagtagaaacagtaaagattccgagaatttcagattccaacatatgaaagcattg
cgacacgagaatttgtgccaatatttggatctttttcgatgctctttaggtgtgtttttcaaaggggaatattcaagaattttatgtaaaaaaaa
tttagattgattcgaaaatttatgtcaattcttacttttcctccactcacgagtattaaaacatttttttttccagcaaaaaacttggttatcattgtat
ctgaacatcatttatcaaacttgcaacaactttgtaaaaatacaaatctcacgtgagtatcctcccccactttcctctttttcttaacaactttct
catttttctttttcagatctttggaagttattgatttatctttacaaatagcaaaaggtttggaatatcttcatgaacaaggatatgttcacggca
atttggatatctcaaatattgaagtttttcaaataaaacctatcaaaattcgtttggcaagctatgccttatcgtaagttagaaatcaaacat
atcttatcaattcaattggttatcaaaaatctttcagatatttgacagatttcggtgaagattccgaatcacttattatcaattcgccatattgttc
agctccagaaattttattgagtggttcatgctcggaaaaatcagatgtttggtcatttggtatggttgttcaacaaatgtatacaaaaacatt
gtttagtgattattggtctggtccacaatttatttcgattatcgagaaatgctgtaaaacgataggtttttttctggtcataaaataatgtcatcaa
aattgtttttttatagagaatgctaatcagtgtttgttcaatacacttcaaaaaacaactgattatgagaaaaatgatacggtataattttttttgt
ttgtttttttttttagaaaaaaaaacttcataatttccagctcgaaaaaattatcatgaattgtatcaatgcgcttccatcaaagagaccaaca
atcacagaaatattgaaagatctgaacctgaatgattctgaaattgaagattttgaatatgtagatataaatgcgttgcgagaagagatt
caggagagaatgaacacggaaaaatgttttattttatcggaaatgccaattgaagacgcgtttttttctttggaaaatgagtggagcaagtt

Development • Supplementary information

gtgaaagtattatgattcgaaaaggtgttatacaacttcgagcaccggtttcaactgttccaaggtaccacttttcaggggaggactaag
gtttttgatatgggaaatgaaatgcaaagttggatttgtgtattccaaaacgtcagattttgaaaaataattatcacggaaaatatttttatttt
cagctcggtgatcgaagattttgtgtttttcggaaacgaggaaagtcgtcaatttcagtttgacgattgtcttgttattctgccagacgacaa
acttctagagaaaatatcaagtttgaaaataaccgatgtcataagaccaatatattttgccagaatcactgattcaacagaagatataat
tattgacagaaaaacatcttcgctaactgttgttatcaaggaaaaagatgtgaagtatcaggtaaaaatgattatttttgcaatctcatac
atttattcttcaggcgcagagaatgtcaattttgaggcatttagtggattgcatacaatttgacggaatggaagagttattgcgaaaagag
gcgtttgtagatattccaccagttttgagatcaagagtttggagatctttgcttcatgtggaggatgaagattgtttggatttttataatttggat
actcttgcaccacatactagtgatcgacagcttgatgttgatattccaagatgtcatcaggtagatgctgaaggattttcagaaaaagtttt
ctaaagatgtagaacacgttttcttctgaaaaaaaatcgataggaaaagatctttatagaaaattttaaatttctcgcatttaaatcaacaa
acattttccagtacgaagatatgatgacatcaccagcagcccattctagtttacgacgaattctaaaagcttggctgttatcaaacacaa
attatgtttattggcaaggatgtgacagtttagctgccccatttcttctacttaacttcaatgatcctcgtgagtttttttctagatatttgaatatta
gattctcaaaaaatttcagagattgctcttccgtgcctaaacgagtttatcaatcgatatcttttcaattttttcttacgcgataactctgctatca
ttcaaggtatgatttgatcaaataaattgtctttctaatatcatttttttctcacccaccctacaattcgttttcattttcagaatatcttgccgttttc
aatcacttacttgcatatgtcgatgcccaattatatacacatttagcagatctcgggtttttcccggaattgtatgcgattccgtggtttttgaca
tgttttgcgcacgttttaccaatgcataaattgtatcacgtttgggattatgttctacttcatgatacatcatttccactgatggttggattggctgt
gatgaaacagttgagaccaagattgattgattcgtcgttcaatgatgctattttattgtttagtgatctaccaggtaacatttttttcattcaaag
cattgttcagaacttgtgtagattactggttaaagacctccccatttccaaaaaaagtatcattcataaaaaactttaaactggtcttgaga
attttttagtattatcttatgacgatttattatcactattaccaatactcaagatttgtttttttttaaacttatcaaaacatatgttgaagtcgtgtttc
aaatgaaacaaatctggaatctatgaaagtgttcaaaaccataactatttttacgtcaatttttttcaagtaacaactgaattcttacagaaa
tcagaccaattgatctagggaaaaaatctagttaggaatgtaactctgtaagaaaaatgttcagtctcaatttttaaagcccccttttcaca
gcctcaaaattttgctttttcaaaacagacaatcagcaatagattatttcagacagtttttttattcaaacttttttttctttaaattctgataagatta
tttagcttctcataagagacaaagagaaaattgtagtctgaaaaacccctgataaaaattcgaacgttttttttctaaaaaaaattgtttga
acaaatatgtatatgtaaataaaaaatggaaaaaggaaaagtacaacattttcagatttaccaatcgataaactcatcgaaaatgctgt
gtattattacaaaaccattccaccaagttgcacttttcgagttcacgatagtccatggaaaggtaacattttttttattaatttttcaaataaatat
taaatatttttccagccacatattccgacgcgccttataatttgaatcgagaaatctccccattcaccgttgctgaactcaaagagctcaatt
gtcccagaatgtcaaaagatgagttctgttggcgtgtacaccagcaaagtattgttgttattgatatcaggtcaagcatggagtaagatct
ttttccttttttttagagtaatagcaataataattaaaatggggagataatatatatgtgaacaaagagaattgtacttttatcattgatgagca
aaaatgacaacatgtggagggtacttaggtcaaagttttgtgtgcgtgtttttttttttgttttgccataccgcacaaaaaatgtgtataaccaa
caaaatatgatgataagaaacatcggaggaaaaatctttttttgaaaaaaaaacacaaggaaatatttttttatttattttgaaacaaactg
aaactttttggaagatcaaatcaacgtgtaaacaaagtacaaaagactaacgatctgagaaaacaatttcatagttgattctagttatttc
tagaactttatataagtttccaaatctgaaaatgttatttatcatttcattttgccactttttttgtttacaaaattgttccatattcttggaaaaactta
aaaaaaaagattcaagatttctttgtaactataaaattcctattcagcttcaaccgtggatgcgttattcgatcgatacattacggaaacattg
aagatgacacatttaaaaatgtaatgaacgctttcaaaattgcacaagaaaactgccatccaatttgtgtggtttggggaaaagactttg
cggccgctgtaagggtgagacactttttgttgtaaacgatgtttctcgaagaataacatttcatttttagttctccggaaaactcgtgttcaaa
tcaatcaacggggtttgtattctcgacggtggttttgaaaatattcgagatgatcgatctctcatcactattgggcattttaaaattatcgagtt
ataattcatttttttgttttcacgcacgtgtattgtttccccaacggtttttttcagcattctgtgattttacctactttttactacccatttttgccaccaa
ataaaaaattacactttttttactacgcagtttttttgtgtgtgtttgtaagtttgcaagatgctgctacgaattaaaaagattattatgattatattt
gaatcaaaatatggttctgtgtaaacgaaagaagagacacagcaacaactgtgaaggtttttcggaagatttatggggagaaagtgtt
ggaaaccggattggagagacaaatacacgcaccccccgaacatacgatcttccacctaaatttcaaattgagagaaaaaacattgtt
acctaaaaatagtagatgaaaatcaaactagaacgcaactcaaaactcctggtttcacagaaaaaacatgccctctacaatcaacg
caacacacaacgataaaatggtcagatgaacttttttctcagaaaaatataatgtctttatgttttttttcaataactgccaaaaaatactcgc
ctcttttttttcttatcaattttttttgtcggggtgtctcaaccaaaaatttgtgaaccgaaaacgaatgtcaatcaagctttttttcttttaacttttcggt
gtgtgcttttcccaaaatgcttattttccttgatgatttacaaaataagtgtgccgctttccagaaggtgtgaagaaaaagcggtgcctttttttc
aattatcaactagagaacttgggaagtgggcggagtctggtagctcacttgctcctgggaaggtagcagtactatatatagtgcatgcg
caattcattacctccctctttcattaaaaataatcttaaatattttttttgttgatgatagataattttttttgtagtatttgcttttttactgactgttttgag
gtctgctcaaaaacaacaaaactgttttttttctccatttttggtacctttttcgaaatcgatcgtttattatcagttgtttttttcagacactaacaaca
aaatgaatactcaaagtcctctcgggattcaaacctactacccaaactcaccgcattcaagcactgatacatcatcacgtggtaagtta

Development • Supplementary information

ctgtaactttttctcagataatttaaatgttctataaatttaagatagcatgatgaatacaccaattccaagtgatacatattcgccagcaaat
tatcatcatcttcatcatcaacaaaatcagaatcacaatcaaagtcagaatcataatcaagtgcaaaattatcaagatcatcaatttgag
caacagtatcaaggtaaattttaaaatatagttttttttgaaaacttgttcaaatgttattttatcatcattacaattttgatcgattagttcgaaaa
aatagacaaaaaacacacaaaaattctacttcttctttgccatttttctgttgtattgtcatcgttcgtctttcttcttcgctcaaaatattgttgcttt
tttgtcaatttttgagaaaaaatatgtaattccaaattttgttcttcaaatgtgtgtttcttcgaaaagagagacacagaagacagtaatagtt
tttcacggtcattcttctctatcacaacttaatcatttttcacttcaccgtttcttcttcgtatttttgtgttaagaaaaaatagaagaaagacaaa
aaagaagagaagactatttagaagaagaagaaggagaaaaagaagatcgggaatttggacggacattgtcgggtcgccaatttgt
aacccgtccacacagtggccgcaatgttgttactgtgtattccccccatatctttttcctttttccattcaatcgttcttcttcttcttcttcgtatatc
actgatataggggaatggaatgattttttccatccttgcgctcactttcgctcactttgtttccaactattttattttgttacgatttttatgcgtccct
ttttaaaatccaaaagtttctcacgatttaaatttgaaaaggcaataataagaaaaaatctgtcgagctgcactttacactttttcccgttcc
cccccaattttttttgctttcgcaaaaaatgaaacaattttattttttgaaaaaatggaactgtattgattctcgattcgacttttagtttttttttattgt
tggtgaaaattgtatttttaggttaggttatgttaggttaacacttcattctctctctctttttcaaattttctctcttcatgttgattgtgcgtccggcc
gattggtcgagaatggggcggcgcaacacaagttgcggccactctctactaccgtagtaatatactgcgaatcacggggcattgtcttt
attcggtctttttcgatttttctcgcattttcctattcttgaaaaaaaaacattattctcaaaactctcctagtcattgtgtcaacaatgaaactctt
cgtttttttccaacacaaaaattcttaccatatttgcatttgccatcccatttacatcgtctcatccttgttagcacaccgtttttacgagttggc
atttttatacgtttttttttggagaaagaaaggtgttgcatttttccagctgtttttagaacggtattttgaattccatcaaaattattttattgaatt
tgttagaaattttacaatatttttactgaagattctgacacctttctaccctttttcagagccatgaattcagaattttgaattaaaaattctcaag
ttcaacatttccaaaataaataaaaaaaattgcagaatatcatcaccaccaagaggcgcagccacaccatcaactaccaccacaattc
gagccaacaaatagttcaaattcacgagcttattattgtccatatccatcaacaacggagacaatacaagctccggttactgtcgcctct
gctgctccccttccagctatcgctcatttaatgaaggaagttcagatcaaagaggagtttaacgaatattatggtaagtttttctttttttcctcg
accctatatttatttatttttattaaattattatataaactgtatacaatcttgaccttctctccacccacaggaaaagatctttatcgctcttttttttct
ttcccgcaacaaaaagaagatatctcaattcattcctatacataatatgttatttacagctcccaatccatcaatgaacgactatcgcgttg
aaaagattgccaatccgttggctgacccatatattcaaattgagcaaccaatttataccgattttgcaaatgcgtaagtgaaactttgttca
ttttccttaacaatgtctataggggggaggggggattcgacaatacacaatcattatcaaggtcaacttctatcatcaatcactctttttttctaat
tgttaaataagtatgattgaagaatttgaaataggaaaaaacctgggtggaaattgagaagccaagagatcaattttatttggatttgaa
aaaagagagtggtggatggttaaaaaaagagaaaattagaactttttaagccaaaattcgttatggttttgttagaaaactggaattaaa
aatagtgattttaagtgtgattatgacaagcagctaaattgatgatagagagctttttagcacttttctttatctgaatacttctaaactttcaaa
aaaaaacttttcattgaataactcatgttccagaatttttatgaacacatttctgagtaacaaatatgaaactattctcaaattagagtagag
ccaactttttttcagatggttcaaaaatcgaaaagtgatagtgttattttaattttaaaaactctagttcacaattttgactgtaactgaaaacc
agagttccattatcacaacattgttcttatctcaaaagtaacccccctaaatttttttcagtccgatgctcaatcaacaagatatgctaaaccc
gatgaatttctccaccaactacatgggagccacacaattctcccaccaacagactgtgcaactcaatttgctagatctcagcggcttctc
cacattcaactcgtgtgatccagctctaccattaatgcaccagtcaccttctcaacaaaattatgtgatgccacattcaactttacaccac
ctccacaagatccattggtcgctgaacaaaaaccacttgtcaaaaagcgaatggctgtaagttttgtttttttttttttgaaaacgggttttatt
gattttccttgttgacagaaaacagtgatttcagaaaaacaaagagttcttaattcttcatttttcaggccgtgcaatgtcatcaaaattcaat
ttgctcaaactgcaagacacgtgaaactacactttggcgacgaaatggtgaaggaggtgttgagtgcaagtaagttgttctaactcgaa
aaaaaaaattaaccaaatttttttgcagcgcttgcaatttgtatttccgcaaaaacaatcgaaaaaggccgttgtcattgagaaaagatg
gaataatgaaacgaaatcgtcgtccaagaaacgaatcaccaaatgggatcagaagacaagccacatgctaaacttccccgtttccc
aaaaacaaaatattgctgatattcaaacagaatttgtttgttgatttttttctcttctaaaaaaagtgtttgcccgacatgtacagatgatttctctc
atttattattttagtatctatctatagtcgttgctgccatttccgccccgaaaaaatgtctggtgtttttacagtctcccaaaaatgttttctctattc
atagatcatcaaaatgccatttcgtcatgatcttacaaaatactctttttttgtgcatttcttaattaattgttttctgctcacctacttccaaaaaattc
ccaaaaccattcccaaagatgtttttttctcccaatttttgttacaatattttccatgacttgcaaatattatttttgtttgtcgaaaatttgcctatttg
tttgtgtattttactggtatgtttttttttccgttttcccttaaaattgtttccagcattaacccatttttttctgtcacataattttaacatatctaacataat
cccattaggcataaacatatatttttccaaaatatttgaaataaattgaaaactttgttcatttttttgtttttttttctcgttgatcacaaaaaaac
ggtttgttatcaataagcgtttttaacgatcttttctttgttgcattttgtttggatattgttgatcggttcataaagtggtcaaaccacaaaaatga
tagattatgggtgatatttatccaaagatttttgttgaagtttgaacacaaattgagagatttttgctatcgtattcgatgaacgattttgattgat
tggaacagaaagttcagggtagtgtgggtgagataatgtttgaactcgcgatgtatttctggtttccggattctataggaaatttattcagac
ctattcctaacattgttcattcacatttttctgacataaatcaaatgaaactttaatttataccaacaagaaataaatcccaagttgagcttct

gaaaaaactcggttttatgaacaaataaaaactcatgaggagatatgaaaaacccggagaaaatgttgtgagtgaacatttctttcata
agaattcttcgttaagtttgagtgaaaatttgaaaaatataatgaaaaactttgggcgtcccatcagatcagtcagattcttcttgatgatttt
agttattatgtacaactaaaatctctaacgttattgttcaaatccatttcttgactgtaattatttttgacatgaacgaagaaaattttagataa
aattagagtgccaattcaataattgtaaattttacataatgtcaaaaacctttaaatttatgaaacacactttttttgcatgaaaaccgtatttttt
tttaactccaaacacgcgagatatcgaataattgtgatgaggagtatatattcctatttgaatatacgtcttccacccccctctctctctctctc
tctatcacaaattctctcttcattctccacactttctcatcttctctgtacaatctgtaacctaacactttttccttcatatactcataagttttgttttct
cgtcatctaacgagttttctaactgtttgtcttatttttatgatgcgtatgatggtttttatgaaaaaggttcgaaaaattcaatctctgtgaagctt
ctaaaaaaacttcaggattcttatcattctctcgatatttttgaactgccaagtgctctactttttcactcaattagtctcttccaaaaatgtaaa
tattgcagattgcccacaacaatttttcagtgtcaaagtgtaaagtttgatcgatgtctcagcttggcgtaagttctcataatggtggaagtat
tcaatccttacctaaatgatttcttccttcaggcaaacaacaactgtcacgaacaagacgtttcggaaaaccgttccaatgaaagttttga
aaatggccacagtgaacacgattgggatacttcgttcaattccgatgtttcttgcgatgtaagttgtgaatataactacaaaaaactaaca
tatttttatagtacttaccggttcaaagagggacggccgacgatccgatttgtttaagtgatgactcagtgagtgataatataaacataact
tataaggttattgaagtaaaaccccccatttaaaaataaaaatatcctattcactcatttgtaaaaatgtagagaaatgtcaagaagtgtc
gagaaacctctcttttgttgagtttttcggcatttctcgacacaccttgaatatgatatttttgtttttg

Can-elt-5

join(3182..3683,3753..>3853,4391..>4654,6982..>7053,7108..7263)

tttatatatatatacatattttctcgagctcattcattttcattattttcattacttcatagccgctgggcatcttttcgctgtttgtttgtccaatcggctt
catctttttcatatcagttgggaaatttggaaaattaatttatgaaaaattcaactacttcaggcttagagaatttttaaagcccgaaaattttc
atctaaagtttttttattgggttgataagcctgaattttgcctaagttttgaaaacaagcctttatctgaattgggatcggacctgttttatcagga
agtctcatcccaaaacaggccttaaataaataggcttaacaaacttaactagcctctaggcgctgatccttgacacctagaatgtctag
gcgcggctacttaataactaggctctcaagacgcggatccttgtcagaggggatctctagactcgggtcctagacaattaggttatctag
gctcgggggcttaacaacccggcttactagacttggctactagtcacgggagctcttaaggtttcatgagttaaaattttcaaattattttaa
cttctctgaactttattgagctccatgcaccccccccccccaaataatttcaaccgattttcttccaaatcacatgacctttcataataaccta
attgtcgtcgccaccaaaaactcccacaaagagcctcaacaattgtgtgtcgatttttaacaaaacgtgaacaatagtcgcctggggtt
acggctgatagaatatttaaagagtggctggccagagcgggcacccattattattatcgaggttctttctgcgtctgcccagcatccggttt
tgcttttgttctgttttcatgctctcgaagaaaaggtttttgtttggtgcagaaaatttttatttatcatgacatagaacacaaatgcgctccaac
gagaaagataattttcacgtgaatattcaaaaaatcattttttttttgtttctctacacctctcctctctcatcagattgcatttatcactaagaaa
tgtgtgaagactaaaaacaacatcacaaaaaggcgggaagatgagatttggcaggtgttaaggtgtgaaaaacgaacagattgtg
gatacttattgttagtgttattattgattggggatttttctgtatgttgcgtaatttgggaagattagtgggcaaggtgttagccaatcattgtctttt
tcatcatttattcacatcattttatttatgtttggttagacatcatttttctgcatttttaatcgtattttttcctgctagcttttaaaggaaatttgaagca
tcttgaaaaacagcgcccgtggctcagtggcagagcgttttgctgtggcgcgcaaggcgcgagttcgatccccgacccctacaaactt
ttttcttccatcatttttcgcctatcctctatcctatcgacaatcttaatctcatcacactcaccattcacattcacgatatttttttcctgacagtaatt
cctgtcatataagatgcggattatctctcctcttcgcttatttttcgcgcaggtgttgatattatgataatgagagcgttggaaggatttacgag
gagaaaattgcatttttaggagggcctatttggtttacgaccatctagatattgttgtggacaagtttcgaggttatttggagaaactttgaat
agaagatcaggattttgtaaaattcaaattttagttctatcaaaaaattcaggaaaactcttcgatgcaagttattatttttttttgaattttttaa
acaaaaatttaacattcagtgatagatatttctaaccctcttgtaaattccactcaaaaaatcctcataacatttcacggcctctcaattcga
taacaatcattttggtgattcgattcctcctcgaatcgggtcgcataaaaacagaagaaaagaaaggctatgttgttaacacgcgtgttat
aggcgaaaagattgatattagataactccgtttttttgcctccctttgcgcacagatagatcgtaatcactccgacccctttttagcgatttgta
atcgatagaagattgctatctcacacacaacataactttttttgtttggaaggagacaacacaaaacgaccctccttttttgattttaatttattt
atattaccgccctttagatattctgagataagccagaagaatcttcatcttcttatgaagatgaagcttttatttactatttcacatttataataa
atacaagttcatttcttctcccgaaacaccgcataattcgaaaagggcggagctcgcaattcgcatttcgaaatcggtaattgttcatggg
cggcgcttatctgcaggcccccagccactttactcttttttttcttgcttgatagatatatttttggttgtcgttaggcacacgggatttttattattta
ttttctccccttcgaaatcttacggtagtttattatgaatggatgagtgaatggatatttttgtggggagaagaagttactatggtgaatgttat
ggcaatagttgattttgtattttattcgttttcgttttccgaggaaaaatcatttgaatttttgtaagaagtaaaaataaaaaaatttcaaacccgc
aaaaatgcattgatcgctgcgagacacctcgccaaatctttgtgtgcctttaacaaaaataccgtactctatcgaaaaaatatttttatttttc

Development • Supplementary information

tgcttttctcaccttcccgtcgcgcgttgtcaacacaatttccaccctcgccagagagtgtataaacataaacctatcagattacgtccttct
gctcgccaaggaccacccccttttataccaccacccaaaaatgattaacattgccaggtgggtggagctcgaccccgcccacaattg
ccttcttccctcttctcattgttgtttctctctccttacatgtcttgtttgttgttgtcttgtcttgatttcgtctatcttcttaatttctattcatatcttgagctc
tcatcaaatgaagcttcttgctttatcatccattttcttcccttctttttctccatttttttcctttttttttaatatcccacgcggattttcgccataaatcca
tctttcattttattttttttgttcgcttctattcaaattcgtattccagggatgacacaaatcgagtcatcatcaccagcttcacaatgtgcagcga
catcgccgattcctgtcgaagatccgcccgctgaaactcccgaattaacagcagcaccttgcgcaggttgcgtacaattgcaccacga
aatcaagcagaacgtgcaacaaatgatggataaattcgatttgatgtatatgagacttgagagttttatcagtgataaagagaaggatc
gcatgtcagagattggaagtgaggaaaaagatgcgccatcaccatcaggaagtcgaagctcgccaaatctgcaaaatggaactgg
aaaaggtggagccggttcgcggaagcgaaaaccaaccaaagaatcgattaatcgtgttggcgcacttcttgaagccgcgcagaatt
cggaaaatttcttgaatggattatcagatgagaaacgacagatctcaacaccagtctcggctagttcaccatttccagattttaacaatttt
gcaaacggatttatgtgtgagtttttttaggtttacatcctagtccccctggattatttttcactgatatttttatttgtttccagtcgatccaatggct
caccaacaacaaatgatgaatattctggcgatggtccaacaacaacaaggacccgctgaaaatgctgctggaactccagccaaac
aagtgaaacaggagaaagaggaaattatcattagacaaccgaaaatcgaaattagcgaagagcaaccatcgcaaagtattatgg
aacaattgacggcgcatttgaatggggtaagaattggggttctagagtcaagaccttaagtgtaaggccctaaatttttgaataaacca
atatatcttggctgaataaatctcaccttataatcttttaacacagcccgccgctctcccaaacaaagaacatttttttgttcgcaaaaaagtt
tatgtgaccctctattctaacacatttatcttttttttttggatagatgtttcacctgccgccgccgctttttttgttgttagttttgcgaaatttatcgcg
cgcaaaaaagttttgggtgatagggaaatttgaaagttttggggggttttaaaggtggagttcgagataaaaattgcatttttgcattttctca
agccattttacacgggttttcactgttttcttcacctttacgtaatttaaaaaatcatcccaaatgtttttttttccagaaactccaagaaaccca
acaaccaacatcagccacagccaacatcaaaacatcaccatcgcaagcttctgccgcgccagccaccacaccatccaccaacaa
ttcagcagccaacaatgccgcttcgaataatgatgaagatgagttgagtgcgtcgttgtcgcgatgctcgaattgtatgacgaccaaaa
cgacagcttggcgacgcgatttgacgggaaaattggtgtgcaacgcgtgcggattatattatcgattacatcgggtgagctttttttgaaaa
ttttttgggagaatcggggatttttggatgaaatggtggataaataaggattttccggtacaaatttaaaatcataaattttcaaatctccaa
aacatataatttcttggcgcctcatacatatttcaaaaaagataattattattaggcagcagctgattggtttttctgcctcgctttttttttcgaaa
aaaaaaaggtcgtttcgactaattatcgaattgaataggtttttttcgggaggtaaaatatgtatgttcaaggtttttaactaccttagcctaga
catccaaaaaatagccattaacaaaaacctgggcagctgatagatatagggctctctatttgtgcacctgttccccccatccaaatctac
cgtatttatatccgcttcaatgataattactacctcttttccccttttgtcaagtcaatcacatacagtactctcattctctttttgtctgtatataga
gaaagagagataattagtgtggtcggaggcggcggtaagaagaaaatgattacccatcgggtggaaatggatggatgatatattga
attgtgaaaaggggagcaattagtgaagatgactgagtgacacccgaaacagaagaaaaggtagaaaaggaaaacaatatgaa
taagaaaagaaaagaaattatgatgaagatgacgatgatgactaccgacagatgaacaatgtaaacacctgcaccaggtggacg
gggggaactcttatggaaaggaaaagaaaagtgcatggataaaagggtcccccggaaattgtcagggttcagagaatctggaggc
tgtcagacaagccagagttttgaatttcaaaaaaaaaaatcaaaagggtttttaggcgatgttacttgagaagtaggttctctagacacgg
gtacttgacaattagaattttaaggcgcgcttccttgccagtttaagtctctaggagacactacttgaaatatcagctgtatagacgcgagt
cctatacgatttaggtttctaggcgttgctccttgacttcaagaatttctaggcttgcctgcttgacatggaagcttttaaggctctgatccttgt
cagggtcgatttgaggccgcgggccctagattagcaaaacctttaaaaaatttgaattttgattttccaaaattaaaaaaaaattgtatctcc
caccagacttctgaacttttccacactcccagcccaagttactactgtactctacctcctttcattgttgattgacctcaattattgttatttttcttc
gatgttaataacaacaccgataattgaacctaagatcgaaccgaacatgatgatcactccggccgcccgccttgactcaaagttcttttt
cccactaattaccgaaatacaaaaggtggtacaaccttgaggcgaatcgtgctaatttacttttgatgcacttttgcaatatgattcgatgc
ggtcattttcttttcttccaagatactgtagatattatatataccgacggaacaatagaataggttaggttaggttagaaaataaaacgcaa
agataattagagggacggtgttttgcaatttgagacgaagaagaagaagacagtgttttggaaacgttctattatataacaacagagg
gttccgcaggcagcggatattgaactcgacaccttgcacttgacagtgtctactgttgccactgtgccatttaaccactttatatccacaaa
accatgatttttatcgtttgtctgtttgacacggttgtttagcagctgtctttgtgacatccaatgacctcatattcacaagatgacaactaaga
taagcgaggtagtagttacaagaaaaaggaaggaaagagatgagtagaacgaaccaattatagtagtattagttgaagagggtgtt
attaacgatatgatgattgactaggggccggttttggggagggcacgcgtgcgcacgcggattataagatacggtagataattgggca
ggtgaagaaaaggaaaatgatgatgatgatgatgaagaagaaaaaaatgtttggttgacgaccaaaaaattgaggattgatggggat
ttctaggcataaaaaatctgaaaattagggcacaaaactctcagctaaattttgacctgaaaaccgtaaatttttaggttaaatttggttcta
aatatccccaaatttactcaaatttgagctgaaacatcttaaaattgttttattcaaaaagcttcaaaactataaaatcttctcactaaaaaa
attcctaccaatttttcaaattcaattttttttgcagactcatcgacctgttcatatgcgcaaagatttcattcaacaaagattccgccgaaaaa

Development • Supplementary information

tcaaagaagatgatgtaagtcacccatattttccatatgactttccctaacttctcaatattttgcaggtatcaaatcaaaacgcagtgttca
gtcaacttctagccatgtctccaatgacatctggaaatggctcaaatacaccaaacgccttctcatttttggagcaattcaatcaactcaat
caagttcaggagcaattgaatagcagtgcacccgtctaattaattaattagcagctaaccctaacacaaaaaactcacttaaaaatca
aattaaatatttatttttcacattccctcacccactatgaaatttttcctttttgaccaccgcgtgttttaactgtgagctttactttttcaattgtttgt
tttgtgtgtgtggcccttgtagggctctttttggtctagtgcaggttttactggcgcatttccttccgtccaattttgatcgtcatttttcacttttcctt
gcatttttctctctcacaccctttctttttaccataatatttctctcatttaacgaaaagcataatcaaaaaacaaccccaaacagtaagcaa
tttctcatttttttctcgttttctttttttctgtaacatatcttatggttttttattgaaataataacaataaaatgcattttaaattgaatttgtgctcatatt
tgaatttcagaggaaaaattgcagattttcttcacgaaaatttttttttttttttttttaaattaaaaaatgttatttcatacacgctgtccacaaat
gaaaaaacgctgccttgtctgcactctgacacacggcatactaaactgtgtgtgtctgttcgctctctccgatcgcaacactctctcgtcat
ctttttttctctcggtttcttcagcgggaggcgctctctcgtttttttcttttgaaaaaattttagctttttttcattgaatactctgaaa

Can-myo-2

join(2457..2519,2601..>2870,2924..>3574,3636..>3795,4088..>4359,4412..>4582,4629..>5474,
5530..>8066,8120..>8270,8319..>8527,8575..>8656,8707..>8988,9047..9187)

cactccagtaacaatccagataagaaaaactgagaaaaatgcacccaatacttctgaaaaagatctagaattgtaaaaaagaaata
ttttacattattttacaaacctgctcgatgaaatccaaaactcatagtttgagatggttttcgtctggcaatatataatgaaaacaatgagatc
aatacggatgcaaaatcagttagctgaaaagtttcctgttcaatatcaaacaattgtaattttcaaaatcttaccaaatgtgcggcatctgt
cacaatcgcaagtgatccagctaaaattccaccaaaaacttcacatatcatgaaaaataagcacaacgcaacagttagccaaagta
cccgagtggctggaaaaaaagttcaaaagatttttagatatttgtgaagatcaaagagatacctcgcttgttagattcatttgattcaaga
gaaatttcatcgtgacaatgaaaattgtcattatcgtaggttactgttgtttttcggccatctgcggaagcatcgtaggtttttaatctgaaaa
gttttataaattgaactatcgatattttttacaaaaaaataagttagcaaaacttgaaattatgttatttttatgacccgtctttctataaaacatt
atattagagttggtttttttttccaaattcaccgaaatatattagtgctgaaaatttgagtataactttcatttatccaaaacataatgttctgtgat
ctttgtgacatttgataaatataaaaaccatttgttctacgcaagttgttatatcatttttttgctagctctcttattttattttattttcacaaagcaca
aaaaagaaactgtgtgatatttcggagtcgacctcatttcatctattcattcatttttttttcattgcgcgcgcgcgagctgctccacatccaca
tctcaaagtaaatattacacgaagaaaaaaaaaagaaaaagaagaagaagaacaaaaaacgaagggcgagatttttagttgctatt
ctcaagataaactcacccattttcaagtagcggtgatcgctcggaaatcatttgaaaagaagataagcgaaataaatgtgcttcaacct
gaaatgcttattatttttgtgaattattgaaacaaagaatggaaaacggacggaaaaataaaggaataatgaacaaaaatgttatttatc
agtttattttttttctgactacgaatgatgattgataaaaaaaagttgtgtggggaggggtcctaattgtacattgtgacataatcatatatgctctt
atcaagaaaacaacgagtttgaaaagaaaatacaaactcgaaagtacaattatagtatgtgaaaatggaaaaaaaatgcactttttt
agagatcgaacgtgaaaaaatatagtgttccagaaaaaaaaaacgtttgtagaaagtgaaaattcagaaggaaaattgctgatttct
gcaaaaattagtgataccgatataaaatgccattaaattgaaaataggtttacaaaaaccgtagaaaacgaatatatacgtaggttgat
aaaaacaatgaaaaaacactgaaccaaaatttggtaattttaacataactcaaaaaaaaaataaaataaaataaaagccaaacttc
cacctaacaactcactacgacactccactttgccctttgtttaacggaaaatttggtttttattttttttatggattattcaactttttggtttttttcc
aaaaatatgtgtttcacagcgttttttttttaaattttttaagttccatttttttattagtagcagtggacctttccttggtttttgaaatacaatttttaatg
ttcccattgcatctttttgcaataatcttgtttttttaacttttacacaaacatcatatcattaatttccgtatatcttcgctacacatttccaacacac
taactctattcatctacagtaatcggattattgttccaagaattgatctcactcaagttgataaaaaaccatcgtgtgtggtctagaaaaact
gtttcctcaatttgtttctcctgcaaattccccgaatctcctttcgtttttttgtgtgcgtcggcgaaaagataatcatagttcctctcaatcttggtt
aaaaaaaaagttaaagtggttgtgtgtgtgtaaatggcagaagcacttgcgcgccttaggctccccgcgtatctgctcattgttctggatat
ctcatctcctctcttcctctcgctctctttctatcatttatttgtccgctctatggatatttgcacttcctctgaagctccgcctcatcccacccacttt
tgagaaagcgaaaaattgacagtagttatctccaggctcgcgcatcccaccgagcagcggttgtcttcgtcctcgacttacttaactaca
tcttttaccaaagacaacttttagattttatatcacaatggattacgagaacgacccaggatggaagtaccttcgccgcacccgcgagc
aaatgcttgaggtattcattttatttcttttttttttgttgaatatcacatatttttaacagtatttttcatatataatttcttatttttcaggatcaatcaaaa
ccatatgattccaagaaaaatgtctggatcccagatgccgaagagggatacctcgaggctattatcaccaaaacagctggagacaat
gtaactgtttcgcttggacaaggtaacgagaaaacaatcaaggcagatttggtccaagaaatgaatccaccaaaattcgagaaaac
cgaagacatgtccaacttgactttcttgaatgacgcttcagttttgtacaatttgaaatctcgttatgcatctatgcttatctatgtaagttttttttc
aatataattctgtcagttttctcatgtttcttaattctagacttactctggtcttttctgtgtcgtaatcaatccatacaaacgtcttccaatttacac

Development • Supplementary information

cgattcggttgcccgtatgttcatgggaaaacgacgtaccgaaatgccaccacatttgttcgccgtctctgacgaagcctacagaaaca
tgttgcaaaaccatgaaaatcaatccatgttgattaccggagaatctggagccggaaagactgaaaacaccaagaaggttatttccta
cttcgccgccgtcggtgctgctcaacaagaaactttcggtggaaagaaggctgatgctgatccaaatgtcaagaaagtcactttggaa
gatcaaatcgttcaaaccaatccagttttggaagctttcggtaacgccaagactgttcgtaacaacaattcatcgcgtttcggtaaattcat
ccgtattcatttctcgaaacaaggacgtgtcgcttcttgcgatattgaacattatcttttggaaaaatctcgtgttatccgtcaagctccagga
gagcgttgttatcacattttctaccaaatcttctccgatttccaaccaaaattgaaggaagagcttctcctcaacaaaccagtcaaagatt
actggttcattgctcaagctgaattgattattgatggaatcaacgatactgtaagtgaaaacctttagaaaatcttctgtgattttaaaatca
atatgaaatgtatttcaggaagaacatcaattgaccgatgaagctttcgacattctcaagttctcaccaagagaaaagaaggaatgtta
tcaattggtcgctgctatgatgcacatgggtaacatgaaattcaaacaacgtccacgtgaagaacaagctgaaccagatggtaccga
tggtaagttatttttcatattcagttggagaatattaaaaattttctatgatttcgaatttttacaaaccgataagtggttcattgttgactttgcgc
gtgggacagatcttttctgtttcttttctttttcgtttttctcggaaaaaatatcttctttttttatcctatcccaaagtaaatattactatatcaacagg
gataagataactaactacgtttatcccacaaaaatttgattttgataaaacatagacaatcgagaagatttgaaaatcaaatcatgttatt
acagatgctgacagagccgccaagaactacggaattgacaccgatgaattcttgaaggctttgactcgtccaagagtaaaggtcgg
agccgaatgggtcaacaagggacaaaatttggaacaagtcaactgggctatcggagctatggctaaaggtctttattcaagaatcttc
aactggctcgtcaagaaatgtaaccaaactcttgatcaaaaaggtatctcccgtgatcacttcattggtgtcctcgatattgctggtttcga
aatcttcgatgtaagaatcatattttcaaaaatttaatttttaattttgtaattttttttcagttcaactctttcgaacaattgtggatcaactttgtcaa
tgaaaaacttcaacaattcttcaaccatcacatgttcgttttggaacaagaagaatacgctcgtgaaggaattcaatgggttttcattgattt
cggtcttgatttgcaagcttgtattgagctcattgagaaggtaacaatttataaaatttatatgaaatatctctaatttgtttcagccacttggta
tcattgctatgctcgatgaagaatgtattgttccaaaagccagtgaccaaactttggctcaaaaactcatcgaccaacatcttggcaaac
atccaaacttcgaaaagccaaaaccaccaaagggaaaacaagccgaagctcacttcgccatgcggcattatgctggtacagttcgt
tacaatgttatgaactggcttgaaaagaacaaggatcctctcaatgacaccgttgtcaccgtcatgaaagcatcaaaggtcaatgctctt
ctcaacgaagtctggcaagattacaccacccaagaagaagccgccgtcgcccaaaagggagctgctggtggaaagaagaaggg
aaaatctggatcattcatgactgtttctatgctctacagagaatcattgaacaaattgatgaccatgttgaactcaactcatccacatttcat
ccgttgtatcattccaaatgaaaagaaggcctctggagttattgacgcttctttggttctcaatcaattgacatgtaatggtgtcttggaagg
aattcgtatttgtagaaagggattccctaacagaactcttcatccagattttgttcaacggtatgctattttggctgctgatgaatcactcatc
ggaaaaactgatgccaagaaaggatcagctgatatgttggctcgtttggttagagaaaagaaattggaagaagataacttccgtgttg
gtttgaccaaagttttcttcaaggctggtattgttgcccatttggaagatttgcgtgataccactcttgctagacttattactggtcttcaagctc
aatgcagatggtacttccaaattgtgagttttttatttatttatttataaatcaatcaattaatttgaaatgttctagatcgagaagaaacgtcttg
ccgaaaaagccgaagcttacaaagttatccaaagaaacatccgcagctggggagcattgcgcacctggtcgtggttcagactttacg
gaaaagtcaagccactcgtcaactctggaaagattgaggctcaatatgagaagctacaagagactgttgccacactcaaggacacc
ttggtgcaagaagaggagaagaaacgtcaactccaagagggagctgaacgtctcacaaaggagaccgccgagctcttggctcaa
ttggaagctagcaagggaagcactcgtgaagttgaggagcgtatgtctgccatgaatgagcaaaaggttgctctcgaaggaaaattg
gctgatgccaacaagaaattggaagttgaagaagctcgcgctcttgaaatcaacaagcaaaagaaacttgttgaagccgaatgtgct
gaattgaaaaagagctgtcaagatgttgatctttcattgagaaaggttgaagccgagaagaacgccaaggaacatcaaatccgcgct
cttcaagatgaaatgcgtcaagctgatgaaaacatctcaaaattgaacaaagagagaaagaatcaagaagaaaccaacaagaa
attgagcgaagacttgcaagctgctgaagagcaaaacttggctgccaacaaattgaaggccaaactcatgcaatcgcttgaagattc
tgaacaaacccaagagcgtgagaagagaaaccgcgctgatatggacaaagccaagagaaaggctgaaggagagctcaagatt
gctcaagaaactttggaagagttgaacaaatcaaagagcgatgctgaaaatgctttgagacgcaaggaaaccgaattgcacaattt
gggaatgaaattggaagacgaacaagccgccgtttccaaacttcaaaagggaattcaacaagatgaagcccgtatgaaggatcttc
aagatcaattggctgatgagaaagatgctcgtcaacgtgctgatagatcaagagctgaacaacaagctgaattcgatgagctcaatg
agcaactcgaagatcaaatccgcgccactgctgctcaagttgaacttggaaagaagaaggatgctgaactcgccaaactccgtcgt
gatttggaagaagctggacttaaattcggagagcaactcaccgttttgaagaagaagggaactgatgctatccaagaattgggagac
caagtcgaacaattgcaaaagcaaaagaaccgcatcgaaaaagagaaggctcaaatgcaacgcgaatttgatgaatcatcagct
gctcttgatcaagaagctaaggctcgtgctgaccaagaaagagttgccaagggacatgaagtcagacttctcgaacttcgtcttaagg
ctgacgaacaatctcgtcaattgcaagacttcgtttcatccaagggacgtttgaactctgacaactctgatcttgctcgtcaagtcgaaga
attggaagccaagattcaaggagctaaccgcctcaaacttcaattctccaacgaattggatcacgctaagagatcagctgaagagga
aagccgtgaacgtcaaaacctctcgaacttgtccaagaacttggctcgtgaacttgaacaactcaaggaatcaatcgaagacgaagt

Development • Supplementary information

tgccggaaagaacgaagctactcgtcaactccaaaaagccaccgtcgaactcgaacaatggagaaccaaattcgagaccgaag
gacttattggagccgatgaatttgatgaggtcaagaaacgtcaaaaccaaaagacttccgaaattcaagatgctttggatgcatgtaac
gccaagattgttgctttggagaatgctcgtagtcgcttgacaactgaagctgatgccaacagacttgaagctgaacatcacgctcaagc
tgtttcctctttggagaagaaacaaaaagctttcgacaaagtcatcgatgactggaagaagaaggttgatgacctttacctcgaattgga
cggatctcaaagagatgctcgtcaactctccggtgaagctcacaaactccgtggacaacacgatactttggctgatcaagtcgaagg
actccgtcgcgaaaacaaatcgttgagcgatgaagttcgtgaccttggagaacaactctctgaaggaggacgtgccactcacgctttg
tccaagaatctccgtcgtttggaaatggagaaggaagaacttcaacgtggactcgatgaagctgaagctgctcttgaatccgaagaa
agcaaggctctccgttgtcaaattgaagtctctcaaatccgcgccgaaatcgaaaagagaatcgccgagaaggaagaggaattcg
aaaaccaccgcaaggttcatcaacaaaccatcgacagcattcaagccactttggactccgagaccaaggccaaatccgaacttttc
cgtgtcaagaagaagttggagaccgacatcaacgaattggagatcgccctcgaccatgcgaaccgtgcgaacgaagacgcccaa
aagaatatcaggtttgtacaggcttttcttcggtgcatcatttttattttcattattttttcagacgttatttggaccaaatccgcgagttgcaaca
aaccgttgatgacgaacaaaaacgtcgtgaagaattccgtgagcatttgttggccgctgagagaaaactcgctgttgccaaacaaga
acaagaagaactcatcgttaaactcgaagctgtaagaactttttgaatgacttttcaaatcaaattctaatttttccagatcgagagagctc
gccgtgttgttgaagcttcagtcaaggagcatcaagagcaaaacaacgagctcaacgctcaaaacgttgcctacgctgccgccaag
agtcaactcgacaacgaaattgcactcctcaagagcgatatcgctgaagctcatgctgaattgtccgcctccgaagaccgtggacgtc
gcgctgctgccgatgccgccaagtgagttgttttactcacagtaacccgtatcatcatttttttttccaggctcgctgaagatctccgccacg
agcaagagcaatctcaacaactcgaaagattcaagaaacaactcgagagctccgtcaaggtaaacagataagccctaactttatct
ctcaactttttttgtgtattacaggatcttcaagagcgcgctgacgctgccgaagctgcggtgatgaagggaggagccaaggctatccaa
aaggccgaacaacgcctcaagtctcttcaaaccgatttggaaaccgaaacccgccgcgctggagaagctaccaaaacattggctc
gcgctgaccgcaaggttcgcgaattcgaattccaagtttctgaagacaagaagaattatgataaattgcaagaattagtcgaaaaact
caccgccaaactcaaactccaaaagaaacaacttgatgaagccgtaagtttattacttatttttcttgattgattcgaaatacaattgtgga
attttttaggaagaacaagccaacaaccatttgagcaaataccgatccgttcaattgtccttggaaaccgctgaagaaagagccgattc
cgctgaacaatgcctcgtccgaatccgaagtcgaacgcgcgccaacaacgagcaaaagtagatatttccgtactttcataatcccctc
cccctttctccccccacataatttttgtcgtcgtccctcatatcaaaagcaaatgtcctccccccacattttatcccccaccataaacgtgtc
ggttgaaataagtcatcttctctgatttcctaaataaaaaaccactttcaaaaaaaaaatgataatgaatccccccattttaggtgtagtattc
tcttgttgttggacaaatgttccttctgattattgattgattgaagttaataaaagcatcatcttggctttccaagtgtgtgttattttattatgcattc
a

Can-pop-1

partial coding DNA sequence: join(3134..3701,3756..3905)

ctctattttcatgccatttttttgtgatgcctgctagatagctgcttgggttgtgtcttcttttctctattcgtcttgtcttctattttttctgttctgtttttttgaa
tattttattttattttcatttaccttgttcttcataaatttaataataggctttttgtttcatataggtagtaattagaattgttttgaaatttaattataact
attattagcgaatgtgacgttttcggcttcaccctttggtgtaaaataaataataagagaaattatttttggcgccaaagaatgtcaggtgg
cgtttttatgagaagaagcaacattttgtctgtcagatatgtttaataacacacacaacatttaaataacatttttgaattgaaaaaactttttt
atttcatttattttgtttataaaaaaataaacaaaaaatattttgaaaacattatttggaaacagttcgctgcgagactcaaacgagtgaatgtgt
gctcctttaaaataccctactgtcattatttattttattttttttttgcaaaatctcaattccacccatatttcgcagaatttcgagagattttcaagac
taacgtttcagaaaaatctaaaactcatcattttcgtaaaacattctcattttgtacctacatcttcctctacaacctcatttttcatccagttttttt
ttcttcgttcattttctctattttttttcttgttctcatcattgattcatttttttccctcattcgaacgtacgttatcagctcttcaaaacgttcagtttttttttcc
ataaatttatatatggttttcattttcgccccattgcacttcttctccgttttcttattttttatgacgccttcccccacccgccaatgacacccaaa
aacctgttactttttttggggtggctgttctggcgtctttttttttcgggtggtgaaagttgttgttggtttttctaggtcgccttgttgttttgtttctatctt
atttttgaactatttatttattactcatgcccgtttttcatctgatttttgattaaatgattgaaattaagttcaatttttgcaggcctttaaacacaaa
aaaataaatgcaataatgatggccgacgatgaaaatggtgatgaggtgaaggtgtttagacgtggagaagatgtggatgatgataca
atgatcggagaaacatccgaacaacaattagctgatgataaaaaagaagcagttttagaagcagagttagaagttggtaagttgttttt
tggggatttggagaaaaattgggagaatttgaggctttctgaatcggtatcagagtagcctaatttttgtgcacatcgggcttttttttgaatttt
cgaaaacaattctgactcgagtctcatctttcgaaatttgtcaaaaaattaaaaaatttcgttcaacaaaaattgaatttttcaaaagaga
agcataatgtaacctgacacctcggctgaggtcgcggttttcaaaattgaatttttactctaaacgtttaagaccgttagaaccatgcctag

Development • Supplementary information

atagtctatttctcaagaagccaggcctgaaaagcttttttaactttgaatgttatccatcctgattctggtagctttttcctcggaatttattca
aaaattctacagtaaccctcatctcttccattcccattagctttttctctttgccatctccaaaaataaaataaacaaattaccacaaaaaat
gtatttatttacattaaacctaattaattgccaatcttaaacttttctttcgcaccttcttcttcccaatcaaaaaaaaaaaaataacgagccac
atgtcaaaaaatattacaatttttgccgtatccaataaaaataaataaatagaaaaaagtttttgtggagttcaagagcggaaaattgcg
caattctcatttactaaccagacatgtacgaaaagagataattataaaaattgcgcaactttttttctacttgtatactactactgtttgttaatc
cgaggattaaaaaaagaggagacatatggaattgaatcaagattttcgtttttcaggttcaaaatcaggaacaagcgctttcatcaaac
ccgaaccttctccatcttttgccagtttagcaggtttaatgccaggaggtccattcagtccaggatatgcttttccaatgtttatgccgcttatt
atggtgagttctttattttcgaaaaattcaaaaaaatacaacaatttccttttgtcttcaaaatggtaatagtcgggtccggaagcaattagc
acccacccattcattggctctcttttgtctttctttcgacgacacacccttgacacttttgtttttttgaaaaaaacatgacaaaaaagggaatt
gtatcggtaacattatttgggggagaaagagacagtaattacgattcgatcgagacataatttgagtaggagaaaacggagcaagac
ctacattttttgggggcgagagagaaaatgaacaataattggatgatgatgatttttggagaatttggagagagggggtttcaaattatc
gtagaaacttggaatggttttgaggggagaatttcgaaccctgattttcagccaaattaaatattagagtataattttcagcttctattcttga
acacaaaattcaagctctctaatcgtcatagagtctattctttgacgtaaccttttttcctcgtcaagttttaaaattcaaatttcatttctcaaaa
ctcgtgaaaatctttctgatcactgatcatcaaaatcataacacaaatagggcgaaagaatgatttcaagttcgaaaaggtatgagttgc
tcagctcaattgcgaatcagcaccaaaacaaatagcattcttcgcagatgctcttgcacaatccgtgtctagataatatacatagattcgt
gtaaataaacagagattactgtaggggcgtaatgtttaagcaaatataatgggagataaccaatgggagattgtgaagaagaaaata
gatattatgtaattgggaatttttgaattcagctcgggcatacctcattcagaaatctgtaaaaatctgttttatcctcaacagcctcttattttc
agcctcaatatggattacgagccggactttcaccgaattttgttctcccaatgcaagctggtctttcaccttcatttaatatgtttccaacatct
ccattttatggtgctgcaatggctgcagttgctaaacaacatatggaaaatgcagttccattagcagcaagtatgagaggaacaccatt
gaatccgttgaatcaaatgagaatgccaccgtatctaaatccaggaaatggacatttacatcaaaaagaaagaggacatggcggag
gtggaaaaatcaaaaaagatgatcatatcaaaaaaccattgaatgcatttatgtggtttatgaaagagaatcggaaaaaaattgctgga
tgaaattggaaataatgagaaacaatcggctgaattgaacaaagaattgggcaaaagatggcatgatttacccaaagaagaacag
caaaaatattttgaattggcgaaaaaagatcgagaagatcataaacaaaaatatccacaatggtcggctcgtgaaaattatgcagtta
ataagaagaataagaaaaagaggagggataagagtattagtaagttgtttttgttagaaaatgaaaatatatttgtatttttgtagtttcag
tttcggagaatggtgatcagaagaaatgtcgagccagatttggtgtgaataatcaagaaatgtggtgtaaattctgtaagagaaagaa
gaagtgcgaatattcgagtgatagaaatacagatacaatgatgatggatttacaagatagaggtaaggaatttttgaaaatttttgtggg
attcaagaaatggactgggcatttttgtggatttttaggtttagagaatcaaataaagatgtttccgaatattttaaaacccgaaaattcgg
attttttgaaatgtttaagactttgaaaatccacgttgctaagtttttgaaaattatttggttgaaaaatcaacataccaaagtttctagatctttt
ttaattcaaaaaaattgaaaaatttgtttgacttgaaaaatcctcaaaatgaaatgctttccgaattttacatctcacgaagttcacctgatct
tgaaatgttccccatttctattaattttgaaatccaacatttttcattttaacatactttcaggatcacttggcccatctccattagcctcttccata
ggtcgatcaccaatgggtgcaaatgcttctgattcagaatctgatgtcgaagatgaagatgtcgatccaacaatttcacaacaagcaa
gagaagtgattatgcaagaatcactatgtactttataagaaaatctcgaagaaaacaatcaataatcagttcaaatccgggtgtttttca
cttctctcaatttttctaatttttcatttattatatcaaaattaatttatctttttcacctcttcctatacctttttttgctcaccaatttcgctgaatttgaa
acaggtgctcccaattctttctctcttttttttttgattttttctgatttcacactatctttttggtttttttagggtatcttgtgagtttaggtaataaaat
gaatgaccaaagaaattgtgtttcttttttgtatatatagtttgcggttctttttggaccacaatctcttttttgctctttttgatgtatcttttctcttat
ttatattatgatgaaaattttattttttaaattcagaagatgatttggtagacaattattatgccaaaatttttggcattaaagtttttcaaaata
aattttagacgcccaaaaatctacaataatctggattagattcaaacatttttttatttcgaaaatcagccagaaaaaattgaaatta

Can-skn-1

partial coding DNA sequence:
join(2362..2763,4598..>4928,5401..>5544,5941..>6205,6774..>6863,6945..7296)

agtagattttcagaaatgaaataattagatttttatttcttacgttcaaacgtgacccatttttatctttcgaaaaaatctgaaattaatttatttct
tattttcatcaattcgaaattctcgatttttctaaaatgccaatcaaaagatcaattctgtgagtttcaatatcgtaattttcacgataaaaacta
ttaattcttcgatttcttgagcattttctgcgtacacttgtccctttatcgcctcgtgatttttatcgaaaaaatcaataatctggcgccatttttcct
cgcaatttcattttcaatttcgaattcttatcttttttcttcaaaattcttgcatgtttacccccgacctttttgtagataatttttgcccaaaaaat
ccgtttttcttttaaaaaaaaacgatgtaaacacacatttttgcgcagcaaacatcaattttgttcgttctcttttgtttcgttgatctttttcgctatt

Development • Supplementary information

ctcttcaatttttcttctgtcacagaccacgaatttgccgcaactctgtttttcatctgcgtctctccatctgattttctcgccgggtcaaaatttc
ccttcttttttttccaactttttctctatttcctatttgtcatcatttttcctgagccaggtttttccagaaaaaaaaaacaatttttctcacggctgc
tatggagattttttttattgatttgttgcaattttattttcgataaccttgaattaaatttttaagtgtttttgtttagagaaacaaaaataaattagta
aaaatctttggccaagtttcatttaatattgtttttctccgcgtctctcgatttctagttttttttgctcgttgcccttttcttatcaggacaagtatggttt
tttggcaaaatgcttatcgttttgtaaataaatttctgggtttctcgattttcaatgatctctaattcttgagttggaatttctccgcgtctcgggttc
cccacaatctctaactttctgattttctgattgagtgagttttatcaacgcaaatgcaattttctgcgtctctgcctctaaattatctttggtcgtct
agatattcaaaatagaagtgcagtagagcaactttgaaaatttcgagaaaaaactgtatgctatcttcccgtgttctttttttctctccacttat
ctttatctaccgttttcctgttttttggatgatgcaaattgtctgttttactaatgagaaaagtgcaaacacgctccaatcacaatttctcgtttttct
ctgcgtctctctctcaccttcttccatttccacgttttgatctatgaaaaagagcttatcaaatgttggaaaaaagcctttcatacaaatagat
aactttttaaaattctcccatataaaccgttttttttttcgcggcaacacaattaatttttctgacaaaagtcacgttcgttatcgaaagaccgt
attgatgtatgatgtcacggatagctattttcttcttctttttttctctctttttttcttttctctgtagtacttttgtagaatgacagacacacattgaga
aaatagtctttcacaattgttcgttcttcttcttcaaaaaacatatatttgtttttgatcagactagactctagttttatttttcagtcaatcctcatct
ggctcgtcgtttcttcgccattcgtcacccaaccagaatttcgacatttttggcggaacgagcgatttttgagcgaagtgttgatcggattg
cggaaattacggaaagaggtatatttttttggattttgaaaattttatgagtttggatgaagaacagtatttgtgacaataaattaaatattgct
taaatattttcataagttattttttttccgaagagttcaaaaatttttaaatttcagttctcagactttcttgaaaattttaagatatttttttgtaatttg
agaagtgttcatgctgctcatttttttcgaacaaaaacaatgtttctatattatctcattgaacttttcaaaggttcttgtgttttagccaagttagg
gatttttataagcctaaatctaggcctaactcagaacagaatgtagtcatcaaacccctaaatttgagcctcgaaaatttttagtttctctg
aaattccaaaaaatcacgttatcatcatccaactatacgaattttcgaaaaaaccaattaatagtgcaaatttattttcatccaacctgcgc
aaaataaaaaaaaataattactcatgtatcacatgcacttttaattatcaccttccaactcacgaaaaacaggaatcctttgggaatatgat
tttttgcagcacacaaaatgttctcgcaatcaaataatcaggaaggatattcggattatcaacaaaatccaaatcaatattcaaatgatc
ccaaaatgacgccaggtttctatcgtccaatgggaattcgttggagagatgatcaaagtttgatgcaagttcaaactatgccaattttaac
tgaacacagatcagcctcgccatttggaacaagaagatcaaattcaccatcaagaccaaccacttcgaatcgttttgatacatattcat
atgattcgccatctttggaagatgctgatttgattgatgttttatggagaagtgatattgcgattgaaaaaggtgttagacaagttgcaccgg
ctgaacaatatgaaagagatttgcaaatgttgactgagaaatcaactattactgtaggttttttttcaaaagttggattttggggtttaaaaat
gatatgggttattgaaaaaattatccattcaacaaaaaaatcaaggcataggcaattcgcacacaaaaaaattgtggcaacatttctctac
atccataatcgaaccaagttcctcgttgttcttgatgaaaagccatataaacacaatcgaccaatggctcatttggttgagtcgaatccca
attcaaataatcagcttgtgttccatcggaccatttgtagacttgatttgtgtcatttagcttattcaaaccaatccaaatgttgttttctgtaaat
gaaattcgaaaattgcgatgaagaaattggccaaagttcgaaacaattttgttcgaaattgggctaattttttgaaaagcgggattatag
aaattcaacgatcaaaatgagtctctaaacaacccaatatttttaaaattaaaaaccgttccaaacaaaaaatttggtcctttttttccctgtgt
caaatttttttttaataccaactttttcgcaatttgtgaataaatagattttctgattcatccttgatactcgccaaatgtcctcccatacttctacaa
aagttttctgctggaatccacatcaaatcaacttcgaacagtttgtaccaatggtcttcatacatttgccaaccctgcaaaaatgtttcctttc
ttgatttcttggatttgtggtgaatcataaatgtacttttccatcattggatgattttacgaaatgccgaaggatgattttcaattaggatgttttga
actattggaaagtgcatgtgatactctcgtcagaagctcaaccaattactaaaattaggagttatgagtagtcaaaatcacgagacctct
ccagagacaatctatactttttttcaaagttctcacgtaatctttcatcggacaaagaactccacaaaaagcacattgtatttttatcagtttttta
ttcggaaaaatgatgctatcttttcgtgtattgaactattggtaaaggaaaagtcgtaaagtctgatggagaaaaaaatgaggaaaaata
atattgagtcatttttgggtcaacgccttttgataaaaaaaaaagtaacatgcgtcagaaaaggattatacacagtgaaatagattcagag
attggactgttttaatggctacgatgggactgaaaaaaggattggagctagaatcgggatatcggaaatcttctaaacttctgtaaaactt
gggggagaatcgcggaattttatctctgattttctatagatgattttcttttttgattttgaactcgcccatatatcaaacggttatcctaagattg
atcttcgatgactcgacgaatgatttttcattcgaccatcaagtcttcttatttttcctataaactcggtcttagtttgagccttaaccttttcaagc
ctacaattggaatgttctcatcgtttagtgatttattttgtttcatcccaatcgaaaaaatcagtgtcatttcatttcattttatcattttcgcctatatt
cccactttctaccaaccaactttgtctaacttacactttgttgcaatgctaatactgtcaataatcgaacattcatatcgagatttattgcgcg
agttcctgcgattgaaactggcacatctttgagaggcgatggtatcactactgaatagattcttgaaatcaacagtaaaaatgttagaaa
cagtttcatgatttgtttagaaacaactaattttgtcgaaagggcgccaaactttatagggaaagtatcagatttcggaaaaaaattataaa
caatttatattttccagggtttgacagctgaagagcatcaaagatatgaagatttgtcgaaaggaatgtttcaagatttttatccaagttttgc
atcttcacaaaataacaacaataattatcaaaatacccacaataataatttgcaaatgaaaacattggaaaatcgagttgcaccaattc
caagcgatgaagatttggctgcattattggaagatgtttcaaaagaagaatcacaattgaatttggtatttgatcaacaaaatattgtgca
gacacaacaatcacagcagccaattttgcaaaatgtttcattgagcgagggaattgtttattcgcaaaacaattgtgagttggatttttaa

Development • Supplementary information

aaagatatctgaaacatttccgaaattgtaaactaaaatttcaaagcccccaaatattctttcaaccaaattaatagattcaaaaattttcc
taagacctgagaatcataaagttaatttccactaatgagatctgtcacttgtgaatagttacattattagaatcttttctcgaaacatcatttttg
tgctacaatttgccctcttctatcttcccaccctcataagcctcataataatcatcgcccaaaggtcttcccacttttctcctttttaaaaacag
cacgtttcgttcattttcctgattcctgatatacgatttatcagttacgaaaatgcacagtttctccgactgaacgataaaactttttcgttcga
acgtcactttttttgtcatcatcaaaaatcttatcttttttgggttttccccttttttattttcctgtttacagtaaccgaaatgagcatgttacgaagt
gatgaattggcaccaactgcttccaatgaagatttgcaagctgagacgtttttcaattcgactgattcgaatacaatgcaacaatatttgat
gccggctgaagctgaacctgttgatggtatgtttcaagatttgaatagtttttaattgtccgattcaaacagccgaaaaattagatcatcttg
gtcccattcaaaatccaaaaacgcccgccatttttgcctaaaagttagctgacctcgagatcaaacctataattatcgctttatccgagct
ccgcccatttcagctctaaattttaattagctccgcccatttttcgcgcacctgacacatctcctcaagggattttgttgctataaaaggagtt
gtactatagtgaaaagtacacacacttctaagcttgagcttctctcgccaactccaacttgaatcttcaaaaatgaactatttcaacactcc
ctatgatttttcggcggcttttgccaacttccaccaactggatttttcagtcttcccaccatcgcaattctcctatattccaatgatcaacgaca
ctgcgcacgaacaagtttcgaatggaaatgttgattttgatcatagatattcgagaattccaccaaccgcaacagcaacagcaccaac
aagaccagctaatcatgatcaacttggagctgcgatttcaagtgcattgggtggagctgcaagaaatggaacaacactttcaagtcca
ctttttgatccatatcattcgatgagacaatcgttttctgatgtgtaagttttttttaaaattaaccaaggtcccagaaaaataggcaaaaaa
caagagatatcaatattatttctagaaatcttcttgatctacccaaaaatattctagttttgactattccatttgtaattcgtgactaaactagac
agaaatatcatccatgtttttcttcctcctaatgtcatcatttatttcgaaatcgttttcattttaattttcacatctacttagatattgtcagtccgata
tttttcagccacttggaagagaaaagccgacggcctagaaaaccaaatttttctctttcaatacaaaatatcagagcgctctcgagcag
ccgcaaagtctactgtattttggttttctcttccacggctcttttattttcggattgacaatacctttttttcctatttttcggctctgttttgaaaatgga
caaccaaagtcctatggaatttattaagcgaattgttgagaatttttgtaaagttgaagagaagaaaacatggtaggccctatcataaaa
tcattcgactgaaatcaaataaactattgatttttgcagtgatacgtcatcgacttgttcaagattgtcatcggaatcaccaagatataatag
agaggaaggaacgagtcaaggaattccagctagcaggtaagaaatagagttatgacgtggcaaattgacactttgctggaattacg
aaattagccaacaaatcaattgatttttcagattctatggaaaattgattccaaatgatttcactttcggtcgtcaaacttcgccaattcgga
aaatctcacgtgtcattccattgactggacaacaacaaagaaaacgtggaagacaatcaaaagatgaacaacttgcaactgaaatg
gcacttccagtcactgctcatcaaataagcgaaatgagcctttccgaattgcaacaagtactcaaaatcgacacattgtccgaaggtc
aaaaacaattgatccgcaaaattcgtcgccgcggaaagaacaaggttgccgcgcggacttgccgtcaaagaagaactgatcgaca
tggaagagccattgatatgaattatatttaaagtttttattggttctccttactttttaccctattatatacctcgaaagggtctcgtaatttttaatt
atttaattcatgtgaatttatatttgtctccccgtgtttttaaaggcctggagcctttcttttctccccatactatttttttagaaacgggtagagctcc
gttggactctttttttatacccgaatgatctgtatttgttatgagtttttacaagttttcgaatatcccatttctcccccccagtgaaaatgtacacat
gtttatacccaatcaatctcccctaaagtttttttttctattgcgatttgatttttgatttttaatgattttttttggaaaaataaagtttgaaaaatgtata
taagtgagagaataattatggttgcatttcttcgtcgatttgcggcggtggagcacaacagcaaggtttacgattgaagagggatttttttga
cgaaatgagaaatacgaccagattcgagctcatcgccaatggctttggcatccgtgacaatctgaataaaaattgagtctggtcgagt
ctggtgtttcagttttcaattgttgaaaaaaaatcaaactcattatgaaaatgagaattttcagtacatgttaaaaatatgaacaatttttagat
aaaattttctaacttttcacgtcgacatcgaaactataattggaaaatattacgaaatgttcaat

Partial Can-SKN-1 coding region:

Kmfsqsnnqegysdyqqnpnqysndpkmtpgfyrpmgirwrddqslmqvqtmpiltehrsaspfgtrrsnspsrpttsnrfdtysy
dspsledadlidvlwrsdiaiekgvrqvapaeqyerdlqmltekstitgltaeehqryedlskgmfqdfypsfassqnnnnnyqnthnn
nlqmktlenrvapipsdedlaalledvskeesqlnlvfdqqnivqtqqsqqpilqnvslsegivysqnnltemsmlrsdelaptasnedl
qaetffnstdsntmqqylmpaeaepvdvfppsqfsyipmindtaheqvsngnvdfdhrysripptatataptrpanhdqlgaaissal
ggaarngttlssplfdpyhsmrqsfsdvdtsstcsrlssesprynreegtsqgipasrfygklipndftfgrqtspirkisrvipltgqqqrkrg
rqskdeqlatemalpvtahqisemslselqqvlkidtlsegqkqlirkirrrgknkvaartcrqrrtdrhgraidmnyi

Only a single SKN-1 orthologue is apparent in C. angaria. A search of the partial Can-SKN-1 protein sequence to C. elegans returns Cel-skn-1 with an expected value of 1E-109.

Cpo-elt-2

Development • Supplementary information

join(5001..5043,6093..>6148,8819..>8900,8953..>8982,9039..>9128,9187..>9328,9384..>9438,
9823..>9908,9961..>10129,10212..>10309,10902..>10985,11050..>11153,11235..>11455,1155
4..11631)

ttgtcatcttgatgaaaaaacaattcccaaaaatcccaaatgaagctgatgagcggaaatgatgaaggctcaaaatgagtctaatttg
aagattcgggtgctaagattttcaattgaaaaaagagaaacaccgtgtttagcttcaggggatgtgagagaaaacaacacatttagg
ttgtgacgtttttataagaaaagttcagaatgttattcatgcaacctggaaaatgaagtaagtctctattttcagagaaaaaatgtggaaa
actttggaatgcaaaaaaaaggaaattacggacagttaaactatataggtcttgtctgctagatatgtttattagagtttcggtgacatgac
catattttgttccctagtaaacatttagaattgaaatgggtgtatcagactcgctaaaaatgtttctgtcagtttttatattcatatttgtaaatgttt
tttcatttcattttgtttttttttgtaattatatgaatagcactgttgagaaatgagacattcccggaataaaccgtgccactttgggagaacttca
ctaccccgagaactgaaaacattctcgaaccgagttgttgtttcacgattacatgaccataaaaaattgaaaatcatttttcgaaattaat
aactcgtttttgatgacatgtcttcagtttcccttcagaatcttttacacagtttctgacaaattttttcaatgagaaaaaataaaaatcattcat
cataaacagtattactgtcagttcatgatcgtactctcaacattgtattcattgtatgagttttacagagtatatttcataaccaatcagataatt
cattcagtttcacatacaagtatatattttgatgaacatacactcaaatcgcgtttaatatcagaaattgacagcttatcaataaatctccatt
aaaattcatccaaagtctaccaaaaccgatattatccacatgtttccatgcgaactaactaagaagattttcaatttgcgtcaaagtcgga
taatgatcctactagtgtgatgccttctgtggaatgcatgctcattccgagtttctgacttgatagaagatggtcgacatcagatcgcttgct
gctccaaaagtagccattgctgtggctggagttctagttctggtcgtagtcactaccttggtggttgtattttatgtcaggtatgaacaaaaa
aaatgaatttcagaaagccatgcatttcaggaatcctgatgtgactgatgcaattttcaacccgggaaaaactgaaggaacaacttca
gcgaccatctcaatccagggagatatcgactctggtagttctttcgtcacaacggtttgtgatagcacttgaactttttttgttcaactcaggt
tcttttcgaaaattttccagactgtgagctcatcgggaagttcttcatcgtcattttcttcaaatcaacatccagctgatgctattaacagccgt
tcgtaaatctcattttgctaaatttcatttcaagaaaaacctcttcattataacatgaaaaaattttcaattgaaagtttttgagagagttttaca
acagtattgaataaatatttgatattgatgttcaatctgataaactgttcattaaaaagtgaaaattttcatcgtaatattcacagtaattttttg
atatagtgaaaattataaccaggttgccggagatatcaaaagctaatagatttttgtctaatggtatggaatataaccgaacaccctctta
tctacttgatcttataaaactttaattggaagaaatatggtcactgattcacgatagaaattgtttctgccagtttccagagaattttcgagttg
aacaataaaatcaaagttttgaattgaatacgttctagttagaaaatgagaaactaacataagttggaacgttttggactgataatatcgt
tttaaaaaaatgttgttgaaatgacatagttgtggaaaagtgtaacacaaaaaaaatcacatccagtttttcgattaattcaaaaatttgtg
agaaccactcttcgattcgtttatagtctaatcaagatttcagcgttttggttccggaaaaacaactgcacagggaattgcacgactatctt
ctgtgtgcattcaatgagtgtcaaaaaacattagtttgaaaaaaacttttataattttttttgctttctctactgcttgactacgccgtgaaactc
ttgttactccaaaaacccagaaaaaaacatccaagaaaaacctttctcatcagatttattatactctattgttgtgcatagaatattcacca
aatattatgtcatacaaccaatccgtcttcattcccatcatgtcacaaatgatatcattccagagacaactccgcgagccgacaatgcga
agactacaacagtgacaccggctccacaacaattcacaacattactacatgccattgaaccagttgagcacgtataagacaaaccat
gataatttcaaaattatgacagaagaatctttgagtttataaacttgctcactttgaaaaaacatctcatcgtaactcacatcgacttacca
aaaaaataattttctaacaattttcattttctttttaatatcttaaatagactataagtaattcaccataaaattcgaaatattttgcacttaataa
acacattttattgacctggaaacaacaaaattgttatgatggcaatagaactgataaacaaataaactgaggcaatgaagatatttgaa
tgttgttttcgataactcgtgactattattttcaagagaaaaaattatctacagtgagcggtcacttctcataaatatctaacgagttataata
aaattagccattgatgaaaaaaagacaatcacatcataagcagacagctttttcgagttcgagtaatgttagttttgaaacgaattggga
aagttggtaatttcaatttgagtgaacatgtttaattcattatgtttcgaaacctgaaagttttgaaccatttttttaacagttatttttgagcccaa
ctagaaattgtttttataataaataaaaaaagacaggtttcaattcatttttgtccatttttaagtagttttgggacaattcagaccacccatgc
agaaaactacccacattacgttttttttaattttcatgtttttaaaatgtaaatatccaagacaaattgaaaattaccacaaaattgaggtat
gatacttgatactgtatttaactttcctgttttcaaattaaaagttcacccgggcctgtggtaaattagaggtggtaggcttaaacctggacc
aggccgagaaatattaagtatgagtcattttcaaacttcagcaaaaaatcccaaatatcaaccaatgactccttattattttccgccagct
aactgtttcatatcgggaaaaatggcagaaacaatttgagaagaattgaaaattttcaaaatttccaagtctctaaaaatttatgaaatga
tgattttagtcggtttttcacccagaatgatattttttgtagaaaatgttacaacttggctgaaatcatgaaaagtttttcaagacacttcaacc
aaaaatataaacgttcatattatcactctacagacctcggtatttcgtcatttttttgaatttctctccgggctgactcgaaccctatttctgaaa
attttcgaaacaacttctacgatgttcaagttttttttcagaaatgtcactctttttcagacacttactgtaaaaaacaattttaagcattctaatc
ggtttttgttcgaaaacttactcacatcagcgtactttgtagataaaatctattatcaatatggaaaaaaaaatgataaaattggttttttatttt
atcacaaaattgcaaaatccgctgaaaattcatattttttcatggagcgttctgcaattggaaaacatcaaacttcgaaatcgaaaattaa

Development • Supplementary information

ccactctatcagagtatctctgttaaacgtaaccctgaaactaaactttcgagaacaaatatggaaataagggatccaatcaaaattt
caaaaacccaaactttttcatgtcaacccctattagttttccaacatatattttgtcaagaggggcacctcccaatacttccctttccttttcca
ccactcgtctactttttctgtctaccatttgtcctcaattaacaggatgattccatacaccgaagtaatcaatactccttatcaatgttggtgtg
ttgagcggtgtatatatgtagttttgagaaaaagtggattgccgaaactaagtgcttgaatatggttgaaatatcattgacatggttcgagtt
cagaaaaacggaggcaacttcggcgctggtgggaatcagtcattatgaaatttgagagaaaaacgcatggaatcaagaaacattgt
ttcacttcaaaatttcaactcagaaacacattaaaattataaatagaagagcttttgaaaaaaataagaacagtgggataatcatgatttt
tcatcacagttcggtttggaacacgcctcatgtttttgatcgaaatgaactaactaggtaaatgttttggatctggattttagggtttcctaaaa
aggaatatgtaggtagctataagtctaaacaactgtcacaattgtttttcgaagaactgtcagttgaatttcagaactcacaatcgtaaaa
atgtgaaaatcttttttgtgtgtttttgagacgtctgtgtacagtgtagggagtgatttggaccaataattttctgaaggaactccataccttca
gcaagttttcaaaccatcattgactagaattgagcttcaactaaaaaatgacaccaatttgaaagcagatagattcacgaagtttacac
cgagatattgaaatactcaagaatacgtttatcacaactttccgacaatgttttctggcggttttcagaaaactatggccaggattatttgga
agaaaagcacaactaaaaacgcgggtgagtttttcatgaaaaaaagagatccctaaccaaaaacaattttgttgaaaccttcggaaa
ctagcgtttttgaaaacaaaattttgaagtttggacttgtttttttaatactattattctgcgttcacaaaaaatcagaaaccaaaacctgaa
aaaaatcgagaaatgactggcaccacaactacaaacttcatgtagtgacgggaaaaaatccagacactttcgcaatttaatatttgaa
attttcttccctatttgtcagaaatgatgacgtacacactgatttgggtgtcaaagtcgatctttaaaagtttttaactgacattaacatgattttt
caatcacaaccaaagccttcatgagattcctaaaacgttaatttttacataatttaatactaccagcaaaagttgaatttgatttactttgatt
cagtgtcaataaatctctcgttttttgtaattatatagtttttcgaaaaattttgaatcaaaaatttgaaattcacaatgaaaaacaaagaaa
gtgtgttttcaatactactatcaacaaaaaagtgttagaaactcaatatcgcaactttttcaacgtttatattgaaaaagataatgttatctgt
aattgatatattcttccatctgaaccatagtgatatcaaatcatcctgaacgataaacacagaatagtgataaaaataagcattttcaatttt
tcacgcctctcctctaacccttgtatattcagacttctttcagatggatttttggttttgaaacttttcacccccatattcaaccttataatagctaa
tatatgcgatgtattaatgatggctgtattatatttgtgtataatgttgggctgaattgtcgaatagagcagtgatgtgcacatttcattgccgtc
atttactgataaggatacagtacccggaatattgaatatagagtcgcggccttattacaacaagtggaactcaaggttctaccatcattttt
tgtgcaaactcggatatttgtcttgggtacatttcaccatccagaaagcgaagaattttcaaatttattccgaggaagtacagataatttttg
acaaaggtgagttgctatttgaaatatcgaatataagattgaattaatttttgacctacgtatcacaaatgatgttaaatgtgctgaaaaa
agattaaaatttgaaaaattaaaacgagttcgagaaaaataataaaacaatcaaatcatcggttgattacgttatcaaaaattgtctag
ataggtgagaaaaacgtttcgaaaactttttcagttttaaaagtaagaaaaccaccgaataaccaaataaaagaaaccattatgagtc
tctgcgagtcttgtctgctgggtcaagtttcgatttgcagaaagtttgattcttcgaaaaccttttttgttcgaaatttttcatcaaactaaattag
aaactttaagtcatcaattcacgaaattccgtattccggtttttatgaattactgtatttccgctcgaaaataatgagaattttcccaaaatcg
atagatcaagaaaataatcaatgaagcttacacaacacactttggcaacgaatgaacagttcgagttgcttgctaacaaaatttgctgc
tgtaaaaacttcaacaagagttacaactgttttatacttttaaaacacaaaaaacgtgatggaaggaaagaaatcgttgattttctcatga
gaaaacgtattgagatgcttgaaaaaccattttcacaagtcacttgtgattatttaacaacagtgtcttgtcgatttcaaaataatggtcttcg
tattaaattttctgtatcactttatttttcgatgtctaacaacgatgtcagagcacctttttccaaaattctgacaatgattttacccacaaagtt
gatcttaatcaaaacaaaaataaattaagacaagaaagattatttttatttttctgtataggtcggaaatgatgaattgataacttccctcaa
tagttttgggtgtagttttttttctggttatcttgaaaccttcacaatcaaacttatgagcatagttatcaaactcgattatcaattcagaagcca
cttatttcagtaatgttttgataatttttcagaattaaactgactcttataaccctttgaattttcgtatatctttttccatccggttttgattcgtgata
aaccaaaatcaaaaaaccgtgactcatgttaaaactatttaatttttgaaaacgcttattttcaaccgtactctctcgatgggaggagttgg
aacacaaaactggcgaatcagaaaacgtcgtttttaaccatccagtcgagtcccgttacttaccttcaacagtaagaagggcggtgat
aagataagacgtgagcgtgtgagtgtgtgagcgagcacataattcttcgacatactggctcgtctattagttttcaaatcatttttacctttcc
atctacccttacaatctcctatttttgcttgataaaaattatatctacttttcttcgggtttcttatcgtttcacacgtttttttcctgctaccgctgtttttt
tgtgatatctcaaaaaaacttatctataaactgatgaaaacatataaaaaacgtctaacaatgattaggaagaaataagggagttcatt
aaaaaaaagattaatggttcaaagttttattgaatttaggaaaggacttctgtggaatactccttttatgaactgaagaatagggaacaat
atttttaaaagtttgataaaacaacttgacatgagaaatgttatgaaatcactgcatagggaaattatgttattactaaaaaaatcgataat
gactataatatttcaaggaagttcaaaattcagaaagtttttgttcgagattcaattcattgaactagattcagtttaacgtataaattttctttta
tgttagtactagaacaggcgtggttctgggttcagttgataggaacatcaattttcataaggaactctggttatcatggaccaatacaaca
aatgtaattctactacatgagactacatgtattcccgacaaaacccaaatttttcttagatcagaaaaattgaagtgtcagttgaattgaga
aaataataagaaatagaatgcgactgaaactgattcaaaatatccattattggcatttttgtcgtgacccattcatcttaccacgttgtatttt
cgaaacgattttgggttcttatgtgactttttgtcaaaagacttttcgatttcacataatctttattatcggttcttgactaatctgacaaaacggc

Development • Supplementary information

cttacttcttaggtgtccgaactgttggttttggtaagtggaacgcttggtgtattgtgaaaactgtgtacttctcctggattttaaatttgcaag
aaaacttcattatttcgatgtcaaattggccgttccatctgcaaaatttaatccatttcgtcggatttgtcagaaaacgataataaaattttttg
aaaaaagcctatctggacccatagggacgcttgagccctactgcggtcatcgagaaccgctttagccaatgagtgttcagttagaatc
acactgaatttcatgaactgtatcgaaaaaattcaatcattgacacaaaacatcatttatactgaaccacaccgagatgagttcaaatta
ccgatttctttgggaatgttgtccgactttttacaatttatctccaataaactgaattttcaggaaagaaatggatggtcaacaatatatggat
ggagtttggccagaaatgcgccaaaatcagctccaactgccaactcaaccggtacatcccacttctcaacacatttcaatcaaagaa
gaggatgttccaaaagttagtagaggaatgcaagaactgacaaatagtgagttacgaagactttaaaaatgtttttgtattgatgaaattt
acaaatttcagtgaaaatagatgatagttcaattgacagccgccagagtgtgatcacaaatcaaactgggattgattttaaatcagatttt
caaatgacccgtaagaattttcccttttcaaaaaattggtgttttagttgacttatttgaacttccagctgctcattattttggagggccattgga
ctaccaacagttcccgggttttattgacaattcaatttttcaaacacctaacccttactacaatccagtgaatcctgcccagctcagctcgtt
gagcggatttcctggtagctcgtaagttggaacacctttgtaaatttaatgaaaaatttgtgagctttcattacagatcgttgtatgatcggcc
aatgtcttatgatgcaaggactgcgttcccacaagttgtgagtgtaagttcttcccacgaagaactttttttcgagtagccaatgattttcgaa
cgagaccattttcatctgaaaatacaagaacaaaaaacctatgctttttttggaactcaatggcgactaatcgtttcatagtggacctaatc
agttctatgaagccagttctatgaagccgagatgatcagttctgtgaaatggttcagaactggtttttttcgcacttttcaatcgacaaattgat
gatcccttttttccctaatcacatgttctgactagggtttcaacaacgtttttttcacataaaatcagacttgttcaaaaaatttggtctgacatg
aggttgcgaaaaaaaacacatttcaaattcatactaacattttgcagaatataacgtcccaatatgctgcttcttatgaatgcctgaaatgt
ggaaacctttgtggtttgaactataaaaatgtgaatggagggtaaaaattgaaccgaaactctcacaaaatcttctcaactctcaattttc
agacaaatttgtgataactgtagtaaagaagtttacgaacaacctacatcacatatttatgctactccagctccgccaatcattgaaaca
caacctgcagttgaacaacagccatcacttccagcaccggcaaaagccaccaagtctggtaataagaaaaatggcggatcggtac
gcaatgctttatcaccaaaatccggggtttgaaacacagacctaaatttcgaaattgttggtgaatttttagttttagggtaattcatcaaga
cgtcaaggattggtgtgttcaaattgtaacggaacaaatacaactctgtggaggagaaacgcagaaggagaaccggtgtgcaagta
tgtatttggtatctttcgaaaaatagggaattcacatggaattctccagtttgtgagggttgtcttagagtttttagaaaatttaaagcaggtca
gcaggttttattccgcagttgagctgtcgttaagccaacctacttgaatgtatcagctgacccactgaacgtaattttcgctctgagcaggtt
agctgcaacctatcaaattatgatttttttgccatttagcagattagcttaatcaaagcggatagcctgctgaaaccaaaccaactgaacc
gaaccacaaccgaaaccacaattcagctgatttgaacgaaaccagctaaactgctttgaataacccgagtttcgctagaaataattct
ctatgttggtttcgaaaatctggttaatcagtatattcagattgaaaatctgacgtatttttttttccttcagtcaactttctctgaaagttttgatgt
gaagtatatatttgagacaccggtattcttctgactggaactttgaaattcttgaaatactggaacagaaatttttcttacctatcaaaattttc
aagaatcaatttttttgttttttttcagtgcatgtggtctttactacaagctgcatcacattgccaggccaacttcgatgaagaaagaaggtca
attgcaaactagaaaaaggtgagactccgttctaacattgtttttaaattttttggtgaacagcgaatttttaaattttaggaaaacgaataa
gagtgaaacacactcaactccaacaagttcccgcacaaaagaacggaagtttgatggatctactgctgcagacagagccgctgcta
gaaatggtgagtataagaagtaagaaaaaatagcttgctggaaacttcagaatgaaaaattaaaaaatttaatttgtgtttttcagcttc
atctatcacctacactaccccggctgcgtatccggtcagtagcgggcttcaagaaacaccatactacacttaccaacaagcatcgtgg
aatgcagttatgggatcgtcgcaagacgctcatattagcttaccacatttgggcctagatccagcttcccaaacaactgtctactcaactc
attcttacacaactccagttttcgtctcaaaaccagatgtaagtttagtggaaagaaattaagagagaaaaaaacgagcgacacacta
tttctctgtttcaaagctctgtttcataattttatttgaaaattacagcttcaagtcataccggttcaagacgatgaaacaaaagcggcggca
agagatttggaagacactacagacgaagtataagcttttaatgaatacactttagttgaatcacaaaaaaaatatttattgcatttttatgttt
cgagtttttatcagtcatattcatattgaaaattgtttttgtatacacggatattttggatttttcttttctcatttttgtaccaatcagtcggctgtaa
agtttttttttgatgataacttacgattttgcaatttcagaacttgttttaacgtacttagtcattattatcgttttctgacaaatccaatcaaatggc
gtaatttttgcaacttcacaactgctagtcatgaaaaggggaacagttggtacgacgttaaaatgatataattctcttgcaaactcaaaat
ccaggagaattggtcagtttttacagtataccgagcgtccctcacatcaagacgaacagtacggttccccccctaaagttaggccatttg
gccggatttgtcagtaaacgatagtgatataatataagaattgaattgaaaaaaatac

Cpo-elt-3

join(1604..1682,9347..>9535,10540..>10645,10985..>11108,12491..>12667,13144..>13238,13
296..13440)

gataagctttacaaattcgtgaagtttataaactaactaacgttttctagttttgttcaatgggtattttcctctatcagatcaactatttgaacgg
aaaagaaatagttataatatcccatatttttttattttcgaacataaaatcccgaaaatgccctagttccaaaatttgttgaagtttttgagta
gtttggtttttcgattctcgacatacagtaccggccataaagatacttgtagctctgcgaggtttcaaccaaccaaattcaactgattttatttc
ttaaaacttgagaagtaggcttctgagaacataagaaacgtgcttcaaattttccatattaatttaatcacttttcacagagctacaggaaa
aaaacacgtttttatttttcgtgtctttatgaccttatctaatttcgaatgatgttcaaccagttttcttgaaaactgtatcacaaaaccgtcctat
taacagtagatcacaaacttgtgcattatgatgaaaaaaaaatttggtgtttcactttcctactgtagtacaactttaaaaattattcattttttca
cagggcacactttttaagtacagtttttttcaaacaaaaaaactactcaaaaaaccatttattcaaaaatatccaaaatacggttcggcaa
aataaaagaaatcttgtactcgtacttgccgaaagttgaagcatgtcaaaagttgagctaaacgagacagccgcactttcccagaggt
gactttcaccaaagaagcaccaaataagctgcccctgaccaccaagcggcgaactttcaaaatacatagtgttaacatgctctcgcc
atggcggtattgtttttgaactaatcaactcgccgccaatacaatacaacaaagaacatgggtgtgggtttgggcggagccaactacc
gagcaacattatcagtagtataagtagacaatgagagaacacccaaactatcataattagtcaccatgatatgagttcgtggtggtttca
tggtgtatctatcggttttcttttattaatgagttatagaatgtaaaatttttttcattattttatattattgactttaattttcttcaaaattatgaataaga
ctgtacaaacaaggttgttttcagattttgagttaaattctgttggtttggttggttgccatcccttgttcaaataatgaaaatatacatttgaata
tgaaaatctaaagtaaaacaaaacaaaaaatttctactcatcatattagagtagaaaaattttgtttctcaaccaactttgttttcgtttttgttttt
tcataattttgattgaacaaagtattattgattcattcagtcggtgttatttttatattgtatttctcacctgttttttttctgattctttaatcttctaggttct
tagggtaatttttcctttttttttaagtcttggtgacagcttgacgttttaatcaaaatttaatcttttctttccatatttaacacttttatatagaattgaa
cgttttttttctttgttaactcacccaaaaagatgttcaacttttcagagcagtgataattattaataacataattaataaagatgaacagcttct
actcgacatccccacaatctacagaatccgattcttcgcagcaaaccaactacaatgactggaatggtaattttttttcttctctttgggtcag
atagtatttgaaaaaaaatcaaaaaaaatacaacaaccgtgggaaaaagaaaatagatttgatatctaatcgaattgtgtatgtagaattg
gtctgtgggggggggggggttatcatataaaaatgaacgaaggaatgattattttccaaagttcaaacggttgaagcgatagttgagat
attttttgaaattcctgggggttttggggttcaaaaattttttttcgaagaaaaaaaatttcatcaaaaaaaatttttttttgaaaaaaaaaattttctt
ccgaaaaaatatttttttttaagacattaaaatttttgccagttactattattgattcttctcaaacaaacatgttaaaaattttgattctagaaca
tttccaaaaattgatcgcctccaactataacgtttttttttcaaaaaatctttttgttagttattgtttgatcaatatttgatttacatttagacattaca
attttgagacgatgatcgattagttaaaaaattagttgccccttcttttcatcatcgtctctttcatcgtcaaaaaattctactttttcatgttccatg
ttttttttgttagtgtcgcattctcttcttccaccccgtcagcatactcatcgtccaatttttgtttattttgatttttcttatttcatttttatattatttttaaa
aaaattgagaagtttaccttgttcagagtttcgatgaaaaaatcatatttttactgtttttgtattaattttgtccacattgatttttatagcttgact
aatttgcttcaaaacgaaatttttcatcgaaaaatgataatttctgaactttttttccataaacagtttacaattttgttgatcagccaaattttgtc
gaacaagcgatttcgttgaacagaagattttgttgaacagacaattatattgaaaaaaaaaatttgttgaacagccgatttttttgattatcc
aacttccaaacaacttttctgaatagatcggtttttccatgagcagcgtagattttaggttgaacagcagatttttttctcgaagtattctaaa
aaattctataaataacgttggaaattttttttgtcgaaaaaaaatttaaattttgttttggaaaccgttttagagcaatgtattttgtgaacaataa
gattttcgttgaaaatagcggcaaaactttctaataacaatgtcaaaaatcagttttttttcgaaaaacggctatattgtcaaattttttgatc
gaaaaaaagactgtttgagaaattctgttcaatcaaatcagctgttcagctgaattcaaccgttttgaaaaaattttgtgaatagttcaaaa
aaattgtgaatattataaaattgccaggaatgcgactcgtagaagtgattgaattatccatggtgcatttacatttttgtgtcgaagaaatta
aacctttttgatttttttagtaaagttttactgatcgtgatttcattcaaaaatgtcgagtttgaagtttcatataaaaaaattcagtttaagaaaa
acagaaaattaagaaacgtgcttcaacgaattcatgatttcagactacctacaaatttttgatatgttggttttcaacgatttcataatattgg
tattcacctgaaatagctaattttctttcggggtttttcttaaaattgatgtttaaaattgatcgattttgtatggatattcatgcttgaaaacctag
cagaattttgtttctattagcggcgactgaacagcctacattgaacttgagtgagttgaaaatgctattctcttccgtcattttgaggcgttcta
gtcatgcttattaggtttttgaatatgctgatctgtatgaagtcggccgcgttcgaaaagctcgtgtcgacttgaagtgttttccttattacaact
tccgaactcaaattatcaaaatttctcactctccaaacacaattctatatccttatcatcattattctcttcccccatttgttcattcaagatacgt
tataatatcaaacaatttgaattgctgctagcttttctagaagatttacgagccactgatgcttcatcgtaaaacttcttcgttctcctttctccct
tttctcttgaattggatcaatctgttgctgctgaggctgacccttgaaaaattacggtagacaatacgacacgcaaaaggcatgcagag
ggaaaaaggcgcagaacgttttggtttggattacaacaattctcgcgcttctactacgtggtcactactatcttttcaggggagaactatttt
tgagactgtctgctttattaatattcccttgcaacggttgagaagtgtcccggaaaataactgaagaaatgaatacaactactagagggtt
agttgtcagtcgaaagcaacccaatacattgtttcacggtcattttcttgcgatttgactttctagcgcatccgttttctccatcataaattatca
ctagcaagcaatttccaccccccatagggtcctatctttttccttctctatcacaactttttcatcagcatcagcagcatcagagtcgggaatg
tatgttgacgtcttcatctccatcttcgccttagttttcgccttcaacttctacgcagcgcagcgcgcgatcgcaaaatgcaaaacgcaaa
aaagtacagtactgactgcggcgagaatagagaccacgatcaaccacaatttgtatttgtaacccgtctgttcgatccttcccactacat

ccatccatctcctatccatctatccgtctatccatctatccgtccatccgtccatccgtccaagggacagcgacagaacgacgcctgcgg
cggggaccgatgtctttttctttttctttttccttttccatttccatttttctctgttcgttccaaccaatccgagtagaaacggtattaggaaaagg
aagaagggaaaaagaaagagcgcgtaatattactgtatgtctctggagaagggctgatatttctcaaatcactgcatcactttcgctcc
acgatcgaccccggttaccgaataagtctcgattgttctttcctccccaccccatgattctgatggtgtgcgcgttagtatctcgtttgaatcg
tttcttttgtgataataagatattctatcaactttcgaattgtttccctcctcattcatccggacgatgaccaattaacgtttgcagcctagatagt
attctcgaaaaagttactgataactattattttgattttgaaataatgaagtggtgttaataaaggatactgttaatcaatactactaaggttct
cgagcagagtccctaatttgatatttttttcagtttcggaaagtgacattttcgaactaaagattcattttacttcaaatcttacaagtggggtgtt
ttgttaggggtcagaaattgatctctccttgaaaattcactcgagagtaggtatttcttgatccaaatggttttgattttatttctgggagttttcg
gcacctgtttaccaagttatacaggccctcaggctctcaaacaccaaaaagcagtttttttgggtatttttgccacggaagagacaattttttt
gagattctgacatagtctgaacataaattcaaaacgctgaaaaaatatgagtcgtttttccagttgcagaattattccgttggtatacaattc
acctcaagactctgaaattttggattgactggataggttttcgaatctcttgattttgccctctataattacaattccatgactgcaaaattgaa
attctgtctcaatagccaagatgacacgtggaaatcaatttccaaaatccacggaccattcgaacgcgtctgtgaaacgaaaaatttca
aaattttcagtttgactgttatagggactgagtacaagaaaaacgttcttgttagatattctacaagtagaaaaattttttttgaaatttgctcc
gaggatagtttgtcttggaatcccaccaagctaaaacttgggaacgagtggcttttcaaaatcaaattaagtagagatgaaaccacca
aaaaccatagatcgtttttttcagtatcaaaacctcgagaaacaaaaaaaaaacttgctctaggccttcagattctttaaaaaagcaggat
tttatttaaaaatcaatttctgtcagtaatacgcagtaaaagctatggattactataatctttcctttcgttgtttggcttcctttctctattttctaac
ccacaccttgattacttttcagttttattcattttcattatctcaattgaacagattttttcccaacaatcttaggcagtttttttgatctactttgccat
tataggtgctttattgcattatctgtcctcatcatttcctcaaatttcagtcactagaaaccacaattctgacccctctgaccttttaaaatcaga
aattcatttccatgatccattgaactacattcactcttatcaatttgataagcatttgttaaatgttatcattctttcacgaacgctactgattttta
acgctttttgaaatcttctgatttcgatttgaaaataatctgataaaactttgtttgcggtgtttatttatttacaactcttggttcaaatcgtttcaat
catttttgttgtttttcaaacaataagatagaaaaagcagaagcaggagcagcaaacaatacatttttttgaaggaaaaaaagaaacgtt
tgtaatattttgtattcaataaaaaaacgagatcagagacccaaagttctcacgatttgaatctgaatggaagctttttttccgtttccttacctt
tccgctactcgagagtggcggaaaaccgtaccgattatgaatatgtaaattatgtaaatttcgtgtttgaaccagctcgaaagtttttcttca
cctcaattctatccaacttttgtcattttttttactttttactttctactttctcaattggctatcactttccatttttcaatttctgtttttttcttctttttcaaagt
tgcgacactaccaccaccacaacattatgcaataaaagcagtaatgatagtgaagtaaaatgtagttgttaaaaagaatcaaaaaat
ttatatattttctattttctattttctattttccattttcttcaacagtgtatttgaaaaaagcgtctccaccagactagccagtctataagtctcggtc
ctctgcggccattgatggcgaaaaaggaaaaagatgggcgggcgcgaccactcttgcataccgtagtattactgcgaatcacgggg
cattgtctttttttgtctattcaacgtttttttgcgcctatcggctatcatctccccctcacgcacgccgcaatcaaataccacataccaaaaat
gtaacactactcactcataaaatcattattattaatcaagagtattttttttcgtcaaagtttttggtttttttcgcgcatttttttctttttttgattgatcca
tcgattttcaaagatcttccgaaattcctcaatttttttgacttcagaatcagaatcttgtgataagataaggccaaaaaaccacgttagata
aaattaaggaaatgggggggaggaggagatcaaaacacacccaaaatacaaagtttccaaaaacaaactaacacagccgtaaca
agggaccttccagagttctaacaaatagtttgaactaatatttctaaactgtggaatgaaaaacgccacatagcatcacaggcttttaaa
aacgggaaaaaacacattttgaccgtaattttacgaattttcgaacattttccagtaagaatgagctgggcttgtttcattattttttttctgata
gagcacacttaggactatttgaataaaaggattaggtattcacaaaaacagtgacctacagccaaaaacagctcagtctcgtttatgtg
attataccacctcaacaaagtgaatgtgtttttttgaatataccgaatcccataaatttttaataattgacacccccagtgtgctttaccagga
aagaatgaccagttagatcgaatctttttgagagaagtttgacaatttgtcaaagagagtcataaacgcgttgctctcggaatttttttttca
ctctagttttttcacgatcatatgaactactttggtaaaatctttgatttcaaattttttgtctaacttctgaaaccttccttgtgagaacttttagca
gcttgagaccagctaacttgctgaatagggtcaaattacctgctgaagggtcagaagataagctttctcaattcagtaactaactttctga
ataccccaacttattgaaaactttgaagtatctgattagcttagaactgacgacactcttgctaaaattatttttcagaatctacagatgttttg
tcgattagacagaaaatcttccatttcaacagagtaacaaaaacgcgatttcaaaaggtttttcgaaagtttgaaaatcagatttgttttttttt
ttaagaatctgtcgaattgtgaaaacctctgttgacttgacttttgtaaggataatctgttgaattatgatattttctgaaaacccgctaaaac
aagacagctcactttttgattctgaaagtgactttttttagcagattagccgacctttcaggttagataatcaatttcatcaatatacctgctga
acgtcacaggaattcagcaggttatcaacgagttagctgtctaatgtaaggtcagatagttagttgacaaccaattagcttattgaaaact
cgctgagactagtctaatgaactgttgactttgaaaatgacattctgtggatcagttgacccttcccagcaagttttcaaactcaattcaattt
aacaagttacctgaacaacttctgctaacccactgtaatccatcagcttaccttctaaaaacccgctaaaacacgattctcgagttccat
aactttgaaaaagttctcctttgttgaaaaaaaggttacgatgtacaatataaacagaacatgcaaaaatggaagggcaaagcttggttg
aaaaatagaaagtatgagaaaggagtcactgtgtacacatatcaaatatgtggggaagaagggttttgccatccgatttgatgttttaca

Development • Supplementary information

aaaattccaaagtttctttatgtatgtgtgtgtgtgtttttcttttttttggtaaccttctggtaataaaaaaaaagtgttgtttgacatgagcttcc
ccatctatttatgaatctttctatatttgggttttctgttatttttcatttttatttcaccttttcactctactctgccaatacttcgactattcaattcacaa
taagattgctcatcattcttgatacttgacggttgttagcacacctcctcactacttttatgagtatgcgcatattgaaagtttgcattcccgcc
gcttttctgttttactttgtagttttgttgcaatttcatgcctatggttggaaaaaaccgagaaaaaattttattgccattcaaaaaaatttcatgtt
cacatcaatattgaaatttatatttttgcggtttttataactcgatgaatcattctgatttttccaggttacccccagcagtactgtggttataca
acaaattcggatcagcaagcttattactgtcaaacagctccgaatttgcatttatcgactacatcgcagaacaacactgataactcagct
gctccattgccagcgatttccaacttcatgaaggaactccaagttaaagatgattacaacgactattatagtgagtttttatgatttttttgcct
aatttcgagcggtttgtgttattcaaaaccgaaaaaaattttcccgaaacttttattatctaaatttcatggatattgttaaaaaacctgttttttt
ctggttgaaaaaattttctcgaaaattataattctcgatttttcctaacagtgacaaatttgattatcaatctagcttcccagtttttgttatttcac
caagctgacacgtggcaaaatttttcatccagttttccaggcgaaaagttcagaagccacaaacttttgttaattcaaatttcaaaaaaa
agtttttcgccaagaaatctagaaaaaaaaatattcagcttgggtgatacgtttcaaaaaaaatttttttctcaaaaaaaacatgatttttc
acttcaacttaacagagtttaccatttagagattttattccttcgttttaagctcttttcatcaaagctgccacgtggcaatttttttatttttcagttt
ttgacttgattggttaactcatgaaaacccgttctaatatcgattttcgaaaaaattcgaaattgaaatttccgtatttttctcaactttttgaag
attttctaaatttttcgaattctttatattttcaaaatatttagaaattagtcagtttctaggtttttcatattcttcccgaattttccaaaaaaaacca
aatcacaatcttattttgtttcttctcaactttttttgtaaatagtaatgtttatagttacaatataatcataataataataataataataatgtc
gcccaccctattaaagacagatgaccttttaccacctcttcaaccaaccgcactgtttctgatctttatcttccgatttctcaaccgcattttatt
tattcataccagaagaaagaaatctatcataacgtgttcattagttttgattcgatgttatgttacaaataaaaaaatttttaaaaaactgattg
ttccgtttcagcagctggacaacaaatgagcgactatcgcgttgaaaaagtcgtcaatccacttgctgatccgtacactcaattggaac
aaccaatttatacggaatttaccaacacgtgagtttttcttcttttttttcaaaatcttattttctgataatagtcaaagttcaatctatgtatgtatg
taattgtcaatttttcattctctctctaaaattcttcaaatctcgaacaaaacgttcatgactatcaaggtcagttgaaataaaatgagaaaa
aaaatgaaatgacaattatcaatcaatcattttgttctatcagtcaatcagacacttttcgaaagaagaataaaacgatattttatttagaa
aaaaaagtaaataagacggaacgaaaagactttcagacataaaaaaaaacttttcctaaaagtatgatttcaagaattttcagaccact
cccaagccaacaagacatgatgaaccacatgaacttctcaaattccttatccgcaaactatatgacagtaatgccagtaacacaacct
tcagctgtgccgctgaatctttttgatttcgtgagtttttttatttgtttttttagtgtgaaaagggtgtttttggtattatatgttcaccccgaaaaaaa
ccttcccagaggccggcgtcttggtctcgtttagactgccatgcgagtaagtattgagtcctgcggagacgctccctgcgtctccgcagg
ggtcagtacttactcggatgcagtccaaacgagaccaaacagccggcctctgggaaggtttttttcgaggtgaacaaataatagaaa
aatgacaaaaaaataaaagaaaaaaacactagttgtgtttctgaactattctaactaactgaactatcagacgaacgtgctactgcact
cttgagacgtatttttgagtgaaatcgagaaaattcaaaaaatcctaaaaacccatatttttattgaaaaagactgatataaatcaagtct
cgtaaccagaacttcacaaattcagagttttctcttgttttacttgagttacctgattagtttcaactgaaaatctcccaaaatgacagtttttg
atattttcccagtaatcttcacaaggaaggtttttttatgtcaccaatagaatttttctgatttttacactgttttttccaaaaactagacgaagta
ccaattcggatattttgtggatctttggttaagaccgatgcatttatttttggaaaagttgaacaaatatttgaaacattatagtctttaaaattta
tcaatcgcccgaaaagtctacaattctcaattatctactaaaccagacgcaattacagcttgaactttattggattgatttttgagagttttgt
atcaaaaaattattcagaatacgtattgatccgaaaaatttctcacaatcagtgccaaaaatacccaaaaacgctattttcagtttttggat
cactactctcgaggaatatctgaataacaaaacgtattcgcccgaaacaaaacgcccgaacagcatcaaatagattttcgtaaaaaa
aaaccaccaaaaaatcagattgtttcttattctgaaccacttttctcaaaaattagctggcagttgttcatcaaaaactcatggtcaacatta
caatcactttcgaaaatagtctttcgagctagtgagccaacttctgctcgagtttttcaaaactgtagttacaagactgaaaatgaaactct
gatatttccaggagccatttttgggatataggtcagattttcaaccttttcagtcgaactctaattttttaagtgtaaaaaaaataataattacc
atagaaattcaacgaaatctgtttcttttcaagttcttttgaaaacgatcaaaaaattgaattagacaatatctacttcattcgattcgttttttta
atatctcctttttttcagaacctctcagcattcccttccttcaacacatcagtttgcgagccaacagcacttccactgatgcatcaatcgccttc
tcaacaaacctacgtaatggctgcacattcaacattcacaccaccaccacaagatccattagtaccggagcaaaagccagcagca
aagaaaagaatggcggtatgttaatttgatctctaggtttcacaaaaattgccacaaaaaacttcgaagaagactgatttttgtgaacca
gcatgctcggaaacctagaaaatcgtcaaatttaattgcgattgaccacaagtttcacaacaaatcacagtagtactgtaaagcccca
aaatataaaaaatcgtacaaaaatgacacttttgttttaactgtaaaaagttcagtcgcaaccaatagattttttccagttttcaaacatac
cggtttgaatttagaattatttccatatcgaaatgataattagaaaggaaaatgataaaatttattttgaaatgagaacctagacgcagtc
gtttcgaccccaaagttggacgactaggtaacgggtctcatcagcaacattaatataccggtttgaataaaatccatctcgttcaaaaac
tcgaagttttagaaatttcaatagttttcttgtttacaggctgtccaatgccatcaaaactctatttgctcaaactgcaaaactcgcgaaacg
acactatggcgcaggaatggagaaggaggtgtagagtgcaagtaagttactaaattaaaaaaatttcattttcttctttcaacaaaacat

Development • Supplementary information

cttttcagcgcttgcaacttgtatttccgtaaaaacaaccgcaaacgaccactctcattgcgaaaggatggcatcatgaagagaaatcg
tcgcccacgaaaccaatcgccaaatggtggcatcagaagacaaggacatgctgcagcttgctagttcaaagcgagcaacttcacta
taattttttcgttttttttctgttcaaaatttgatatcttataacccatattgacagttcaatggtgtttgttgacactgtatacacaaaatgatgac
atgaattgaatcagttttgattgtaattttcattttcattttcattcatatcacaagctcgaatggtgtcagatagtcatgagagagggtcaact
gaaacccgaaataaccgaataaccaaactttctcaaattttatgtgatttttctatatattctttccgtgtattatcttccctttcgattctgaaga
aaaagtctagttattcaataactcttgccattttcatactgatcttggattacctaccaaaaatctttattcacttgcgaatttttccaatatatcat
atcttctgtttattgattttttttcgatcatgtttatactattagtccttttttttttgtttttctttgccaattgtcatcgttcaaatgaccctctctttttcaa
ccaaccttttagtaccaacgtaaatttttcaccatgactcgaaactcgaaatgctcttcttgttccctcctgaaatattttgtgacaaccaatttt
ggttcattttgttggatatttactggtattcaaagttctacagtttctattgttctcttattttttcctatatgttttgttttttttttctttcccattttttatttttat
cataattttctgtcattatcattttcatcataatcataggcataacaacttgtataccatgcacacaataaaatataaaattttcactagctcg
gaaattagaaacaatgaaaaaaatgtgacaaagttgatgaccatgacaaaaaatatgaaaaaaaactgctttttgttatcaattgga
gattggttaagagaacaaggtcatttttgcgatgactgaaaaatgacgaattgcgtctacagaagaagtgcaaaaatgatcgatgaat
ggaaaatatctccaaaaactgggtttcgaacacgattgaaaaaaactgagagaattcattg

C. monodelphis elt-2, *elt-3.1* and *elt-3.2* are described in Eurmsirilerd and Maduro, 2020.

2) <u>RNAi clone information</u>

Sequences used for RNAi constructs (feeding and injection, where applicable): DNA was amplified from genomic PS1010 DNA, or synthesized directly, and cloned as HindIII / SacI fragments into the same sites of pPD129.36.

Can-skn-1 (smaller 520bp region from genomic DNA, contains a small intron):

gatacgtcatcgacttgttcaagattgtcatcggaatcaccaagatataatagagaggaaggaacgagtcaaggaattccagctagc
aggtaagaaatagagttatgacgtggcaaattgacactttgctggaattacgaaattagccaacaaatcaattgatttttcagattctatg
gaaaattgattccaaatgatttcactttcggtcgtcaaacttcgccaattcggaaaatctcacgtgtcattccattgactggacaacaaca
aagaaaacgtggaagacaatcaaaagatgaacaacttgcaactgaaatggcacttccagtcactgctcatcaaataagcgaaatg
agcctttccgaattgcaacaagtactcaaaatcgacacattgtccgaaggtcaaaaacaattgatccgcaaaattcgtcgccgcgga
aagaacaaggttgccgcgcggacttgccgtcaaagaagaactgatcgacatggaagagccattgatatgaattatattt

Can-skn-1 (larger fragment, 750bp, synthesized cDNA):

aaaatgttctcgcaatcaaataatcaggaaggatattcggattatcaacaaaatccaaatcaatattcaaatgatcccaaaatgacgc
caggtttctatcgtccaatgggaattcgttggagagatgatcaaagtttgatgcaagttcaaactatgccaattttaactgaacacagatc
agcctcgccatttggaacaagaagatcaaattcaccatcaagaccaaccacttcgaatcgttttgatacatattcatatgattcgccatct
ttggaagatgctgatttgattgatgttttatggagaagtgatattgcgattgaaaaaggtgttagacaagttgcaccggctgaacaatatg
aaagagatttgcaaatgttgactgagaaatcaactattactggtttgacagctgaagagcatcaaagatatgaagatttgtcgaaagga
atgtttcaagatttttatccaagttttgcatcttcacaaaataacaacaataattatcaaaatacccacaataataatttgcaaatgaaaac
attggaaaatcgagttgcaccaattccaagcgatgaagatttggctgcattattggaagatgtttcaaaagaagaatcacaattgaattt
ggtatttgatcaacaaaatattgtgcagacacaacaatcacagcagccaattttgcaaaatgtttcattgagcgagggaattgtttattcg
caaaacaatttaaccgaaatgagcatgttacgaagtgatgaattggcaccaactgcttccaatgaagatttgcaagctgagacgtttttc
aattcgactgattcgaatacaatgcaacaatatttgatgccggctgaagctgaacctgttgatgtcttcccaccatcgcaattctcctatatt
ccaatgatcaacgacactgcgcacgaacaagtttcgaatggaaatgttgattttgatcatagatattcgagaattccaccaaccgcaac
agcaacagcaccaacaagaccagctaatcatgatcaacttggagctgcgatttcaagtgcattgggtggagctgcaagaaatggaa

Development • Supplementary information

caacactttcaagtccactttttgatccatatcattcgatgagacaatcgttttctgatgttgatacgtcatcgacttgttcaagattgtcatcgg
aatcaccaagatataatagagaggaaggaacgagtcaaggaattccagctagcagattctatggaaaattgattccaaatgatttca
ctttcggtcgtcaaacttcgccaattcggaaaatctcacgtgtcattccattgactggacaacaacaaagaaaacgtggaagacaatc
aaaagatgaacaacttgcaactgaaatggcacttccagtcactgctcatcaaataagcgaaatgagcctttccgaattgcaacaagta
ctcaaaatcgacacattgtccgaaggtcaaaaacaattgatccgcaaaattcgtcgccgcggaaagaacaaggttgccgcgcgga
cttgccgtcaaagaagaactgatcgacatggaagagccattgatatgaattatatttaaagtttttattggttctccttactttttaccctattat
atacctcgaaagggtct

Can-pop-1 (773bp from genomic, contains a small intron):

cctcaatatggattacgagccggactttcaccgaattttgttctcccaatgcaagctggtctttcaccttcatttaatatgtttccaacatctcc
attttatggtgctgcaatggctgcagttgctaaacaacatatggaaaatgcagttccattagcagcaagtatgagaggaacaccattga
atccgttgaatcaaatgagaatgccaccgtatctaaatccaggaaatggacatttacatcaaaaagaaagaggacatggcggaggt
ggaaaaatcaaaaaagatgatcatatcaaaaaaccattgaatgcatttatgtggtttatgaaagagaatcggaaaaaattgctggatg
aaattggaaataatgagaaacaatcggctgaattgaacaaagaattgggcaaaagatggcatgatttacccaaagaagaacagca
aaaatattttgaattggcgaaaaaagatcgagaagatcataaacaaaaatatccacaatggtcggctcgtgaaaattatgcagttaat
aagaagaataagaaaaagaggagggataagagtattagtaagttgtttttgttagaaaatgaaaatatatttgtatttttgtagtttcagttt
cggagaatggtgatcagaagaaatgtcgagccagatttggtgtgaataatcaagaaatgtggtgtaaattctgtaagagaaagaaga
agtgcgaatattcgagtgatagaaatacagatacaatgatgatggatttacaagatagagg

Partial Can-POP-1 protein sequence:

Pqyglraglspnfvlpmqaglspsfnmfptspfygaamaavakqhmenavplaasmrgtplnplnqmrmppylnpgnghlhqk
erghggggkikkddhikkplnafmwfmkenrkklldeignnekqsaelnkelgkrwhdlpkeeqqkyfelakkdredhkqkypqw
sarenyavnkknkkkrrdksiisengdqkkcrarfgvnnqemwckfckrkkkceyssdrntdtmmmdlqdr

Only a single POP-1 orthologue is apparent in C. angaria. A search of the partial Can-POP-1 protein sequence to C. elegans returns Cel-pop-1 with an expected value of 3E-64.

Can-elt-2 (no DBD) (522 bp genomic fragment, contains small intron):

aactttcgcaactggaacaaatcaagattatttgtataacacttcttcaatttttaacacatattcaactcaaccgcctccatcacaatatca
tactttatcatcatcgtaagttggccgggaaattttgaattcttgaatgtttctgatttcacaataagaaaatattttcaagacatcaaactcca
attttttaacagattttacgataattcggcatttataccacaacaacctttcgcgccagcaaatccagaatgtgtcaaatgcgcaaatcca
gtcatttccggaagacaagttgatggtggatatatgtgtgatacttgtagtaattctaatgtatatgatttgtcacgaattcaaacaaactatt
cgatgccactttctcttgttcaaccaattgaacctgctccaattgaacaaattccacctccagcaccagcaccatcaaaaccaccaaaa
tcatcaagtaacaaaaaagctggatcagctggaagcacaaatcgccgacaaggattggtt

Can-elt-3 (no DBD) (270 bp fragment, common to short and long isoforms):

ccgatgctcaatcaacaagatatgctaaacccgatgaatttctccaccaactacatgggagccacacaattctcccaccaacagact
gtgcaactcaatttgctagatctcagcggcttctccacattcaactcgtgtgatccagctctaccattaatgcaccagtcaccttctcaaca
aaattatgtgatgccacattcaacttttacaccacctccacaagatccattggtcgctgaacaaaaaccacttgtcaaaaagcgaatgg
ct

3) <u>Plasmid maps and cloning information</u>

Development • Supplementary information

All fragments were amplified by PCR using Q5 polymerase (New England Biolabs). Plasmids were cloned by Gibson Assembly into pBluescript KS- digested with BamHI/HinDIII. Fragment sizes include primer overhangs for Gibson Assembly. Linearized plasmid maps and primer sequences are shown below.

pGB598: *Can-elt-2::ELT-2::GFP*

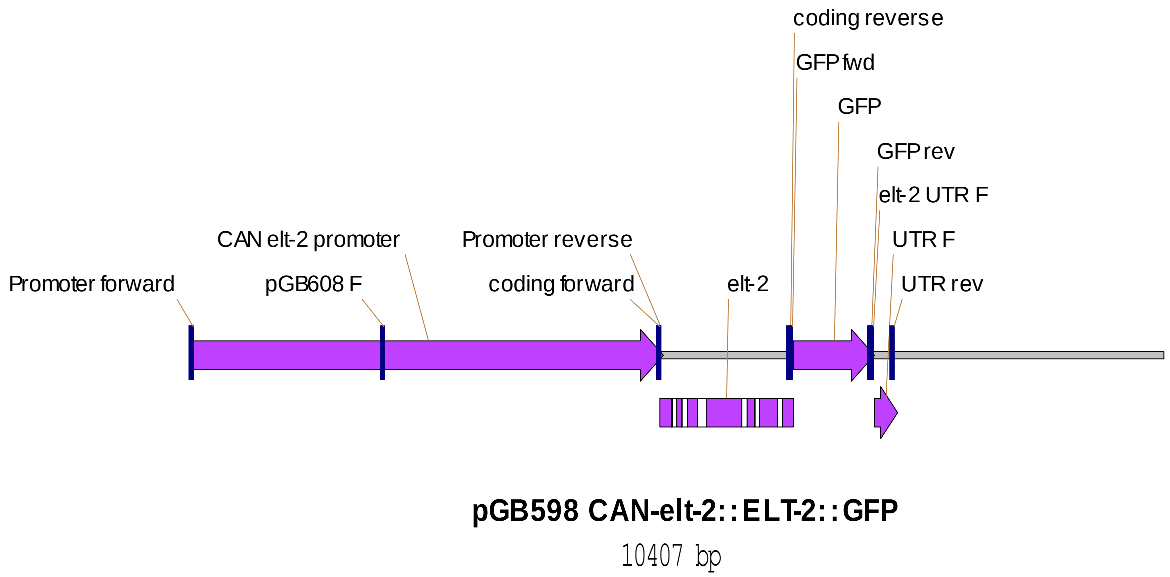

<u>*Can-elt-2* promoter (5040 bp amplified from PS1010 genomic DNA)</u>

Promoter forward, ctatagggcgaattggagcttcatttattatatgtcattacggaaaaccg
Promoter reverse, gacgcatactatcgatccagggactctcgatcg

<u>*Can-elt-2* coding (1436 bp amplified from PS1010 genomic DNA)</u>

Coding forward, ctggatcgatagtatgcgtccaccaacattgcaact
Coding reverse, ctttactcatttgttcctcttccagatctctcgcag

<u>GFP (870bp amplified from pPD95.67, introns not shown above for simplicity)</u>

GFP_fwd agaggaacaaatgagtaaaggagaagaacttttcactggag
GFP_rev aatagttgaactatttgtatagttcatccatgccatg

<u>*Can-elt-2* 3'UTR (251bp amplified from PS1010 genomic DNA)</u>

UTR_fwd atacaaatagttcaactatttttatcaataatttgctc
UTR_rev cgaggtcgacggtatcgatagaatgctcatcttcgcaaacttcgg

Development • Supplementary information

pGB598 Sequence:

aactagtgcattgtcagagagcccgtggttctgataccaatttttacagataaagttgttcccacacgtttttgagtttttttcgaaaaatatt
gattgacttacttactttgaactcaatttcaaactatctttcgaatattccaccttatttgtgagatgtctcttatttccctggaaaaatttgagtag
tctaggaaaacatctaaaattcagatttagaaattttctaaaagctttcaatttttcatttctattagattttttggggacagttagtttagtttttctt
atgaaaaacatcagaattttcagtttcctcattatccttctctctggccctgtaattctcggcattatacaaaactgtgtatttctggaacaaga
gaaaaacaattttgaattgtgtcccaaagttcaatcaataaaatgtatcgataagaagaggaaagaaaagtataagcggattacagct
aaaactataagcctacataataattcagaggtaggaaaaaacggaatttttagaatgagaaggtttttagctgactcgagacaatcttat
tttaaaaaattcttcaaaacatttcactacaataaacagtgaaatctggctttggtttaaacttcgaattattataggttttttatgaatacagc
tcaattcgataagaaatctgtgatttttgatgtttcaggtgtaggttgccaaataatgaataaggtaccagatttgaaacaatgatttaccaa
ttaaaaaccaagaaaagttaaagcttttagggtcaggagaacagtccaaaataattgttttctaacaaactaatcttctcaggccaccgt
taagttttgagcctaaaagatttttttttcgactttgttaaatttacccgacgaatatataaaaaaaatattcattatattcagactcactaatttc
atcagagttttccaaaattctgaaatctggatcagtttttagtgcctaaccaaaaaaaattggatattcatcaaatttcaccagtttcactaaat
cgatcttcgtgaacaacgtccgaactgtcagattgggataatcataaagctaaaaggaaccaaattaaaaactgattaagaaattcat
agaagataatgctttgaaatggaaagaaacaaccaaaaaaggttttagaattctaacagaagaaataaaaatatcagaccttcaact
cagtatttatattgaaaagataatgttatctttaattgatatattctattcatctgaaccatatcaagacaatcaattgtgtttttattgataaaaat
attgctttttttaaatttgaaaatgtttcgaatgtcccctataatttcagcattccccaattttgcttcccccacattgtagtataaagtcgatgaag
aaaaaagttgaccggcatcatataggcaactgataagaataccgtatactttcatgcacatcaactttttgtgtccacgacaatgcacatt
ggacctctgaatcatacaaaaaatgagaaataatttcgagaacaggatttcgaaaattagagaaaaatagaatgaaattatgatgtg
aaataatgttgcgaaaaaaactgattaaaaatatattgattacgttatcgaatacttggagtgatttatcaacttgtagagtccgcggtcct
gatataaaaaggtgtcagttcaacatatcgcctatcactggaacatataaactctaaaacttgataataagtattttcgtagttcattttcatg
tcataagaaattgaatcaagttttccagtgctatgcatgaatcgttaccagaatgaagaattttggttgaaaattttagtggagaatcatag
gttaggaaacatttgttcaacggtaactctttgttggcatactttgttgaaatctgaaaatttatgactggaaaactgaaggctaagctcca
aaaaacaatcttcattgataattttcatgtatttttcccaaaataacttttttttcagaaaattcataggagttgagagaatcagtttcaatcata
tcacaacttccataatatcgaaaatcaacttcgaattaaaattattttcctcaaagctctgggaaaatttcccaaacccttttttccagagcc
aatcaacaacgtttgccagctacagtaccaactcgcgactagaaaaggccatataaggagagggaaagagcactattattccctttttc
tcatttcttcattttccaatgtgtgcggggctcataatgactgataagataacgacagaatatttggcgttgggaagacagttgatggcttg
agcatattctttctttttttgtcggtatctctaacgaatatcttgaatttctctatttttttcataatctatttcgcaagttcttttttttcagaaaatggatc
caagatattcgatcgagagtccctggatcgatagtatgcgtccaccaacattgcaactcccaatccaacaaactcaaacacatactctt
atgcctgatgatgggcagagttcgatgagtgaactaactagaggtactagttttctgatgtttcatttttacaacattaatttattttcagttcga
actgatcctttgatggataggcaaagtgttatcggctcatttgatttacaatgtaagttttcgactaatagaaaaataatgatactgatcttca
tttttccagcaactttcgcaactggaacaaatcaagattatttgtataacacttcttcaatttttaacacatattcaactcaaccgcctccatca
caatatcatactttatcatcatcgtaagttggccgggaaattttgaattcttgaatgtttctgatttcacaataagaaaatattttcaagacatc
aaactccaatttttttaacagattttacgataattcggcatttataccacaacaacctttcgcgccagcaaatccagaatgtgtcaaatgcg
caaatccagtcatttccggaagacaagttgatggtggatatatgtgtgatacttgtagtaattctaatgtatatgatttgtcacgaattcaaa
caaactattcgatgccactttctcttgttcaaccaattgaacctgctccaattgaacaaattccacctccagcaccagcaccatcaaaac
caccaaaatcatcaagtaacaaaaaagctggatcagctggaagcacaaatcgccgacaaggattggtttgttcaaattgtaatggta
caaatactacactttggagaagaaatgctgaaggtgaaccagtttgcaagtgagttattcaaattttaaattggaaatatcagaaatgtg
aaagcttattttcagcgcatgtggtctgtatttcaaattgcatagtataaatcggccagtgtcaatgaaaaaagagggacaacttcaaac
aagaaaaaggtatttatttttcaaatgaaaacattgaacaacttgaaaaattatagaaaaatgaagaatagtgaatcatcaatgactcc
gacacgaccaaaagagcgtaaatatgaaaaacgaacaaaaacagaaacaactgaatatcaagcaaacgcatttgctttgaatgct
catcaacaaagccaaaatccttatagtaattcacatttcgcatatccagtgaacccattgcattatacaggtgagatcatttcgcaagtttt
atttcaaaatttttaaaatatatacgtttccagaaccatcgttagcttcattttcatatccacaatattcatcagaaatgaaaccaactattca
aatgatcaatcaagatgaagaagttaaagcagctgcgagagatctggaagaggaacaaatgagtaaaggagaagaacttttcact

Development • Supplementary information

ggagttgtcccaattcttgttgaattagatggtgatgttaatgggcacaaattttctgtcagtggagagggtgaaggtgatgcaacatacg
gaaaacttacccttaaatttatttgcactactggaaaactacctgttccatgggtaagtttaaacatatatatactaactaaccctgattattt
aaattttcagccaacacttgtcactactttctgttatggtgttcaatgcttctcgagatacccagatcatatgaaacggcatgactttttcaag
agtgccatgcccgaaggttatgtacaggaaagaactatatttttcaaagatgacgggaactacaagacacgtaagtttaaacagttcg
gtactaactaaccatacatatttaaattttcaggtgctgaagtcaagtttgaaggtgatacccttgttaatagaatcgagttaaaaggtattg
attttaaagaagatggaaacattcttggacacaaattggaatacaactataactcacacaatgtatacatcatggcagacaaacaaaa
gaatggaatcaaagttgtaagtttaaacatgattttactaactaactaatctgatttaaattttcagaacttcaaaattagacacaacattga
agatggaagcgttcaactagcagaccattatcaacaaaatactccaattggcgatggccctgtccttttaccagacaaccattacctgtc
cacacaatctgccctttcgaaagatcccaacgaaaagagagaccacatggtccttcttgagtttgtaacagctgctgggattacacatg
gcatggatgaactatacaaatagttcaactatttttatcaataatttgctctgatttacggcgtatttttaccttttttcatccatctcaaatagg at
atgtaaagattttgatacatatttatttttttgtcatatatactcaataaatttatatttcatattgttagtttaatgctttcagaaaaaaacacatca
ttcttgtgaaatataaattacagtagcatttccgaagtttgcgaagatgagcattctatcgataccgtcgacctcgagggggggcccggt
acccagcttttgttccctttagtgagggttaatttcgagcttggcgtaatcatggtcatagctgtttcctgtgtgaaattgttatccgctcacaatt
ccacacaacatacgagccggaagcataaagtgtaaagcctggggtgcctaatgagtgagctaactcacattaattgcgttgcgctca
ctgcccgctttccagtcgggaaacctgtcgtgccagctgcattaatgaatcggccaacgcgcggggagaggcggtttgcgtattgggc
gctcttccgcttcctcgctcactgactcgctgcgctcggtcgttcggctgcggcgagcggtatcagctcactcaaaggcggtaatacggtt
atccacagaatcaggggataacgcaggaaagaacatgtgagcaaaaggccagcaaaaggccaggaaccgtaaaaaggccgc
gttgctggcgtttttccataggctccgcccccctgacgagcatcacaaaaatcgacgctcaagtcagaggtggcgaaacccgacagg
actataaagataccaggcgtttccccctggaagctccctcgtgcgctctcctgttccgaccctgccgcttaccggatacctgtccgcctttc
tcccttcgggaagcgtggcgctttctcatagctcacgctgtaggtatctcagttcggtgtaggtcgttcgctccaagctgggctgtgtgcac
gaaccccccgttcagcccgaccgctgcgccttatccggtaactatcgtcttgagtccaacccggtaagacacgacttatcgccactgg
cagcagccactggtaacaggattagcagagcgaggtatgtaggcggtgctacagagttcttgaagtggtggcctaactacggctaca
ctagaaggacagtatttggtatctgcgctctgctgaagccagttaccttcggaaaaagagttggtagctcttgatccggcaaacaaacc
accgctggtagcggtggtttttttgtttgcaagcagcagattacgcgcagaaaaaaaggatctcaagaagatcctttgatcttttctacgg
ggtctgacgctcagtggaacgaaaactcacgttaagggattttggtcatgagattatcaaaaaggatcttcacctagatccttttaaatta
aaaatgaagttttaaatcaatctaaagtatatatgagtaaacttggtctgacagttaccaatgcttaatcagtgaggcacctatctcagcg
atctgtctatttcgttcatccatagttgcctgactccccgtcgtgtagataactacgatacgggagggcttaccatctggccccagtgctgc
aatgataccgcgagacccacgctcaccggctccagatttatcagcaataaaccagccagccggaagggccgagcgcagaagtgg
tcctgcaactttatccgcctccatccagtctattaattgttgccgggaagctagagtaagtagttcgccagttaatagtttgcgcaacgttgtt
gccattgctacaggcatcgtggtgtcacgctcgtcgtttggtatggcttcattcagctccggttcccaacgatcaaggcgagttacatgatc
ccccatgttgtgcaaaaaagcggttagctccttcggtcctccgatcgttgtcagaagtaagttggccgcagtgttatcactcatggttatgg
cagcactgcataattctcttactgtcatgccatccgtaagatgcttttctgtgactggtgagtactcaaccaagtcattctgagaatagtgtat
gcggcgaccgagttgctcttgcccggcgtcaatacgggataataccgcgccacatagcagaactttaaaagtgctcatcattggaaa
acgttcttcggggcgaaaactctcaaggatcttaccgctgttgagatccagttcgatgtaacccactcgtgcacccaactgatcttcagc
atcttttactttcaccagcgtttctgggtgagcaaaaacaggaaggcaaaatgccgcaaaaaagggaataagggcgacacggaaat
gttgaatactcatactcttcctttttcaatattattgaagcatttatcagggttattgtctcatgagcggatacatatttgaatgtatttagaaaaa
taaacaaataggggttccgcgcacatttccccgaaaagtgccacctgacgcgccctgtagcggcgcattaagcgcggcgggtgtggt
ggttacgcgcagcgtgaccgctacacttgccagcgccctagcgcccgctcctttcgctttcttcccttcctttctcgccacgttcgccggctt
tccccgtcaagctctaaatcgggggctccctttagggttccgatttagtgctttacggcacctcgaccccaaaaaacttgattagggtgat
ggttcacgtagtgggccatcgccctgatagacggtttttcgccctttgacgttggagtccacgttctttaatagtggactcttgttccaaactg
gaacaacactcaaccctatctcggtctattcttttgatttataagggattttgccgatttcggcctattggttaaaaaatgagctgatttaaca
aaaatttaacgcgaattttaacaaaatattaacgcttacaatttccattcgccattcaggctgcgcaactgttgggaagggcgatcggtg
cgggcctcttcgctattacgccagctggcgaaagggggatgtgctgcaaggcgattaagttgggtaacgccagggttttcccagtcac
gacgttgtaaaacgacggccagtgaattgtaatacgactcactatagggcgaattggagcttcatttattatatgtcattacggaaaacc
gaaaatcggtcgataaagaagaaagaaacagcatatatgaataaagaaagaaaacgacgcgacgtgaagcattatttgttttctgtc
tgcctgtctttttcgaaaggcaaaatatcaaaatctcgtcatatcctgcctaacttgacagactgtgcgaaaaatttgtgtgcgagaaaga
aacaaagcaacgacaatttagcggttggcaatacatacagatcgtgtgccaaaacgctttttttccggtttttgctttttgtgttttggcacaa

Development • Supplementary information

atcatttttatcacaaatcaaacaatctagatcacctcgtttttcttcaaaaaacttcaaaatatcatttctcctactctttttataaaaaatctc
atattcgaaaacaggaagggttgacaataaatgtgagatttttcgtctcttcggttctttggaaattgaaattttttgaacagacgaggtttga
atgaaaaaaatgaaaaaaagaaaaaaaactcaaattatatggaatgagggctgaaattattttgtgctctttttccgtttaatgaaaaa
atggagcaaattgaaactgaagaattgcaaattgggcaaatcatctgaaaaaatgtgggaaacaattaatttgtctagtttttctaacac
tcaattgaacgtttttgtgttttgatgttgtgacattgaaaatggaaggtttttcccaatttttcattggaaatgaatgacttctaattaaaaaga
gaaacaaaattggggtttttttcggaattcgttattttgagggaatacgtttgtgtgtgaaattcattgaaggaaaatcagtttttgaaatccc
tggaacgaaacattgaaaaaaaatgacgaacgtcctgaagatttcaaattttacaaaaaaataagaaatcgtcctggtaagttaaaa
atttttcctaagattattttgttgaaatttgaatcggacccttgaaatcaggcaaggcccaacttcaaaatataaactcttaaaaacgcggtt
ggtttttcggcatttttctatctcttgccgactcttcgagatcaatttaataactctcaaatagttttattttctgtatcgatttttccaggttttccgac
ttctcaaaagtttgccagaacattgtcttattcagactattcaaaagttcacaaaaacaccgcgtgatattttctcaaattccaatctgtttttta
tattcttcacattatacgataccgtatttaatccaatattcccaaaaaaaattgtatttttgtactaataaaactacatttcccaactttattattcgt
tattcattcctaaaaataataatactaatattcataaacaaatatccacatgttcgagggatcgacatatattactaatccaaaaatcgtctt
aacatcgacgtcgtaaggatatgtaatctacctaaaatcgtgtcgacgtaaataagaagatttgtttggtagtccaagcaagccacacc
tcatcctgactatttattccatctcatctcatctcatttcattttattttggtttgatttgttttgtcaaacgaaaaatggttgagctcaagtctctcact
gctccaaaagttgcgattccaattgttggactcattgtcttgctcgttgtttcaacattggttgctattttctatataaggtatgaacaataggaa
aggaaattgtattaatctgaaattttcagaaacccgggtgtcactgactcaatctttggaacttccaccgaagaaccgactactcctgcgt
cattttcgatgcaatccgctgatggcgaaatggaacatctccaggtaataaattatatttcaaaatgtcatctgattttttcttctcacaatca
tttcagaactcatctccacttggtcaatccgctcatcaaacaccaacaacaactgcacatgatcgttagtttgaatttgcgagaacagaa
aaaaacatataaaaagcatgttgtgtttttgatcaaagtgaatgatgataaatctttcaaagtttcagatgttttatttttccagtagctacaa
ccacttcttcctctccaattagtacaacaacttcttctccagttactcaaccttctcaacaactttccacacctttacatccaattgaaccaac
acaacatttaaaataattatttatttcaataaagttatttttctgactcattcaaaaaaaatgcaaaaaacttccctccaaaccccttttcgttct
tttattttccaaacaaatataaacgatcgaatataatttgctaataaatgtagggcaggcttggcgtcatgatgttactgataaggctacag
tacttctatattgctggcttgtcaaatatgtattatacgtgaataaacagaataaattcattttcagactagaaaacaggaaaaatagaattt
ttgttgaaaataagataaatttttgaaacatttatcaaaaagttggttatgattatgttatcgtcagaataatgtgtataaatatatttat

pGB608 *Can-elt-2::ELT-2::GFP*- smaller promoter- (diagrammed in pGB598)

all primers used to generate pGB608 are the same as for pGB598 except the Forward promoter primer :

<u>*Can-elt-2* promoter (2990 bp amplified from PS1010 genomic DNA):</u>

608F, cggccgctctagaactagtggatcaaagtgaatgatgataaatctttcaaag
Promoter reverse, gacgcatactatcgatccagggactctcgatcg

pGB612 *Cel-end-3::END-3upstream::Can-ELT-3_DBD::CFP*

Development • Supplementary information

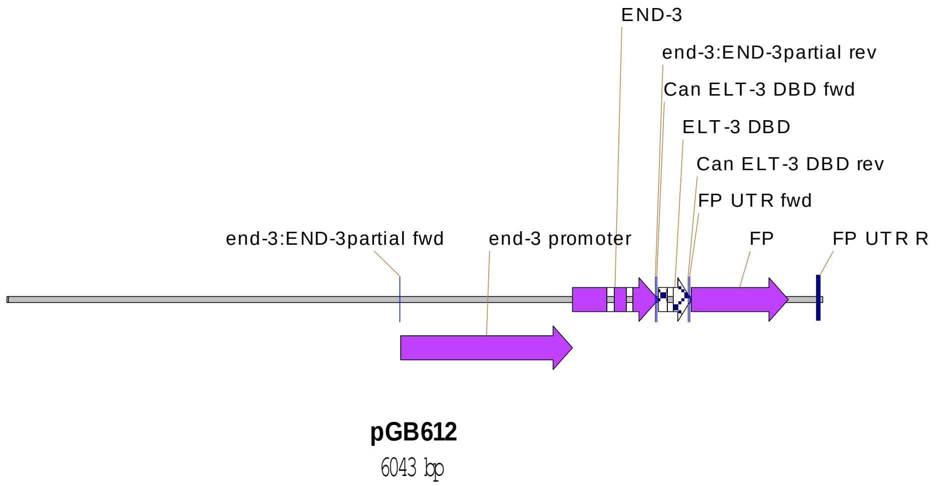

<u>*Cel-end-3* promoter and partial coding (1976bp from genomic DNA):</u>

end-3:END-3partial_fwd cggccgctctagaactagtgcatccaatttagtgtatatttatttcc
end-3:END-3partial_rev agtttgagcacgaagagttgatatgcattg

<u>Can-ELT-3 DBD (265 bp from PS1010)</u>

Can ELT-3 DBD_fwd caactcttcgtgctcaaactgcaagacac
Can ELT-3 DBD_rev ctttactcatgcatgtggcttgtcttctg

<u>CFP+UTR (988bp cassette)</u>

CFP was originally amplified from pEB1-SCFP3A and the *Cel-end-3* UTR was amplified from genomic DNA. The CFP and UTR were stitched together by PCR using the following primers:

CFP UTR_fwd agccacatgcatgagtaaaggagaagaac
CFP UTR_rev cgaggtcgacggtatcgataagctctttccactatagtc

pGB608 sequence

tatcgataccgtcgacctcgaggggggggcccggtacccagcttttgttccctttagtgagggttaatttcgagcttggcgtaatcatggtca
tagctgtttcctgtgtgaaattgttatccgctcacaattccacacaacatacgagccggaagcataaagtgtaaagcctggggtgcctaa
tgagtgagctaactcacattaattgcgttgcgctcactgcccgctttccagtcgggaaacctgtcgtgccagctgcattaatgaatcggcc
aacgcgcggggagaggcggtttgcgtattgggcgctcttccgcttcctcgctcactgactcgctgcgctcggtcgttcggctgcggcga
gcggtatcagctcactcaaaggcggtaatacggttatccacagaatcaggggataacgcaggaaagaacatgtgagcaaaaggc

Development • Supplementary information

cagcaaaaggccaggaaccgtaaaaaggccgcgttgctggcgtttttccataggctccgcccccctgacgagcatcacaaaaatcg
acgctcaagtcagaggtggcgaaacccgacaggactataaagataccaggcgtttccccctggaagctccctcgtgcgctctcctgtt
ccgaccctgccgcttaccggatacctgtccgcctttctcccttcgggaagcgtggcgctttctcatagctcacgctgtaggtatctcagttc
ggtgtaggtcgttcgctccaagctgggctgtgtgcacgaaccccccgttcagcccgaccgctgcgccttatccggtaactatcgtcttga
gtccaacccggtaagacacgacttatcgccactggcagcagccactggtaacaggattagcagagcgaggtatgtaggcggtgcta
cagagttcttgaagtggtggcctaactacggctacactagaaggacagtatttggtatctgcgctctgctgaagccagttaccttcggaa
aaagagttggtagctcttgatccggcaaacaaaccaccgctggtagcggtggtttttttgtttgcaagcagcagattacgcgcagaaaa
aaaggatctcaagaagatcctttgatcttttctacggggtctgacgctcagtggaacgaaaactcacgttaagggattttggtcatgagat
tatcaaaaaggatcttcacctagatccttttaaattaaaaatgaagttttaaatcaatctaaagtatatatgagtaaacttggtctgacagtt
accaatgcttaatcagtgaggcacctatctcagcgatctgtctatttcgttcatccatagttgcctgactccccgtcgtgtagataactacga
tacgggagggcttaccatctggccccagtgctgcaatgataccgcgagacccacgctcaccggctccagatttatcagcaataaacc
agccagccggaagggccgagcgcagaagtggtcctgcaactttatccgcctccatccagtctattaattgttgccgggaagctagagt
aagtagttcgccagttaatagtttgcgcaacgttgttgccattgctacaggcatcgtggtgtcacgctcgtcgtttggtatggcttcattcagc
tccggttcccaacgatcaaggcgagttacatgatcccccatgttgtgcaaaaaagcggttagctccttcggtcctccgatcgttgtcaga
agtaagttggccgcagtgttatcactcatggttatggcagcactgcataattctcttactgtcatgccatccgtaagatgcttttctgtgactg
gtgagtactcaaccaagtcattctgagaatagtgtatgcggcgaccgagttgctcttgcccggcgtcaatacgggataataccgcgcc
acatagcagaactttaaaagtgctcatcattggaaaacgttcttcggggcgaaaactctcaaggatcttaccgctgttgagatccagttc
gatgtaacccactcgtgcacccaactgatcttcagcatcttttactttcaccagcgtttctgggtgagcaaaaacaggaaggcaaaatg
ccgcaaaaaagggaataagggcgacacggaaatgttgaatactcatactcttcctttttcaatattattgaagcatttatcagggttattgt
ctcatgagcggatacatatttgaatgtatttagaaaaataaacaaataggggttccgcgcacatttccccgaaaagtgccacctgacgc
gccctgtagcggcgcattaagcgcggcgggtgtggtggttacgcgcagcgtgaccgctacacttgccagcgccctagcgcccgctcc
tttcgctttcttcccttcctttctcgccacgttcgccggctttccccgtcaagctctaaatcgggggctccctttagggttccgatttagtgcttta
cggcacctcgaccccaaaaaacttgattagggtgatggttcacgtagtgggccatcgccctgatagacggtttttcgccctttgacgttg
gagtccacgttctttaatagtggactcttgttccaaactggaacaacactcaaccctatctcggtctattcttttgatttataagggattttgcc
gatttcggcctattggttaaaaaatgagctgatttaacaaaaatttaacgcgaattttaacaaaatattaacgcttacaatttccattcgcca
ttcaggctgcgcaactgttgggaagggcgatcggtgcgggcctcttcgctattacgccagctggcgaaagggggatgtgctgcaagg
cgattaagttgggtaacgccagggttttcccagtcacgacgttgtaaaacgacggccagtgaattgtaatacgactcactatagggcg
aattggagctccaccgcggtggcggccgctctagaactagtgcatccaatttagtgtatatttatttcccttctcatattttctgtacaatttgc
atttctcttcaaccttgttctagttcatatccgtgtcaaaacttttattcatttcacttcgccaacttcagtcctactaaaatattgattctttgtaca
atgttgaatactttttctttttcttgatatatgaaattgttcacctgctttgaactgttgttaatgggtgttctggggttcacaaagtaaaataaga
gttttgcaaacttttgactgaaacgaaatttggacttttcaaacatttttacctaattttgagaaatgcatcttttggctcaaaggtgagactca
ctgtgtaggtctcgacgcgaacaaaaaatatttcgcgcaaaggcgtgtgttcctttaaatagtactgtcgccaattaatttttgcagtttcta
cagtatatcccacgtggtgtcagagtgtcccatatcggtttgatcttcgtagatctacgggaatagcggtaatttgacgcagatttctcaact
accaaaaatatttgttggttgagaattgaacagaaaaagaagaagaaaatacaagcatgtcgagctttacacaactttatctaataaat
attcaattaatttggatcagttttctctttcagcaattacaaaaaattttgttccgttaaaattgtgaatgattggatttggagagaatcgagttttt
ggaaaaataaaaaacaagagcaacgtgttatggtattattcatgtgatcttttaaaatgcaaacgtgtattttaatttaattgttcccctatca
gaacctctctaaatttcacgtgtttcaaattccctctttttctctctactctttcactctcgcacgtgaaaaacttctttccgaatgataatttgtgttt
aaaatggaaattttattgaagtaaaatttatgaaacaaatacttttagtttaaatccgagatgcacttctatttgagagagcccacggtaaa
ttaaaaaagaagtaaaagtataccaaacaagtatgttccaagtgttccgatggctccgacaaaagagtatacactgattgtataatcg
gttagatgaatagctgaaagtataccacgttcaaagcacaccccgccttgtgtctcttctagaaaaaggcggttgctgaaaagtatacg
gaacgctgaggcacgccctcttcttaccatgatttgctccaagataaatgaataggcattctcattcaaagtataaacatgtactcgaact
cattctcttcatcatcttcatcttccaactcaccaatgtcttttgattttggattccctcagtttcccgaacaagtccaattcaatgaagaaggct
acgggacaccatctccagatgtcctccagaatatgaattatcatcattatccggcacaagatatgacgacaaattcatacaatggagg
ctatgacaattcaatgcaacagaattttatgcaaccagataatggtgcaggatactacagtgagttttaaaaaactattttattttacttttat
atttaaaattaatgtttcagatgaaaactatcaacagatgcctgattttcaatttccagttcaaaattttgattttacaaatcaatttgaattcac
gacgcctattaatggtataattaactatgaaacttacagcctaatcttaaatttattttagacctccagcaatctcaaacttccatcaaccac
accaatcccgacaacaatgaaaattccatgccagagataccaattgacgggggcttcaactttttttccagctcaagaagttcaagaat

Development • Supplementary information

ggaagccagctagaaaagcttcgaaaaataaaatcaagaagatttccacaatgcatatcaactcttcgtgctcaaactgcaagacac
gtgaaactacactttggcgacgaaatggtgaaggaggtgttgagtgcaagtaagttgttctaactcgaaaaaaaaaattaaccaaattt
tttgcagcgcttgcaatttgtatttccgcaaaaacaatcgaaaaaggccgttgtcattgagaaaagatggaataatgaaacgaaatcgt
cgtccaagaaacgaatcaccaaatgggatcagaagacaagccacatgcatgagtaaaggagaagaacttttcactggagttgtcc
caattcttgttgaattagatggtgatgttaatgggcacaaattttctgtcagtggagagggtgaaggtgatgcaacatacggaaaacttac
ccttaaatttatttgcactactggaaaactacctgttccatggccaacacttgtcactactctcacttggggtgttcaatgctttgcaagatac
ccagatcatatgaaacagcatgactttttcaagagtgccatgcccgaaggttatgtacaggaaagaactatatttttcaaagatgacgg
gaactacaagacacgtgctgaagtcaagtttgaaggtgatacccttgttaatagaatcgagttaaaaggtattgattttaaagaagatgg
aaacattcttggacacaaattggaatacaactacatctcagacaatgtatacatcacggcagacaaacaaaagaatggaatcaaag
ctaacttcaaaattagacacaacattgaagatggaggcgttcaactagcagaccattatcaacaaaatactccaattggcgatggccc
tgtccttttaccagacaaccattacctgtccacacaatctaagctttcgaaagatcccaacgaaaagagagaccacatggtccttcttga
gtttgtaacagctgctgggattacacatggcatggatgaactatacaaataacaactgatcaactctgtgcaataagcattattatttttcta
atcaaacatgacctttcggttataatttaactatttgttttatttcccctctgctggtttccataataaataaataaaaaaacttcatgctcttgat
actacagttttccagatcaattgtgaatttcgaacagcttgttcagatttgaaatgtagtgttgcaagaattgatttcctgtgtggagtgtgaa
caataagcatgactatagtggaaagagct

pGB618: *Cel-end-3::Can-ELT-3B::CFP*

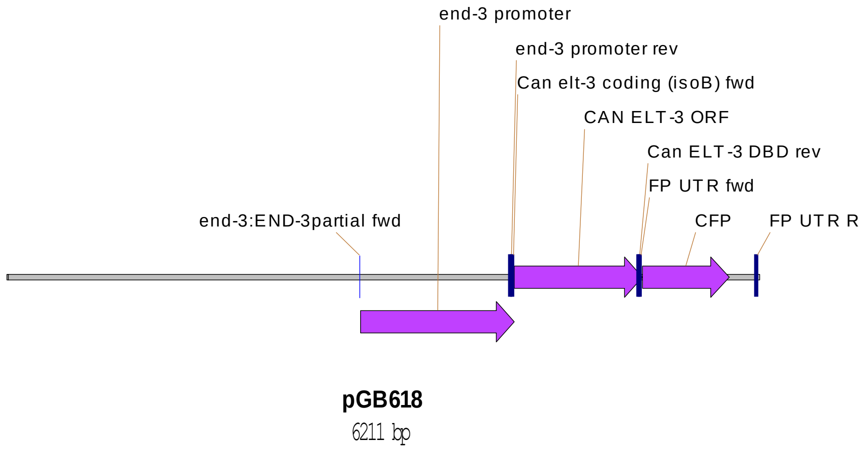

<u>*Cel-elt-3* promoter (1290 bp amplified from N2 genomic DNA)</u>

end-3:END-3partial_fwd cggccgctctagaactagtgcatccaatttagtgtatatttatttcc
end-3 promoter_rev gagtattcatgtttatactttgaatgagaatgc

Development • Supplementary information

<u>*Can-elt-3* coding (1076 bp amplified from IDT synthesized minigene)</u>

Can elt-3 coding (isoB)_fwd aagtataaacatgaatactcaaagtcctctcg
Can ELT-3 DBD_rev ctttactcatgcatgtggcttgtcttctg

<u>CFP UTR (988bp cassette –as in pGB612 (see above)</u>

pGB618 sequence

tatcgataccgtcgacctcgagggggggcccggtacccagcttttgttccctttagtgagggttaatttcgagcttggcgtaatcatggtca
tagctgtttcctgtgtgaaattgttatccgctcacaattccacacaacatacgagccggaagcataaagtgtaaagcctggggtgcctaa
tgagtgagctaactcacattaattgcgttgcgctcactgcccgctttccagtcgggaaacctgtcgtgccagctgcattaatgaatcggcc
aacgcgcggggagaggcggtttgcgtattgggcgctcttccgcttcctcgctcactgactcgctgcgctcggtcgttcggctgcggcga
gcggtatcagctcactcaaaggcggtaatacggttatccacagaatcaggggataacgcaggaaagaacatgtgagcaaaaggc
cagcaaaaggccaggaaccgtaaaaaggccgcgttgctggcgtttttccataggctccgcccccctgacgagcatcacaaaaatcg
acgctcaagtcagaggtggcgaaacccgacaggactataaagataccaggcgtttccccctggaagctccctcgtgcgctctcctgtt
ccgaccctgccgcttaccggatacctgtccgcctttctcccttcgggaagcgtggcgctttctcatagctcacgctgtaggtatctcagttc
ggtgtaggtcgttcgctccaagctgggctgtgtgcacgaaccccccgttcagcccgaccgctgcgccttatccggtaactatcgtcttga
gtccaacccggtaagacacgacttatcgccactggcagcagccactggtaacaggattagcagagcgaggtatgtaggcggtgcta
cagagttcttgaagtggtggcctaactacggctacactagaaggacagtatttggtatctgcgctctgctgaagccagttaccttcggaa
aaagagttggtagctcttgatccggcaaacaaaccaccgctggtagcggtggtttttttgtttgcaagcagcagattacgcgcagaaaa
aaaggatctcaagaagatcctttgatcttttctacggggtctgacgctcagtggaacgaaaactcacgttaagggattttggtcatgagat
tatcaaaaaggatcttcacctagatccttttaaattaaaaatgaagttttaaatcaatctaaagtatatatgagtaaacttggtctgacagtt
accaatgcttaatcagtgaggcacctatctcagcgatctgtctatttcgttcatccatagttgcctgactccccgtcgtgtagataactacga
tacgggagggcttaccatctggccccagtgctgcaatgataccgcgagacccacgctcaccggctccagatttatcagcaataaacc
agccagccggaagggccgagcgcagaagtggtcctgcaactttatccgcctccatccagtctattaattgttgccgggaagctagagt
aagtagttcgccagttaatagtttgcgcaacgttgttgccattgctacaggcatcgtggtgtcacgctcgtcgtttggtatggcttcattcagc
tccggttcccaacgatcaaggcgagttacatgatcccccatgttgtgcaaaaaagcggttagctccttcggtcctccgatcgttgtcaga
agtaagttggccgcagtgttatcactcatggttatggcagcactgcataattctcttactgtcatgccatccgtaagatgcttttctgtgactg
gtgagtactcaaccaagtcattctgagaatagtgtatgcggcgaccgagttgctcttgcccggcgtcaatacgggataataccgcgcc
acatagcagaactttaaaagtgctcatcattggaaaacgttcttcggggcgaaaactctcaaggatcttaccgctgttgagatccagttc
gatgtaacccactcgtgcacccaactgatcttcagcatcttttactttcaccagcgtttctgggtgagcaaaaacaggaaggcaaaatg
ccgcaaaaaagggaataagggcgacacggaaatgttgaatactcatactcttcctttttcaatattattgaagcatttatcagggttattgt
ctcatgagcggatacatatttgaatgtatttagaaaaataaacaaataggggttccgcgcacatttccccgaaaagtgccacctgacgc
gccctgtagcggcgcattaagcgcggcgggtgtggtggttacgcgcagcgtgaccgctacacttgccagcgccctagcgcccgctcc
tttcgctttcttcccttcctttctcgccacgttcgccggctttccccgtcaagctctaaatcgggggctccctttagggttccgatttagtgcttta
cggcacctcgaccccaaaaaacttgattagggtgatggttcacgtagtgggccatcgccctgatagacggtttttcgccctttgacgttg
gagtccacgttctttaatagtggactcttgttccaaactggaacaacactcaaccctatctcggtctattcttttgatttataagggattttgcc
gatttcggcctattggttaaaaaatgagctgatttaacaaaaatttaacgcgaattttaacaaaatattaacgcttacaatttccattcgcca
ttcaggctgcgcaactgttgggaagggcgatcggtgcgggcctcttcgctattacgccagctggcgaaagggggatgtgctgcaagg
cgattaagttgggtaacgccagggttttcccagtcacgacgttgtaaaacgacggccagtgaattgtaatacgactcactatagggcg
aattggagctccaccgcggtggcggccgctctagaactagtgcatccaatttagtgtatatttatttcccttctcatattttctgtacaatttgc
atttctcttcaaccttgttctagttcatatccgtgtcaaaacttttattcatttcacttcgccaacttcagtcctactaaaatattgattctttgtaca

Development • Supplementary information

atgttgaatactttttctttttcttgatatatgaaattgttcacctgctttgaactgttgttaatgggtgttctggggttcacaaagtaaaataaga
gttttgcaaacttttgactgaaacgaaatttggacttttcaaacatttttacctaattttgagaaatgcatcttttggctcaaaggtgagactca
ctgtgtaggtctcgacgcgaacaaaaaatatttcgcgcaaaggcgtgtgttcctttaaatagtactgtcgccaattaatttttgcagtttcta
cagtatatcccacgtggtgtcagagtgtcccatatcggtttgatcttcgtagatctacgggaatagcggtaatttgacgcagatttctcaact
accaaaaatatttgttggttgagaattgaacagaaaaagaagaagaaaatacaagcatgtcgagctttacacaactttatctaataaat
attcaattaatttggatcagttttctctttcagcaattacaaaaaattttgttccgttaaaattgtgaatgattggatttggagagaatcgagttttt
ggaaaaataaaaaacaagagcaacgtgttatggtattattcatgtgatcttttaaaatgcaaacgtgtattttaatttaattgttcccctatca
gaacctctctaaatttcacgtgtttcaaattccctctttttctctctactctttcactctcgcacgtgaaaaacttctttccgaatgataatttgtgttt
aaaatggaaattttattgaagtaaaatttatgaaacaaatacttttagtttaaatccgagatgcacttctatttgagagagcccacggtaaa
ttaaaaaagaagtaaaagtataccaaacaagtatgttccaagtgttccgatggctccgacaaaagagtatacactgattgtataatcg
gttagatgaatagctgaaagtataccacgttcaaagcacaccccgccttgtgtctcttctagaaaaaggcggttgctgaaaagtatacg
gaacgctgaggcacgccctcttcttaccatgatttgctccaagataaatgaataggcattctcattcaaagtataaacatgaatactcaa
agtcctctcgggattcaaacctactacccaaactcaccgcattcaagcactgatacatcatcacgtggtaagttactcatgatgaataca
ccaattccaagtgatacatattcgccagcaaattatcatcatcttcatcatcaacaaaatcagaatcacaatcaaagtcagaatcataat
caagtgcaaaattatcaagatcatcaatttgagcaacagtatcaagaatatcatcaccaccaagaggcgcagccacaccatcaact
accaccacaattcgagccaacaaatagttcaaattcacgagcttattattgtccatatccatcaacaacggagacaatacaagctccg
gttactgtcgcctctgctgctccccttccagctatcgctcatttaatgaaggaagttcagatcaaagaggagtttaacgaatattatgctcc
caatccatcaatgaacgactatcgcgttgaaaagattgccaatccgttggctgacccatatattcaaattgagcaaccaatttataccga
ttttgcaaatgctccgatgctcaatcaacaagatatgctaaacccgatgaatttctccaccaactacatgggagccacacaattctccca
ccaacagactgtgcaactcaatttgctagatctcagcggcttctccacattcaactcgtgtgatccagctctaccattaatgcaccagtca
ccttctcaacaaaattatgtgatgccacattcaacttttacaccacctccacaagatccattggtcgctgaacaaaaaccacttgtcaaa
aagcgaatggctgccgtgcaatgtcatcaaaattcaatttgctcaaactgcaagacacgtgaaactacactttggcgacgaaatggtg
aaggaggtgttgagtgcaacgcttgcaatttgtatttccgcaaaaacaatcgaaaaaggccgttgtcattgagaaaagatggaataat
gaaacgaaatcgtcgtccaagaaacgaatcaccaaatgggatcagaagacaagccacatgcatgagtaaaggagaagaactttt
cactggagttgtcccaattcttgttgaattagatggtgatgttaatgggcacaaattttctgtcagtggagagggtgaaggtgatgcaacat
acggaaaacttacccttaaatttatttgcactactggaaaactacctgttccatggccaacacttgtcactactctcacttggggtgttcaat
gctttgcaagatacccagatcatatgaaacagcatgactttttcaagagtgccatgcccgaaggttatgtacaggaaagaactatattttt
caaagatgacgggaactacaagacacgtgctgaagtcaagtttgaaggtgatacccttgttaatagaatcgagttaaaaggtattgatt
ttaaagaagatggaaacattcttggacacaaattggaatacaactacatctcagacaatgtatacatcacggcagacaaacaaaag
aatggaatcaaagctaacttcaaaattagacacaacattgaagatggaggcgttcaactagcagaccattatcaacaaaatactcca
attggcgatggccctgtccttttaccagacaaccattacctgtccacacaatctaagctttcgaaagatcccaacgaaaagagagacc
acatggtccttcttgagtttgtaacagctgctgggattacacatggcatggatgaactatacaaataatcaactctgtgcaataagcattat
tatttttctaatcaaacatgacctttcggttataatttaactatttgttttatttcccctctgctggtttccataataaataaataaaaaaacttcatg
ctcttgatactacagttttccagatcaattgtgaatttcgaacagcttgttcagatttgaaatgtagtgttgcaagaattgatttcctgtgtgga
gtgtgaacaataagcatgactatagtggaaagagct

pGB619 (*hsp16.41promoter::ELT-3B::CFP::end-3_3'UTR*)

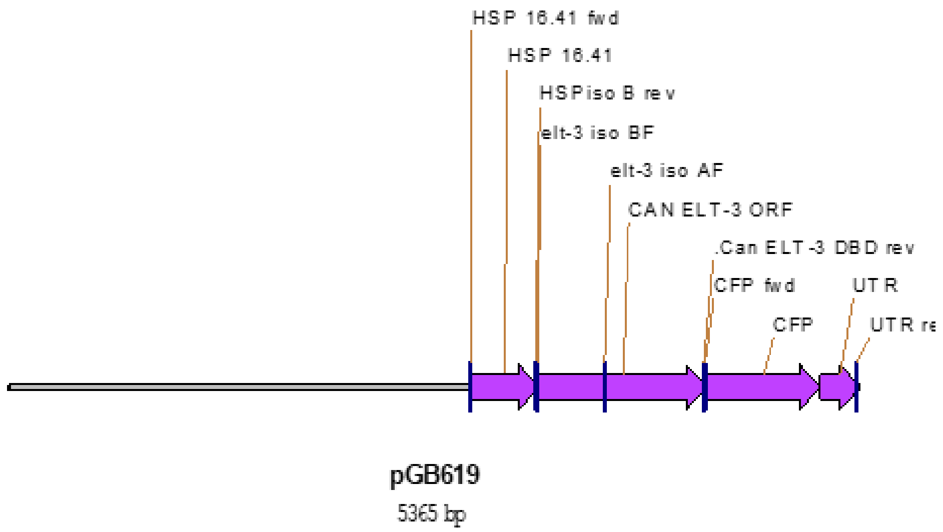

Heat-shock promoter (444bp amplified from pPD49.83)

HSP 16.41_fwd cggccgctctagaactagtggatcaccaaaaacggaac
HSPiso B rev gagtattcatggatcccgatgaggattttc

Can-elt-3B coding (1076 bp amplified from IDT synthesized minigene)

elt-3 iso BF, atcgggatccatgaatactcaaagtcctctcgggattcaa
Can ELT-3 DBD_rev ctttactcatgcatgtggcttgtcttctg

CFP UTR (988bp cassette –as in pGB612 (see above)

pGB619 sequence

tatcgataccgtcgacctcgagggggggcccggtacccagcttttgttccctttagtgagggttaatttcgagcttggcgtaatcatggtca
tagctgtttcctgtgtgaaattgttatccgctcacaattccacacaacatacgagccggaagcataaagtgtaaagcctggggtgcctaa
tgagtgagctaactcacattaattgcgttgcgctcactgcccgctttccagtcgggaaacctgtcgtgccagctgcattaatgaatcggcc
aacgcgcggggagaggcggtttgcgtattgggcgctcttccgcttcctcgctcactgactcgctgcgctcggtcgttcggctgcggcga
gcggtatcagctcactcaaaggcggtaatacggttatccacagaatcaggggataacgcaggaaagaacatgtgagcaaaaggc
cagcaaaaggccaggaaccgtaaaaaggccgcgttgctggcgtttttccataggctccgcccccctgacgagcatcacaaaaatcg
acgctcaagtcagaggtggcgaaacccgacaggactataaagataccaggcgtttccccctggaagctccctcgtgcgctctcctgtt
ccgaccctgccgcttaccggatacctgtccgcctttctcccttcgggaagcgtggcgctttctcatagctcacgctgtaggtatctcagttc
ggtgtaggtcgttcgctccaagctgggctgtgtgcacgaaccccccgttcagcccgaccgctgcgccttatccggtaactatcgtcttga
gtccaacccggtaagacacgacttatcgccactggcagcagccactggtaacaggattagcagagcgaggtatgtaggcggtgcta

Development • Supplementary information

cagagttcttgaagtggtggcctaactacggctacactagaaggacagtatttggtatctgcgctctgctgaagccagttaccttcggaa
aaagagttggtagctcttgatccggcaaacaaaccaccgctggtagcggtggtttttttgtttgcaagcagcagattacgcgcagaaaa
aaaggatctcaagaagatcctttgatcttttctacggggtctgacgctcagtggaacgaaaactcacgttaagggattttggtcatgagat
tatcaaaaaggatcttcacctagatccttttaaattaaaaatgaagttttaaatcaatctaaagtatatatgagtaaacttggtctgacagtt
accaatgcttaatcagtgaggcacctatctcagcgatctgtctatttcgttcatccatagttgcctgactccccgtcgtgtagataactacga
tacgggagggcttaccatctggccccagtgctgcaatgataccgcgagacccacgctcaccggctccagatttatcagcaataaacc
agccagccggaagggccgagcgcagaagtggtcctgcaactttatccgcctccatccagtctattaattgttgccgggaagctagagt
aagtagttcgccagttaatagtttgcgcaacgttgttgccattgctacaggcatcgtggtgtcacgctcgtcgtttggtatggcttcattcagc
tccggttcccaacgatcaaggcgagttacatgatcccccatgttgtgcaaaaaagcggttagctccttcggtcctccgatcgttgtcaga
agtaagttggccgcagtgttatcactcatggttatggcagcactgcataattctcttactgtcatgccatccgtaagatgcttttctgtgactg
gtgagtactcaaccaagtcattctgagaatagtgtatgcggcgaccgagttgctcttgcccggcgtcaatacgggataataccgcgcc
acatagcagaactttaaaagtgctcatcattggaaaacgttcttcggggcgaaaactctcaaggatcttaccgctgttgagatccagttc
gatgtaacccactcgtgcacccaactgatcttcagcatcttttactttcaccagcgtttctgggtgagcaaaaacaggaaggcaaaatg
ccgcaaaaaagggaataagggcgacacggaaatgttgaatactcatactcttcctttttcaatattattgaagcatttatcagggttattgt
ctcatgagcggatacatatttgaatgtatttagaaaaataaacaaataggggttccgcgcacatttccccgaaaagtgccacctgacgc
gccctgtagcggcgcattaagcgcggcgggtgtggtggttacgcgcagcgtgaccgctacacttgccagcgccctagcgcccgctcc
tttcgctttcttcccttcctttctcgccacgttcgccggctttccccgtcaagctctaaatcgggggctccctttagggttccgatttagtgcttta
cggcacctcgaccccaaaaaacttgattagggtgatggttcacgtagtgggccatcgccctgatagacggtttttcgccctttgacgttg
gagtccacgttctttaatagtggactcttgttccaaactggaacaacactcaaccctatctcggtctattcttttgatttataagggattttgcc
gatttcggcctattggttaaaaaatgagctgatttaacaaaaatttaacgcgaattttaacaaaatattaacgcttacaatttccattcgcca
ttcaggctgcgcaactgttgggaagggcgatcggtgcgggcctcttcgctattacgccagctggcgaaagggggatgtgctgcaagg
cgattaagttgggtaacgccagggttttcccagtcacgacgttgtaaaacgacggccagtgaattgtaatacgactcactatagggcg
aattggagctccaccgcggtggcggccgctctagaactagtggatccaccaaaaacggaacgttgagctggacggaaatagtggta
aagtgacatgattatagtttgaagatttctaatttcacaattagagcaaatgttgttcggtatttattttcaacggtatttatactattttccacttt
ttctagaacattcgagctgcttgttgcaaaaggagggcgactcacattcggtacatggaaaagtagtgtacacaataaagagaccca
gatacattttccgtctgcgtctctttgcacccaccgggagtattttcaaacgaatgcatctaggaccttctagaacattctgtaaggctgca
gaatgcgggtatataaggaaagcgggctcagaggaagccaacacgctttgttctagtgcatctaaaaaacttcgaaaatcctcatcg
ggatccatgaatactcaaagtcctctcgggattcaaacctactacccaaactcaccgcattcaagcactgatacatcatcacgtggtaa
gttactcatgatgaatacaccaattccaagtgatacatattcgccagcaaattatcatcatcttcatcatcaacaaaatcagaatcacaat
caaagtcagaatcataatcaagtgcaaaattatcaagatcatcaatttgagcaacagtatcaagaatatcatcaccaccaagaggcg
cagccacaccatcaactaccaccacaattcgagccaacaaatagttcaaattcacgagcttattattgtccatatccatcaacaacgg
agacaatacaagctccggttactgtcgcctctgctgctccccttccagctatcgctcatttaatgaaggaagttcagatcaaagaggagt
ttaacgaatattatgctcccaatccatcaatgaacgactatcgcgttgaaaagattgccaatccgttggctgacccatatattcaaattga
gcaaccaatttataccgattttgcaaatgctccgatgctcaatcaacaagatatgctaaacccgatgaatttctccaccaactacatggg
agccacacaattctcccaccaacagactgtgcaactcaatttgctagatctcagcggcttctccacattcaactcgtgtgatccagctcta
ccattaatgcaccagtcaccttctcaacaaaattatgtgatgccacattcaacttttacaccacctccacaagatccattggtcgctgaac
aaaaaccacttgtcaaaaagcgaatggctgccgtgcaatgtcatcaaaattcaatttgctcaaactgcaagacacgtgaaactacact
ttggcgacgaaatggtgaaggaggtgttgagtgcaacgcttgcaatttgtatttccgcaaaaacaatcgaaaaaggccgttgtcattga
gaaaagatggaataatgaaacgaaatcgtcgtccaagaaacgaatcaccaaatgggatcagaagacaagccacatgcatgagta
aaggagaagaacttttcactggagttgtcccaattcttgttgaattagatggtgatgttaatgggcacaaattttctgtcagtggagagggt
gaaggtgatgcaacatacggaaaacttacccttaaatttatttgcactactggaaaactacctgttccatggccaacacttgtcactactc
tcacttggggtgttcaatgctttgcaagatacccagatcatatgaaacagcatgactttttcaagagtgccatgcccgaaggttatgtaca
ggaaagaactatatttttcaaagatgacgggaactacaagacacgtgctgaagtcaagtttgaaggtgatacccttgttaatagaatcg
agttaaaaggtattgattttaaagaagatggaaacattcttggacacaaattggaatacaactacatctcagacaatgtatacatcacg
gcagacaaacaaaagaatggaatcaaagctaacttcaaaattagacacaacattgaagatggaggcgttcaactagcagaccatt
atcaacaaaatactccaattggcgatggccctgtccttttaccagacaaccattacctgtccacacaatctaagctttcgaaagatccca
acgaaaagagagaccacatggtccttcttgagtttgtaacagctgctgggattacacatggcatggatgaactatacaaataatcaact

Development • Supplementary information

ctgtgcaataagcattattatttttctaatcaaacatgacctttcggttataatttaactatttgttttatttcccctctgctggtttccataataaata
aataaaaaaacttcatgctcttgatactacagttttccagatcaattgtgaatttcgaacagcttgttcagatttgaaatgtagtgttgcaag
aattgatttcctgtgtggagtgtgaacaataagcatgactatagtggaaagagct

pGB620 *hsp16-41promoter::ELT-3A::CFP::end-3_3'UTR* - see notation in pGB619

Heat-shock promoter (444bp amplified from pPD49.83)

HSP 16.41_fwd cggccgctctagaactagtggatcaccaaaaacggaac
HSP16.41 isoArev cttccttcatggatcccgatgaggattttc

Can elt-3 coding isoA (665bp amplified from IDT synthesized minigene (noted in pGB619 above)

elt-3 isoAF atcgggatccatgaaggaagttcagatcaaagaggag
Can ELT-3 DBD_rev ctttactcatgcatgtggcttgtcttctg

CFP UTR 988bp cassette –as in pGB612 (see above)

4) smiFISH PROBESETS:

Conjugated FLAP-X oligos (CACTGAGTCCAGCTCGAAACTTAGGAGG) that were 5′ and 3′ end-labelled with Quasar 570 or CalFluor 610 were synthesized by Biosearch Technologies. FLAP-X oligos that were 5′ and 3′ end-labelled with Cy5 or Cy3 were synthesized by IDT.

smiFISH antisense probesets (with complementary FLAP-X sequence added to 5' end of oligo (CAPS)):

Can-skn-1: 19 probes

CCTCCTAAGTTTCGAGCTGGACTCAGTGttcctttcgacaaatcttcata
CCTCCTAAGTTTCGAGCTGGACTCAGTGcaaatcttcatcgcttggaatt
CCTCCTAAGTTTCGAGCTGGACTCAGTGttcttcttttgaaacatcttcc
CCTCCTAAGTTTCGAGCTGGACTCAGTGtgtctgcacaatattttgttga
CCTCCTAAGTTTCGAGCTGGACTCAGTGtcgctcaatgaaacattttgca
CCTCCTAAGTTTCGAGCTGGACTCAGTGatgctcatttcggttaaattgt
CCTCCTAAGTTTCGAGCTGGACTCAGTGattgaaaaacgtctcagcttgc
CCTCCTAAGTTTCGAGCTGGACTCAGTGttgttgcattgtattcgaatca
CCTCCTAAGTTTCGAGCTGGACTCAGTGgtgtcgttgatcattggaatat
CCTCCTAAGTTTCGAGCTGGACTCAGTGttggtggaattctcgaatatct

Development • Supplementary information

CCTCCTAAGTTTCGAGCTGGACTCAGTGcaagttgatcatgattagctgg
CCTCCTAAGTTTCGAGCTGGACTCAGTGgtctcatcgaatgatatggatc
CCTCCTAAGTTTCGAGCTGGACTCAGTGtcgatgacgtatcaacatcaga
CCTCCTAAGTTTCGAGCTGGACTCAGTGctctctattatatcttggtgat
CCTCCTAAGTTTCGAGCTGGACTCAGTGatagaatctgctagctggaatt
CCTCCTAAGTTTCGAGCTGGACTCAGTGagtcaatggaatgacacgtgag
CCTCCTAAGTTTCGAGCTGGACTCAGTGttgttcatcttttgattgtctt
CCTCCTAAGTTTCGAGCTGGACTCAGTGctcatttcgcttatttgatgag
CCTCCTAAGTTTCGAGCTGGACTCAGTGatgtgtcgattttgagtacttg

Cmo-elt-3.2: 24 probes

CCTCCTAAGTTTCGAGCTGGACTCAGTGaaaagcggcgggaactcgaa
CCTCCTAAGTTTCGAGCTGGACTCAGTGcgtgtcgcatcactgttagc
CCTCCTAAGTTTCGAGCTGGACTCAGTGttgtccaagtcctcgaactc
CCTCCTAAGTTTCGAGCTGGACTCAGTGtcgtcgttcggccgaaagta
CCTCCTAAGTTTCGAGCTGGACTCAGTGctgaatctccggcaacggag
CCTCCTAAGTTTCGAGCTGGACTCAGTGcgtcgatgggcgtcaagaag
CCTCCTAAGTTTCGAGCTGGACTCAGTGaaagtcttcgtaggccggtg
CCTCCTAAGTTTCGAGCTGGACTCAGTGcatacgcgacgtgcttgtgg
CCTCCTAAGTTTCGAGCTGGACTCAGTGgctatcgaaatgtagtggcc
CCTCCTAAGTTTCGAGCTGGACTCAGTGatctgcacgacttggctgta
CCTCCTAAGTTTCGAGCTGGACTCAGTGcgtcgcatatccatcgtagg
CCTCCTAAGTTTCGAGCTGGACTCAGTGaatagacgtcgccctcgaac
CCTCCTAAGTTTCGAGCTGGACTCAGTGctcttcttctcctcgacgag
CCTCCTAAGTTTCGAGCTGGACTCAGTGacttggagtccttgtgacac
CCTCCTAAGTTTCGAGCTGGACTCAGTGtctcgcgggttttgcaattg
CCTCCTAAGTTTCGAGCTGGACTCAGTGaagcattgcactcgatctcg
CCTCCTAAGTTTCGAGCTGGACTCAGTGtcttccggaagtacaggttg
CCTCCTAAGTTTCGAGCTGGACTCAGTGtcagcggacgtgcaaagttg
CCTCCTAAGTTTCGAGCTGGACTCAGTGtcttgacgatgtggttcttg
CCTCCTAAGTTTCGAGCTGGACTCAGTGtcgtagtagacgtagcgctc
CCTCCTAAGTTTCGAGCTGGACTCAGTGaacacacgttcttggcgttg
CCTCCTAAGTTTCGAGCTGGACTCAGTG tcaggtgtgccccaagagct
CCTCCTAAGTTTCGAGCTGGACTCAGTG gtggcgtactgctgctcgcc
CCTCCTAAGTTTCGAGCTGGACTCAGTGtgcttcatcatccgatgcat

Can-elt-3 (probeset1, detects both isoforms): 24 probes

CCTCCTAAGTTTCGAGCTGGACTCAGTGggattgggagctgtaaataac
CCTCCTAAGTTTCGAGCTGGACTCAGTGaacgcgatagtcgttcattga
CCTCCTAAGTTTCGAGCTGGACTCAGTGccaacggattggcaatctttt
CCTCCTAAGTTTCGAGCTGGACTCAGTGtcggtataaattggttgctca
CCTCCTAAGTTTCGAGCTGGACTCAGTGgattgagcatcggagcatttg
CCTCCTAAGTTTCGAGCTGGACTCAGTGcatcgggtttagcatatcttg
CCTCCTAAGTTTCGAGCTGGACTCAGTGccatgtagttggtggagaaat
CCTCCTAAGTTTCGAGCTGGACTCAGTGtgttggtgggagaattgtgtg

Development • Supplementary information

CCTCCTAAGTTTCGAGCTGGACTCAGTGtctagcaaattgagttgcaca
CCTCCTAAGTTTCGAGCTGGACTCAGTGacgagttgaatgtggagaagc
CCTCCTAAGTTTCGAGCTGGACTCAGTGattaatggtagagctggatca
CCTCCTAAGTTTCGAGCTGGACTCAGTGttgttgagaaggtgactggtg
CCTCCTAAGTTTCGAGCTGGACTCAGTGgttgaatgtggcatcacataa
CCTCCTAAGTTTCGAGCTGGACTCAGTGatcttgtggaggtggtgtaaa
CCTCCTAAGTTTCGAGCTGGACTCAGTGgtttttgttcagcgaccaatg
CCTCCTAAGTTTCGAGCTGGACTCAGTGagccattcgctttttgacaag
CCTCCTAAGTTTCGAGCTGGACTCAGTGaattttgatgacattgcacgg
CCTCCTAAGTTTCGAGCTGGACTCAGTGtgtcttgcagtttgagcaaat
CCTCCTAAGTTTCGAGCTGGACTCAGTGcatttcgtcgccaaagtgtag
CCTCCTAAGTTTCGAGCTGGACTCAGTGtacaaattgcaagcgttgcac
CCTCCTAAGTTTCGAGCTGGACTCAGTGtttttcgattgtttttgcgga
CCTCCTAAGTTTCGAGCTGGACTCAGTGtcttttctcaatgacaacggc
CCTCCTAAGTTTCGAGCTGGACTCAGTGacgatttcgtttcattattcc
CCTCCTAAGTTTCGAGCTGGACTCAGTGcatttggtgattcgtttcttg

Can-elt-3B-specific (probeset2): 16 probes

CCTCCTAAGTTTCGAGCTGGACTCAGTGccgagaggactttgagtatt
CCTCCTAAGTTTCGAGCTGGACTCAGTGagtttgggtagtaggtttga
CCTCCTAAGTTTCGAGCTGGACTCAGTGtgtatcagtgcttgaatgcg
CCTCCTAAGTTTCGAGCTGGACTCAGTGtcatgagtaacttaccacgt
CCTCCTAAGTTTCGAGCTGGACTCAGTGgtatcacttggaattggtgt
CCTCCTAAGTTTCGAGCTGGACTCAGTGtgatgataatttgctggcga
CCTCCTAAGTTTCGAGCTGGACTCAGTGtgtgattctgattttgttga
CCTCCTAAGTTTCGAGCTGGACTCAGTGgcacttgattatgattctga
CCTCCTAAGTTTCGAGCTGGACTCAGTGgttgctcaaattgatgatct
CCTCCTAAGTTTCGAGCTGGACTCAGTGcttggtggtgatgatattct
CCTCCTAAGTTTCGAGCTGGACTCAGTGtgtggtggtagttgatggtg
CCTCCTAAGTTTCGAGCTGGACTCAGTGtgaactatttgttggctcga
CCTCCTAAGTTTCGAGCTGGACTCAGTGtccgttgttgatggatatgg
CCTCCTAAGTTTCGAGCTGGACTCAGTGagtaaccggagcttgtattg
CCTCCTAAGTTTCGAGCTGGACTCAGTGctggaaggggagcagcagag
CCTCCTAAGTTTCGAGCTGGACTCAGTGcttccttcattaaatgagcg

Can-elt-2: 24 probes

CCTCCTAAGTTTCGAGCTGGACTCAGTGggattgggagctgtaaataac
CCTCCTAAGTTTCGAGCTGGACTCAGTGaacgcgatagtcgttcattga
CCTCCTAAGTTTCGAGCTGGACTCAGTGccaacggattggcaatctttt
CCTCCTAAGTTTCGAGCTGGACTCAGTGtcggtataaattggttgctca
CCTCCTAAGTTTCGAGCTGGACTCAGTGgattgagcatcggagcatttg
CCTCCTAAGTTTCGAGCTGGACTCAGTGcatcgggtttagcatatcttg
CCTCCTAAGTTTCGAGCTGGACTCAGTGccatgtagttggtggagaaat
CCTCCTAAGTTTCGAGCTGGACTCAGTGtgttggtgggagaattgtgtg

Development • Supplementary information

CCTCCTAAGTTTCGAGCTGGACTCAGTGtctagcaaattgagttgcaca
CCTCCTAAGTTTCGAGCTGGACTCAGTGacgagttgaatgtggagaagc
CCTCCTAAGTTTCGAGCTGGACTCAGTGattaatggtagagctggatca
CCTCCTAAGTTTCGAGCTGGACTCAGTGttgttgagaaggtgactggtg
CCTCCTAAGTTTCGAGCTGGACTCAGTGgttgaatgtggcatcacataa
CCTCCTAAGTTTCGAGCTGGACTCAGTGatcttgtggaggtggtgtaaa
CCTCCTAAGTTTCGAGCTGGACTCAGTGgtttttgttcagcgaccaatg
CCTCCTAAGTTTCGAGCTGGACTCAGTGagccattcgctttttgacaag
CCTCCTAAGTTTCGAGCTGGACTCAGTGaattttgatgacattgcacgg
CCTCCTAAGTTTCGAGCTGGACTCAGTGtgtcttgcagtttgagcaaat
CCTCCTAAGTTTCGAGCTGGACTCAGTGcatttcgtcgccaaagtgtag
CCTCCTAAGTTTCGAGCTGGACTCAGTGtacaaattgcaagcgttgcac
CCTCCTAAGTTTCGAGCTGGACTCAGTGtttttcgattgtttttgcgga
CCTCCTAAGTTTCGAGCTGGACTCAGTGtcttttctcaatgacaacggc
CCTCCTAAGTTTCGAGCTGGACTCAGTGacgatttcgtttcattattcc
CCTCCTAAGTTTCGAGCTGGACTCAGTGcatttggtgattcgtttcttg

Can-elt-5: 24 probes

CCTCCTAAGTTTCGAGCTGGACTCAGTGtgatgatgactcgatttgtgtc
CCTCCTAAGTTTCGAGCTGGACTCAGTGtgttaattcgggagtttcagcg
CCTCCTAAGTTTCGAGCTGGACTCAGTGttgatttcgtggtgcaattgta
CCTCCTAAGTTTCGAGCTGGACTCAGTGgaatttatccatcatttgttgc
CCTCCTAAGTTTCGAGCTGGACTCAGTGctctcaagtctcatatacatca
CCTCCTAAGTTTCGAGCTGGACTCAGTGtccttctctttatcactgataa
CCTCCTAAGTTTCGAGCTGGACTCAGTGcacgattaatcgattctttggt
CCTCCTAAGTTTCGAGCTGGACTCAGTGagaaattttccgaattctgcgc
CCTCCTAAGTTTCGAGCTGGACTCAGTGtgtcgtttctcatctgataatc
CCTCCTAAGTTTCGAGCTGGACTCAGTGgaaatggtgaactagccgagac
CCTCCTAAGTTTCGAGCTGGACTCAGTGgatcgaacataaatccgtttgc
CCTCCTAAGTTTCGAGCTGGACTCAGTGtattcatcatttgttgttggtg
CCTCCTAAGTTTCGAGCTGGACTCAGTGggtttcttggagtttttgtttg
CCTCCTAAGTTTCGAGCTGGACTCAGTGcgatggtgatgttttgatgttg
CCTCCTAAGTTTCGAGCTGGACTCAGTGtgaattgttggtggatggtgtg
CCTCCTAAGTTTCGAGCTGGACTCAGTGatcttcatcattattcgaagcg
CCTCCTAAGTTTCGAGCTGGACTCAGTGtttggtcgtcatacaattcgag
CCTCCTAAGTTTCGAGCTGGACTCAGTGgatgagtccgatgtaatcgata
CCTCCTAAGTTTCGAGCTGGACTCAGTGtgttgaatgaaatctttgcgca
CCTCCTAAGTTTCGAGCTGGACTCAGTGtgcgttttgatttgatacatca
CCTCCTAAGTTTCGAGCTGGACTCAGTGcatggctagaagttgactgaac
CCTCCTAAGTTTCGAGCTGGACTCAGTGatgagaaggcgtttggtgtatt
CCTCCTAAGTTTCGAGCTGGACTCAGTGtgagttgattgaattgctccaa
CCTCCTAAGTTTCGAGCTGGACTCAGTGtattcaattgctcctgaacttg

Can-elt-1: 24 probes

CCTCCTAAGTTTCGAGCTGGACTCAGTGcgcaatttacacactctcgg

Development • Supplementary information

CCTCCTAAGTTTCGAGCTGGACTCAGTGccaaagtggcgtgttttgaa
CCTCCTAAGTTTCGAGCTGGACTCAGTGgcaaagataatgtccgccgc
CCTCCTAAGTTTCGAGCTGGACTCAGTGgtgatataatccacaggcat
CCTCCTAAGTTTCGAGCTGGACTCAGTGccgattttgtccgttcattt
CCTCCTAAGTTTCGAGCTGGACTCAGTGctttttcggcttcaccaacg
CCTCCTAAGTTTCGAGCTGGACTCAGTGtcgtttttgagtgctctgtc
CCTCCTAAGTTTCGAGCTGGACTCAGTGatttacacatgatacgccgg
CCTCCTAAGTTTCGAGCTGGACTCAGTGtgttgttgtgttcgttttgc
CCTCCTAAGTTTCGAGCTGGACTCAGTGggaaccatttcgtcgccaaa
CCTCCTAAGTTTCGAGCTGGACTCAGTGtgcattacaaactggctctc
CCTCCTAAGTTTCGAGCTGGACTCAGTGtcaacctgatgcagcttata
CCTCCTAAGTTTCGAGCTGGACTCAGTGtttttcatcgtcaatggtcg
CCTCCTAAGTTTCGAGCTGGACTCAGTGcgatttctcgtctgaattcc
CCTCCTAAGTTTCGAGCTGGACTCAGTGcctccttttgttgtcaattt
CCTCCTAAGTTTCGAGCTGGACTCAGTGcaaatccatttttcccatcg
CCTCCTAAGTTTCGAGCTGGACTCAGTGagcagctgcatttacagatg
CCTCCTAAGTTTCGAGCTGGACTCAGTGcttcattccccaaacagtgg
CCTCCTAAGTTTCGAGCTGGACTCAGTGcattggttgtgttgttgaag
CCTCCTAAGTTTCGAGCTGGACTCAGTGtcctcctgatgatgtcatta
CCTCCTAAGTTTCGAGCTGGACTCAGTGattatttgtggcgaatggat
CCTCCTAAGTTTCGAGCTGGACTCAGTGtcatcgatttgtggggtgaa
CCTCCTAAGTTTCGAGCTGGACTCAGTGtccatgattggaattgatga
CCTCCTAAGTTTCGAGCTGGACTCAGTGcaggtcgtgcaataattgct

Cel-skn-1: 19 probes

CCTCCTAAGTTTCGAGCTGGACTCAGTGaatgtaggcgtagttggatgtt
CCTCCTAAGTTTCGAGCTGGACTCAGTGttgaagactgtcgttttgcatt
CCTCCTAAGTTTCGAGCTGGACTCAGTGaatctgtccatttgatacaact
CCTCCTAAGTTTCGAGCTGGACTCAGTGgtggattgataggaatgatcat
CCTCCTAAGTTTCGAGCTGGACTCAGTGagaggagacagtggagtctgac
CCTCCTAAGTTTCGAGCTGGACTCAGTGttgacgtcctgaagatccaatg
CCTCCTAAGTTTCGAGCTGGACTCAGTGaacgattgagtagctgttgcag
CCTCCTAAGTTTCGAGCTGGACTCAGTGctctgtgagtgatatggatcga
CCTCCTAAGTTTCGAGCTGGACTCAGTGgtagtgcaatcactaaacgagt
CCTCCTAAGTTTCGAGCTGGACTCAGTGcgagagcacgttgatgacgaat
CCTCCTAAGTTTCGAGCTGGACTCAGTGtatcgtggagattccgaagaga
CCTCCTAAGTTTCGAGCTGGACTCAGTGaactttccgtagaaacgagact
CCTCCTAAGTTTCGAGCTGGACTCAGTGtgatgaacgtggagacgatgat
CCTCCTAAGTTTCGAGCTGGACTCAGTGaacaactctcgcgatcttaatc
CCTCCTAAGTTTCGAGCTGGACTCAGTGaatgacatctccgaaatctgga
CCTCCTAAGTTTCGAGCTGGACTCAGTGttcttcaacacttgttgcaact
CCTCCTAAGTTTCGAGCTGGACTCAGTGtctttgatactcgctgagactc
CCTCCTAAGTTTCGAGCTGGACTCAGTGtcgacgaatcttgcgaatcaac
CCTCCTAAGTTTCGAGCTGGACTCAGTGatgatgtaatgggacatcttgt

Development • Supplementary information

Cel-elt-3: 18 probes

CCTCCTAAGTTTCGAGCTGGACTCAGTGtttttccactcgataatcgttc
CCTCCTAAGTTTCGAGCTGGACTCAGTGtatggatctagtagtgtgttgg
CCTCCTAAGTTTCGAGCTGGACTCAGTGtaggtcggttggtcaagttgaa
CCTCCTAAGTTTCGAGCTGGACTCAGTGcagtacctgtgcattagtgaag
CCTCCTAAGTTTCGAGCTGGACTCAGTGaagttcatctggagcatttctt
CCTCCTAAGTTTCGAGCTGGACTCAGTGtaacttgtggataatggtgttg
CCTCCTAAGTTTCGAGCTGGACTCAGTGtgtgtgacttgtgctgtattca
CCTCCTAAGTTTCGAGCTGGACTCAGTGatattgaacggcatttgatgtg
CCTCCTAAGTTTCGAGCTGGACTCAGTGtgttgcgaagtttgaaaggttc
CCTCCTAAGTTTCGAGCTGGACTCAGTGgtgagctgttgagaagtggaag
CCTCCTAAGTTTCGAGCTGGACTCAGTGtttgacattgttgtgtatggat
CCTCCTAAGTTTCGAGCTGGACTCAGTGcattcttttttcatcggcttc
CCTCCTAAGTTTCGAGCTGGACTCAGTGattttgatgacattgtacagct
CCTCCTAAGTTTCGAGCTGGACTCAGTGtgttttgcaatttgagcagatg
CCTCCTAAGTTTCGAGCTGGACTCAGTGgtttttccggaagtagagattg
CCTCCTAAGTTTCGAGCTGGACTCAGTGtaagagacagtggacgttttcg
CCTCCTAAGTTTCGAGCTGGACTCAGTGttctcttcataattccatcttt
CCTCCTAAGTTTCGAGCTGGACTCAGTGgagtttggagactcatttctag

Cel-end-3: 20 probes

CCTCCTAAGTTTCGAGCTGGACTCAGTGgaagagaatgagttcgagtaca
CCTCCTAAGTTTCGAGCTGGACTCAGTGaagacattggtgagttggaaga
CCTCCTAAGTTTCGAGCTGGACTCAGTGgggaaactgagggaatccaaaa
CCTCCTAAGTTTCGAGCTGGACTCAGTGttcttcattgaattggacttgt
CCTCCTAAGTTTCGAGCTGGACTCAGTGatattctggaggacatctggag
CCTCCTAAGTTTCGAGCTGGACTCAGTGttgtgccggataatgatgataa
CCTCCTAAGTTTCGAGCTGGACTCAGTGcattgtatgaatttgtcgtcat
CCTCCTAAGTTTCGAGCTGGACTCAGTGctgttgcattgaattgtcatag
CCTCCTAAGTTTCGAGCTGGACTCAGTGaccattatctggttgcataaaa
CCTCCTAAGTTTCGAGCTGGACTCAGTGgatagttttcattgtagtatcc
CCTCCTAAGTTTCGAGCTGGACTCAGTGattaataggcgtcgtgaattca
CCTCCTAAGTTTCGAGCTGGACTCAGTGttgatggaagtttgagattgct
CCTCCTAAGTTTCGAGCTGGACTCAGTGcatggaattttcattgttgtcg
CCTCCTAAGTTTCGAGCTGGACTCAGTGcttgaacttcttgagctggaaa
CCTCCTAAGTTTCGAGCTGGACTCAGTGtatttttcgaagcttttctagc
CCTCCTAAGTTTCGAGCTGGACTCAGTGatgcattgtggaaatcttcttg
CCTCCTAAGTTTCGAGCTGGACTCAGTGacaattggagcacgaagagttg
CCTCCTAAGTTTCGAGCTGGACTCAGTGtcgttcgtaaagattacacgga
CCTCCTAAGTTTCGAGCTGGACTCAGTGaagatgttgtggtcttttatgt
CCTCCTAAGTTTCGAGCTGGACTCAGTGtagagtcaacaagtggagcaac

Cel-end-1: 20 probes

CCTCCTAAGTTTCGAGCTGGACTCAGTGtggggagatggagatgaagaag

CCTCCTAAGTTTCGAGCTGGACTCAGTGatgactccataggtatatggaa
CCTCCTAAGTTTCGAGCTGGACTCAGTGtttcatgggaatgaacatttcc
CCTCCTAAGTTTCGAGCTGGACTCAGTGtttccatgattatccggaaaat
CCTCCTAAGTTTCGAGCTGGACTCAGTGtacatttgtgtagcatcgagtg
CCTCCTAAGTTTCGAGCTGGACTCAGTGtcaaaatattgatctccaccac
CCTCCTAAGTTTCGAGCTGGACTCAGTGgattgaacaaagtattgcccaa
CCTCCTAAGTTTCGAGCTGGACTCAGTGtatcgagacttccaaacattgg
CCTCCTAAGTTTCGAGCTGGACTCAGTGgatactgttgtgagtagcaatt
CCTCCTAAGTTTCGAGCTGGACTCAGTGtgttgatagtcttgtccaattt
CCTCCTAAGTTTCGAGCTGGACTCAGTGgactggtggaatattttcgatt
CCTCCTAAGTTTCGAGCTGGACTCAGTGgttcacaattttgcgatttgta
CCTCCTAAGTTTCGAGCTGGACTCAGTGagagttcgtgtggaacgttgag
CCTCCTAAGTTTCGAGCTGGACTCAGTGtcacgagttctacaatttggat
CCTCCTAAGTTTCGAGCTGGACTCAGTGgaatcagttcttctccatagag
CCTCCTAAGTTTCGAGCTGGACTCAGTGatttttttcggaagtacagggag
CCTCCTAAGTTTCGAGCTGGACTCAGTGttttcattattgttttacggca
CCTCCTAAGTTTCGAGCTGGACTCAGTGtggtgttggagattgtacttcg
CCTCCTAAGTTTCGAGCTGGACTCAGTGattatgacatagtttcgagtct
CCTCCTAAGTTTCGAGCTGGACTCAGTGtgtgatggaatgttctgtagtg

Cel-elt-2: 24 probes

CCTCCTAAGTTTCGAGCTGGACTCAGTGcagccgttgacattatcatt
CCTCCTAAGTTTCGAGCTGGACTCAGTGcattattttgctctggtgga
CCTCCTAAGTTTCGAGCTGGACTCAGTGtttgccgttcaattggagat
CCTCCTAAGTTTCGAGCTGGACTCAGTGtagttatttgtgccacttgt
CCTCCTAAGTTTCGAGCTGGACTCAGTGtgagtagtctatgccagtat
CCTCCTAAGTTTCGAGCTGGACTCAGTGgtttggtccaacattccaaa
CCTCCTAAGTTTCGAGCTGGACTCAGTGagtgtttacgggaattccac
CCTCCTAAGTTTCGAGCTGGACTCAGTGtggagtttgtatatcccgaa
CCTCCTAAGTTTCGAGCTGGACTCAGTGgaatactggggtcgtacaga
CCTCCTAAGTTTCGAGCTGGACTCAGTGgccacagttggataagtaga
CCTCCTAAGTTTCGAGCTGGACTCAGTGtgtgagcatttgacgcattc
CCTCCTAAGTTTCGAGCTGGACTCAGTGgtgttgaacagttgacgcac
CCTCCTAAGTTTCGAGCTGGACTCAGTGactggaggagaatacgtggt
CCTCCTAAGTTTCGAGCTGGACTCAGTGttttagaggattgcttggca
CCTCCTAAGTTTCGAGCTGGACTCAGTGcaattggagcacacaagtcc
CCTCCTAAGTTTCGAGCTGGACTCAGTGcatttcttctccagagagtt
CCTCCTAAGTTTCGAGCTGGACTCAGTGggagtttgaagtaaagcccg
CCTCCTAAGTTTCGAGCTGGACTCAGTGctgtaaagcaccttctttct
CCTCCTAAGTTTCGAGCTGGACTCAGTGcgttgatggtgtggaagagt
CCTCCTAAGTTTCGAGCTGGACTCAGTGtctctcaaacttcctttctc
CCTCCTAAGTTTCGAGCTGGACTCAGTGcggcatgtgacacatatgtg
CCTCCTAAGTTTCGAGCTGGACTCAGTGtactgtacgtttgatctggc
CCTCCTAAGTTTCGAGCTGGACTCAGTGttgttgctgcatacacgtag
CCTCCTAAGTTTCGAGCTGGACTCAGTGctggcatcacatgaacttgg

Development • Supplementary information

Cpo-elt-3: 22 probes

CCTCCTAAGTTTCGAGCTGGACTCAGTGtgtcgagtagaagctgttca
CCTCCTAAGTTTCGAGCTGGACTCAGTGcggattctgtagattgtggg
CCTCCTAAGTTTCGAGCTGGACTCAGTGattccagtcattgtagttgg
CCTCCTAAGTTTCGAGCTGGACTCAGTGcttgctgatccgaatttgtt
CCTCCTAAGTTTCGAGCTGGACTCAGTGgcaaattcggagctgtttga
CCTCCTAAGTTTCGAGCTGGACTCAGTGagttatcagtgttgttctgc
CCTCCTAAGTTTCGAGCTGGACTCAGTGggaaatcgctggcaatggag
CCTCCTAAGTTTCGAGCTGGACTCAGTGacttggagttccttcatgaa
CCTCCTAAGTTTCGAGCTGGACTCAGTGtccagctgtataatagtcgt
CCTCCTAAGTTTCGAGCTGGACTCAGTGtttttcaacgcgatagtcgc
CCTCCTAAGTTTCGAGCTGGACTCAGTGggttgttccaattgagtgta
CCTCCTAAGTTTCGAGCTGGACTCAGTGtggtgtgttggtaaattccg
CCTCCTAAGTTTCGAGCTGGACTCAGTGcatgtggttcatcatgtctt
CCTCCTAAGTTTCGAGCTGGACTCAGTGgttactggcattactgtcat
CCTCCTAAGTTTCGAGCTGGACTCAGTGagggaatgctgagaggttga
CCTCCTAAGTTTCGAGCTGGACTCAGTGgctcgcaaactgatgtgttg
CCTCCTAAGTTTCGAGCTGGACTCAGTGaaggcgattgatgcatcagt
CCTCCTAAGTTTCGAGCTGGACTCAGTGttgaatgtgcagccattacg
CCTCCTAAGTTTCGAGCTGGACTCAGTGctaatggatcttgtggtggt
CCTCCTAAGTTTCGAGCTGGACTCAGTGttttgatggcattggacagc
CCTCCTAAGTTTCGAGCTGGACTCAGTGgcaatgagagtggtcgtttg
CCTCCTAAGTTTCGAGCTGGACTCAGTGatttctcttcatgatgccat

Cpo-elt-2: 22 probes

CCTCCTAAGTTTCGAGCTGGACTCAGTGaatttgtcctccattcacat
CCTCCTAAGTTTCGAGCTGGACTCAGTGatgtgatgtaggttgttcgt
CCTCCTAAGTTTCGAGCTGGACTCAGTGgtgtttcaatgattggcgga
CCTCCTAAGTTTCGAGCTGGACTCAGTGcttattaccagacttggtgg
CCTCCTAAGTTTCGAGCTGGACTCAGTGttgatgaattacccgatccg
CCTCCTAAGTTTCGAGCTGGACTCAGTGgaacacaccaatccttgacg
CCTCCTAAGTTTCGAGCTGGACTCAGTGctccacagagttgtatttgt
CCTCCTAAGTTTCGAGCTGGACTCAGTGgcagcttgtagtaaagacca
CCTCCTAAGTTTCGAGCTGGACTCAGTGgaccttctttcttcatcgaa
CCTCCTAAGTTTCGAGCTGGACTCAGTGtttcctttttctagtttgca
CCTCCTAAGTTTCGAGCTGGACTCAGTGgagttgagtgtgtttcactc
CCTCCTAAGTTTCGAGCTGGACTCAGTGttcttttgtgcgggaacttg
CCTCCTAAGTTTCGAGCTGGACTCAGTGgcagcagtagatccatcaaa
CCTCCTAAGTTTCGAGCTGGACTCAGTGatgaagcatttctagcagcg
CCTCCTAAGTTTCGAGCTGGACTCAGTGgtatggtgtttcttgaagcc
CCTCCTAAGTTTCGAGCTGGACTCAGTGcacgatgcttgttggtaagt
CCTCCTAAGTTTCGAGCTGGACTCAGTGaatatgagcgtcttgcgacg
CCTCCTAAGTTTCGAGCTGGACTCAGTGctaggcccaaatgtggtaag
CCTCCTAAGTTTCGAGCTGGACTCAGTGagacagttgtttgggaagct
CCTCCTAAGTTTCGAGCTGGACTCAGTGccggtatgacttgaagatct

Development • Supplementary information

CCTCCTAAGTTTCGAGCTGGACTCAGTGgcttttgtttcatcgtcttg
CCTCCTAAGTTTCGAGCTGGACTCAGTGgtagtgtcttccaaatctct

Can-myo-2: 22 probes

CCTCCTAAGTTTCGAGCTGGACTCAGTGaaccttttgctcattcatgg
CCTCCTAAGTTTCGAGCTGGACTCAGTGaagagcgcggatttgatgtt
CCTCCTAAGTTTCGAGCTGGACTCAGTGtgcaagtcttcgctcaattt
CCTCCTAAGTTTCGAGCTGGACTCAGTGcttcaagcgattgcatgagt
CCTCCTAAGTTTCGAGCTGGACTCAGTGttgagcaatcttgagctctc
CCTCCTAAGTTTCGAGCTGGACTCAGTGttgttcgtcttccaatttca
CCTCCTAAGTTTCGAGCTGGACTCAGTGctcatcagccaattgatctt
CCTCCTAAGTTTCGAGCTGGACTCAGTGatctatcagcacgttgacga
CCTCCTAAGTTTCGAGCTGGACTCAGTGagctcatcgaattcagcttg
CCTCCTAAGTTTCGAGCTGGACTCAGTGttgatcttcgagttgctcat
CCTCCTAAGTTTCGAGCTGGACTCAGTGcttcttccaaatcacgacgg
CCTCCTAAGTTTCGAGCTGGACTCAGTGtgagttgctctccgaattta
CCTCCTAAGTTTCGAGCTGGACTCAGTGtgcaattgttcgacttggtc
CCTCCTAAGTTTCGAGCTGGACTCAGTGtttttcgatgcggttctttt
CCTCCTAAGTTTCGAGCTGGACTCAGTGagaagtctgacttcatgtcc
CCTCCTAAGTTTCGAGCTGGACTCAGTGttcgtcagccttaagacgaa
CCTCCTAAGTTTCGAGCTGGACTCAGTGtcaaacgtcccttggatgaa
CCTCCTAAGTTTCGAGCTGGACTCAGTGtagcgtgatccaattcgttg
CCTCCTAAGTTTCGAGCTGGACTCAGTGcttggacaagttcgagaggt
CCTCCTAAGTTTCGAGCTGGACTCAGTGccttgagttgttcaagttca
CCTCCTAAGTTTCGAGCTGGACTCAGTGttggagttgacgagtagctt
CCTCCTAAGTTTCGAGCTGGACTCAGTGattcatcggctccaataagt

Can-eft-3 (eef1A.1): 16 probes

CCTCCTAAGTTTCGAGCTGGACTCAGTGgcgtacttgaacgaaccttt
CCTCCTAAGTTTCGAGCTGGACTCAGTGcaatggtgataccacgttca
CCTCCTAAGTTTCGAGCTGGACTCAGTGcctggagcatcaataatggt
CCTCCTAAGTTTCGAGCTGGACTCAGTGcttgacaccaagagtttggg
CCTCCTAAGTTTCGAGCTGGACTCAGTGttgttgcaggcaacgatcaa
CCTCCTAAGTTTCGAGCTGGACTCAGTGgaaacgggcctcagagaatg
CCTCCTAAGTTTCGAGCTGGACTCAGTGccgatcttcttgatgaatcc
CCTCCTAAGTTTCGAGCTGGACTCAGTGacgaatggaacagcctttgg
CCTCCTAAGTTTCGAGCTGGACTCAGTGaacttcgagcatgttgtctc
CCTCCTAAGTTTCGAGCTGGACTCAGTGgacgtcttggagtggaagac
CCTCCTAAGTTTCGAGCTGGACTCAGTGacttcagtggtgacgttttg
CCTCCTAAGTTTCGAGCTGGACTCAGTGtgacgttgaatccgacgttg
CCTCCTAAGTTTCGAGCTGGACTCAGTGatgtccttgacggaaacgtt
CCTCCTAAGTTTCGAGCTGGACTCAGTGtttcgagtcagagcagactg
CCTCCTAAGTTTCGAGCTGGACTCAGTGagaactggagtgtaaccagc
CCTCCTAAGTTTCGAGCTGGACTCAGTGccttgagctcgttgaatttg

Development • Supplementary information

5) CRISPR/Cas9 PCR primers and sequence of *ir79* allele

Can-elt-3 crRNA targeting site 1 :gtgcttgaatgcggtgagtttgg
Can-elt-3 crRNA targeting site 2: gaatttctccaccaactacatgg

Primers used to identify deletion mutant:

Elt-3CC1F: 5’-ctgactgttttgaggtctgctcaaaaacaac-3’
Elt-3CC1B: 5’-caaattgaattttgatgacattgcacggc-3’

Sequence of *ir79* deletion:

tctggtagctcacttgctcctgggaaggtagcagtactatatatagtgcatgcgcaattcattacctccctctttcattaaaaataatcttaaa
tattttttttgttgatgatagataattttttttgtagtatttgctttttactgactgttttgaggtctgctcaaaaacaacaaaactgtttttttctccattttt
ggtaccttttcgaaatcgatcgtttattatcagttgttttttcagacactaacaacaaaatgaatactcaaagtcctctcgggattcaaaccta
ctaccca

(DELETION, 2916 base pairs)

tgggagccacacaattctcccaccaacagactgtgcaactcaatttgctagatctcagcggcttctccacattcaactcgtgtgatccag
ctctaccattaatgcaccagtcaccttctcaacaaaattatgtgatgccacattcaacttttacaccacctccacaagatccattggtcgct
gaacaaaaaccacttgtcaaaaagcgaatggctgtaagttttgtttttttttttttgaaaacgggttttattgattttccttgttgacagaaaaca
gtgatttcagaaaaacaaagag

Development • Supplementary information
